# Facile synthesis of functionalized spiro[indoline-3,2'-oxiran]-2-ones by Darzens reaction

**DOI:** 10.3762/bjoc.9.105

**Published:** 2013-05-13

**Authors:** Qin Fu, Chao-Guo Yan

**Affiliations:** 1 College of Chemistry & Chemical Engineering, Yangzhou University, Yangzhou 225002, China

**Keywords:** Darzens reaction, isatin, oxindole, oxirane, spiro-epoxyoxindole, spirooxindole

## Abstract

A series of functionalized spiro[indoline-3,2'-oxiran]-2-ones was efficiently synthesized by Darzens reaction of phenacyl bromides with isatins both with *N*-alkyl groups and without *N*-substituent in the presence of potassium carbonate as a base catalyst. When two equivalents phenacyl bromides were used in the reaction, the *N*-substitution reaction of isatin also finished with the formation of spiro-oxirane-oxindoles.

## Introduction

The spirooxindole unit is a privileged heterocyclic motif that forms the core structure of a large family of natural alkaloids and many pharmacological agents with important bioactivity and interesting structural properties [1–5]. The unique structures and the highly pronounced pharmacological activity displayed by the spirooxindoles have made them attractive synthetic targets [6–9]. In various heterocyclic and carbocyclic spirooxindoles, the spiro-oxirane-oxindoles are a particular class of compounds with both spiro-carbon and unstable oxirane features in the molecule. These fascinating spiranic frameworks can serve as important building blocks in organic synthesis for the synthesis of large-ring heterocycles [10–13]. As a consequence, in recent years much attention has been paid to the diastereoselective and enantioselective synthesis of versatile spiro-oxirane-oxindoles [14–19]. With the aim of expanding our previous studies on the synthesis of various spirooxindoles [20–25], we decided to systematically investigate the Darzens reactions of a series of isatins with phenacyl bromides and report the facile synthesis of versatile spiro[indoline-3,2'-oxiran]-2-ones.

## Results and Discussion

**Scheme 1:**
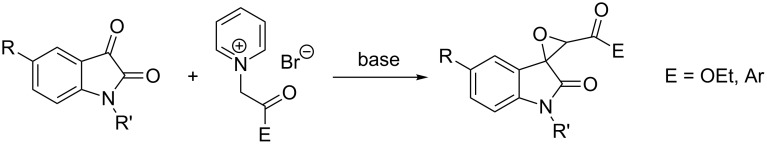
Synthesis of spiro-epoxyoxindole with pyridinium ylide.

In recent years we have found that pyridinium salts react with versatile reactive methylene compounds to give different kinds of products, including functionalized cyclopropanes, 2,3-dihydrofurans, polysubstituted pyridines, pyrido[1,2-*a*]benzimidazoles and charge-separated zwitterionic salts [26–31]. We envisaged that in situ generated pyridinium ylide might react with the reactive carbonyl group of isatins to afford spiro epoxyoxindoles (Scheme 1). To test this feasibility, the reactions of various substituted isatins with pyridinium salt in the presence of base were examined under different conditions. We were disappointed that the reactions produced much complicated mixtures and no acceptable results were obtained. Thus, our attention was turned toward the development of straightforward reactions of phenacyl bromides with isatins.

In a preliminary experiment, a model between 5-methylisatin (**1**) and phenacyl bromide (**2**) was examined under a broad set of conditions (Table 1). A careful screening of bases revealed that potassium afforded the product in better yields. The main problem is that the *N-*alkylated spiro epoxyoxindole **4** is accompanied by the formation of spiro epoxyoxindole **3** even if equivalent reactants are used, which is consistent with the recently reported reactions of isatins with alkylating agents having an acidic methylene group by Blanco et al. [19]. To our delight, the spiro epoxyoxindole **3** could be selectively obtained in 85% yield when the reaction was carried out in the system of K_2_CO_3_/CHCl_3_ at about 50 °C for 10 h. On the other hand the *N-*alkylated spiro epoxyoxindole **4** was also successfully prepared in 90% yield in this system when more than two equivalents of phenacyl bromide were utilized.

**Table 1:** Reaction of 5-methylisatin (**1**) with phenacyl bromide (**2**).

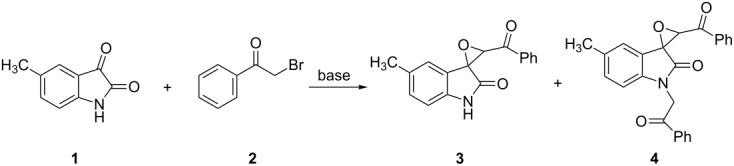							

Entry	Base	Ratios of **1**/**2**	Solvent	Temp. (°C)	Time (h)	Yield (%)	

						3	4

1	NEt_3_	1:1	EtOH	10–15	24	—	—
2	DABCO	1:1	EtOH	10–15	18	40	—
3	DBU	1:1	EtOH	10–15	18	62	—
4	K_2_CO_3_	1:1	EtOH	10–15	18	70	—
5	K_2_CO_3_	1:1	CHCl_3_	10–15	18	78	—
6	K_2_CO_3_	1:1	CHCl_3_	50	10	85	—
7	K_2_CO_3_	1:1	MeCN	50	10	60	—
8	K_2_CO_3_	1:1	EtOH	50	10	56	18
9	K_2_CO_3_	1:1	toluene	50	10	87	—
10	K_2_CO_3_	1:1	DMF	50	10	28	46
11	K_2_CO_3_	1:1.2	CHCl_3_	50	10	66	15
12	K_2_CO_3_	1:2	CHCl_3_	50	10	10	77
13	K_2_CO_3_	1:2.2	CHCl_3_	50	10	—	90
14	K_2_CO_3_	1:2.2	DMF	50	10	—	89

**Figure 1:**
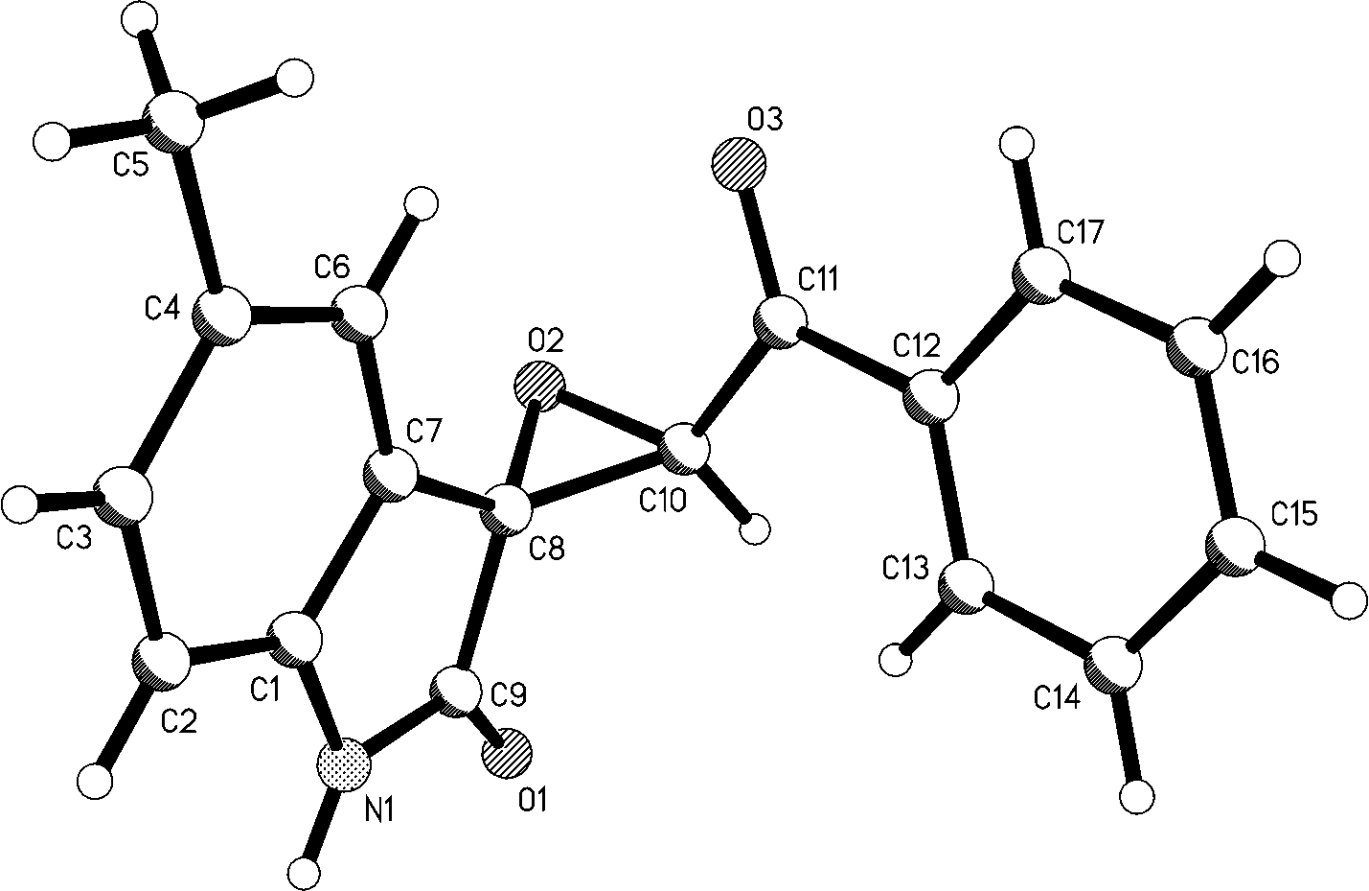
Molecular structure of spiro compound **3c**.

**Figure 2:**
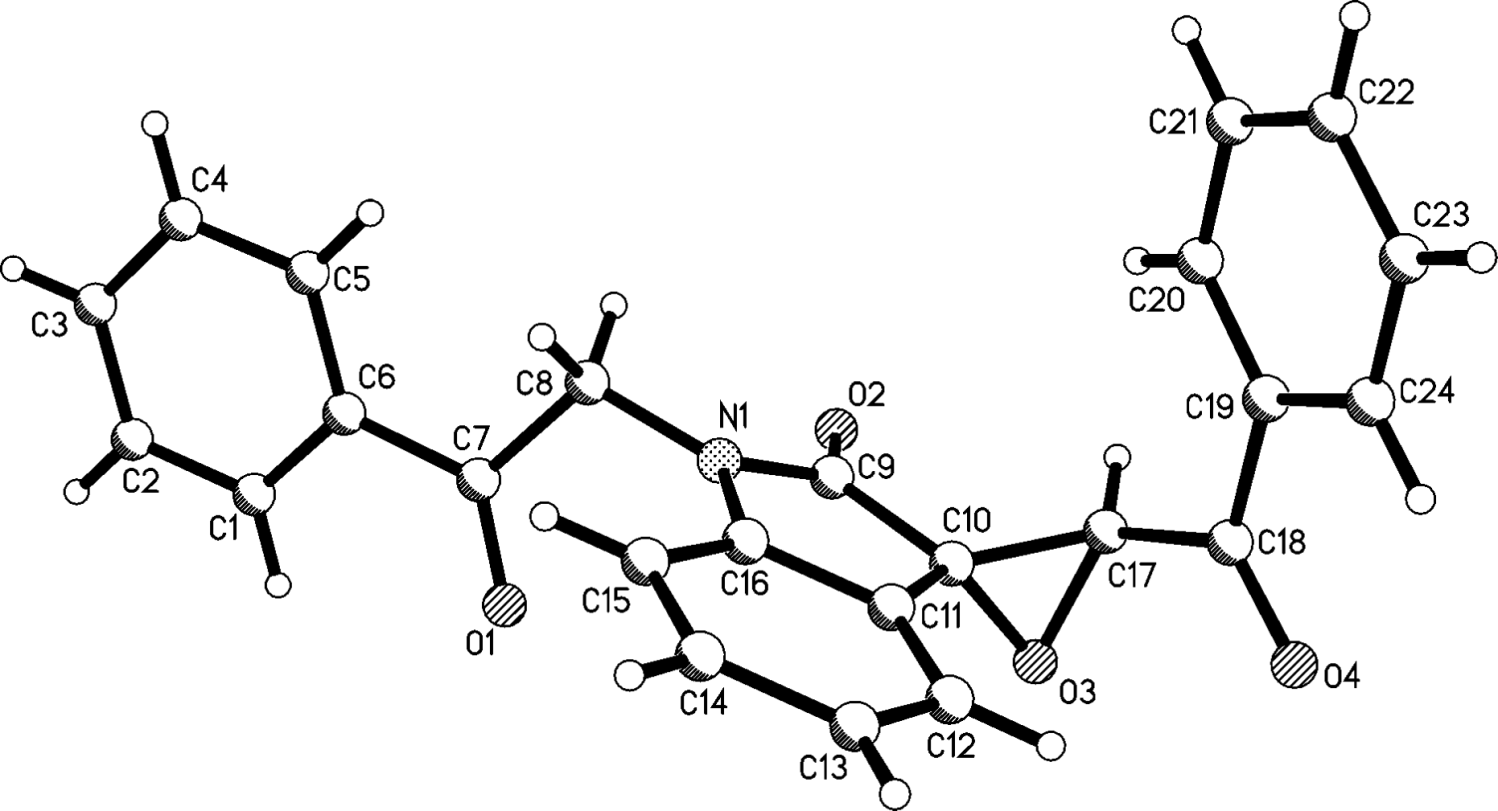
Molecular structure of spiro compound **4a**.

After obtaining the optimized reaction conditions, various substituted isatins and phenacyl bromides were employed in the reaction. The results are summarized in Table 2. All reactions proceeded very smoothly and eight spiro epoxyoxindoles **3a**–**h** were obtained in satisfactory yields. Similarly the *N*-substituted spiro epoxyoxindoles **4a**–**e** were synthesized in high yields by using an excess of phenacyl bromide. The results are summarized in Table 3. The structures of compounds **3a**–**h** and **4a**–**e** were characterized by IR, ^1^H, ^13^C NMR, and HRMS spectra and were further confirmed by single-crystal X-ray diffraction determination of the compound **3c** (Figure 1) and **4a** (Figure 2). It should be pointed out that some of the spiro epoxyoxindoles **3a**–**h** have been previously prepared by other methods and the related references are also listed in Table 2. The ^1^H NMR spectra of compounds **3a**–**h** and **4a**–**e** usually show one set of characteristic peaks for each group, especially one singlet at about 5.10 ppm for one proton of the epoxy unit, which clearly indicates that only one isomer exists in each sample. However, ^1^H NMR spectra of compounds **3a** and **3f** clearly displayed that the *trans*-isomer existed mainly with ratios of *cis*/*trans* isomers of 1:14 and 1:8, respectively. From Figure 1 and Figure 2 it is seen that the phenyl group of the oxindole unit and the benzoyl group existed in the *cis*-position. This result also indicated that the thermodynamic reaction produces a more stable *trans*-isomer.

**Table 2:** Synthesis of spiro[indoline-3,2'-oxiran]-2-ones **3a**–**h**.

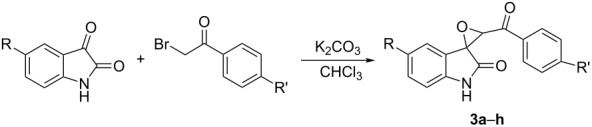					

Entry	Compound	R	R’	Yield (%, *cis*/*trans* ratio)	Ref.

1	**3a**	H	H	83 (14:1)	[32]
2	**3b**	H	Cl	72	[33]
3	**3c**	CH_3_	H	85	[32]
4	**3d**	CH_3_	Cl	80	—
5	**3e**	F	H	78	[34]
6	**3f**	F	Cl	77 (8:1)	[13]
7	**3g**	Cl	H	86	[34]
8	**3h**	Cl	Cl	82	—

**Table 3:** Synthesis of spiro[indoline-3,2'-oxiran]-2-ones **4a**–**e**.

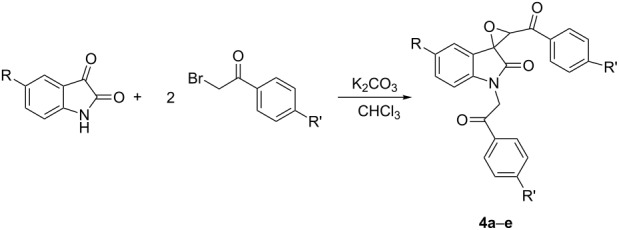				

Entry	Compound	R	R’	Yield (%)

1	**4a**	H	H	93
2	**4b**	H	Cl	89
3	**4c**	CH_3_	H	90
4	**4d**	CH_3_	Cl	85
5	**4e**	Cl	Cl	88

**Figure 3:**
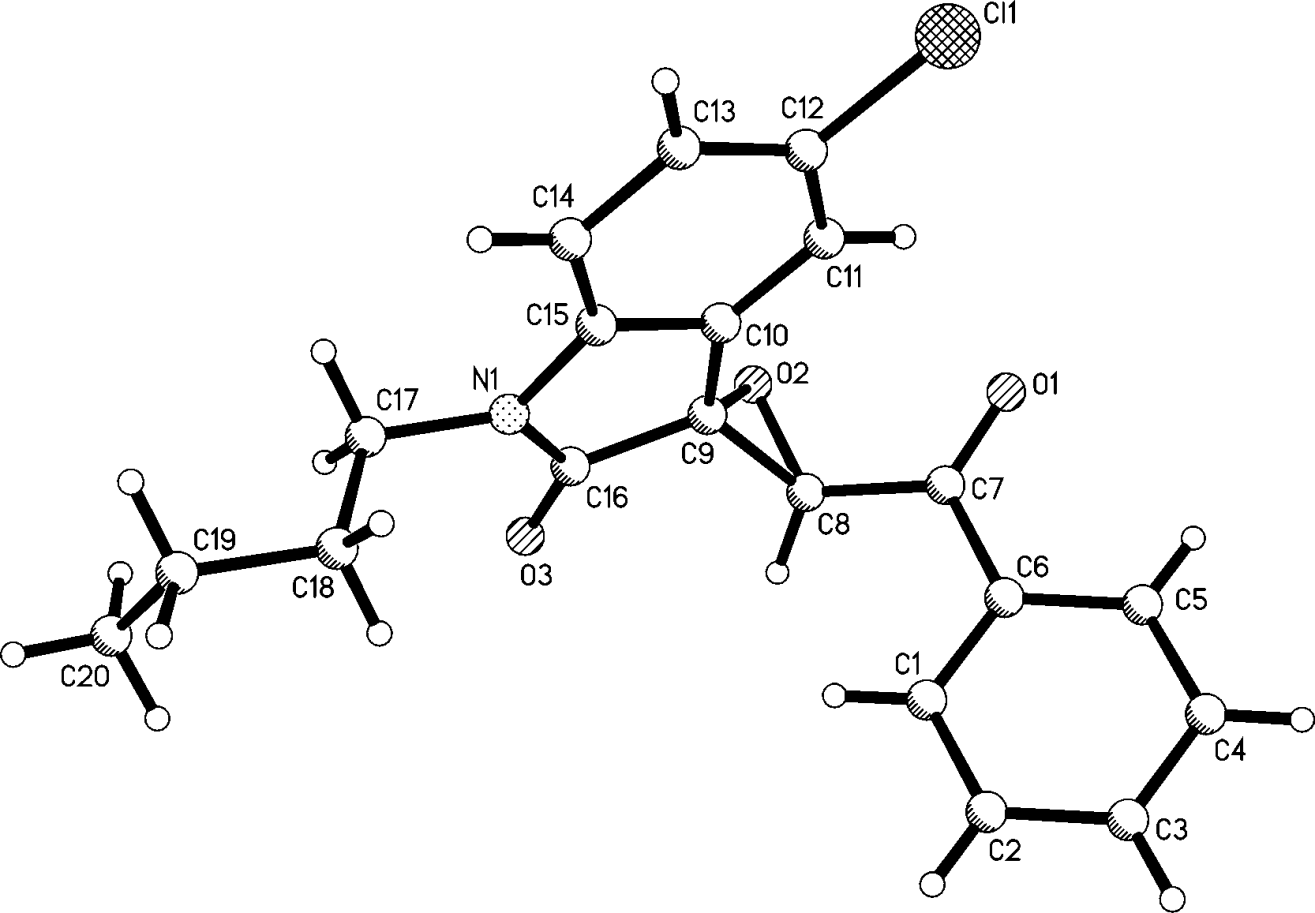
Molecular structure of spiro compound **5o**.

To further illustrate the power of this reaction procedure, the *N*-substituted isatins were also employed to react with phenacyl bromides under similar reaction conditions. A series of new spiro epoxyoxindoles **5a**–**p** were prepared in high yields (Table 4). The structures of the spiro compounds **5a**–**p** were also established by spectroscopic methods and were confirmed by single-crystal X-ray structure determination of compound **5o** (Figure 3). ^1^H NMR spectra showed that a mixture of *cis*/*trans* isomers existed in most samples with a large range of different *cis*/*trans* ratios. It is known that the closure of the epoxy ring would form *cis*/*trans* isomers in the Darzens reaction process. Here the *N*-benzyl and the *N*-butyl group in the oxindole moiety may decrease the steric effect of formation of the epoxy ring and lead to the easier formation of the relatively unstable *cis*-isomer.

**Table 4:** Synthesis of spiro[indoline-3,2'-oxiran]-2-ones **5a**–**p**.

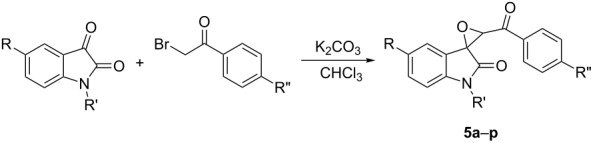					

Entry	Compound	R	R’	R”	Yield (%, *cis*/*trans* ratio)

1	**5a**	H	CH_2_Ph	H	92 [35]
2	**5b**	H	CH_2_Ph	Cl	80 (12:1)
3	**5c**	H	*n*-Bu	H	88
4	**5d**	H	*n*-Bu	Cl	76 (1:1)
5	**5e**	CH_3_	CH_2_Ph	H	90 (2:1)
6	**5f**	CH_3_	CH_2_Ph	Cl	86 (3:1)
7	**5g**	CH_3_	*n*-Bu	H	82 (4:1)
8	**5h**	CH_3_	*n*-Bu	Cl	86 (4:1)
9	**5i**	F	CH_2_Ph	H	80 (5:4)
10	**5j**	F	CH_2_Ph	Cl	89 (5:2)
11	**5k**	F	*n*-Bu	H	83 (11:1)
12	**5l**	F	*n*-Bu	Cl	92 (3:1)
13	**5m**	Cl	CH_2_Ph	H	84 (5:1)
14	**5n**	Cl	CH_2_Ph	Cl	91
15	**5o**	Cl	*n*-Bu	H	81
16	**5p**	Cl	*n*-Bu	Cl	88 (10:1)

## Conclusion

In summary, we have systematically investigated the Darzens reaction of phenacyl bromides with isatins for the efficient synthesis of the functionalized spiro epoxyoxindoles. Both the nonsubstituted isatins and *N-*alkylated isatins were successfully used in the reactions. The scope and limitation of the reaction was established. The *N*-alkylation of the isatins is usually accompanied by the formation of spiro epoxyoxindoles.

## Experimental

**Reagents and apparatus**: Melting points were taken on a hot-plate microscope apparatus. IR spectra were obtained on a Bruker Tensor 27 spectrometer (KBr disc). NMR spectra were recorded with a Bruker AV-600 spectrometer with DMSO-*d*_6_ as solvent and TMS as internal standard (600 and 150 MHz for ^1^H and ^13^C NMR spectra, respectively). HRMS were measured on a UHR-TOF maXis instrument. X-ray data were collected on a Bruker Smart APEX-2 diffractometer. Isatins, phenacyl bromide and other reagents are commercial reagents and were used as received. Solvents were purified by standard techniques. All reactions were monitored by TLC.

**Typical procedure for the preparation of spiro[indoline-3,2'-oxiran]-2-ones 3a–h:** A mixture of isatin (1.0 mmol), phenacyl bromide (1.0 mmol) and potassium carbonate (1.2 mmol) in 20.0 mL chloroform was stirred at 50 °C for 10–24 h (TLC analysis). After cooling the reaction was quenched with water. The solvent was evaporated under reduced pressure. The residue was recrystallized from ethanol to give the pure product for analysis.

**3'-benzoylspiro[indoline-3,2'-oxiran]-2-one (3a)** [32]: White solid, yield: 83%; mp 158–159 °C; IR (KBr) ν: 3457, 3180, 2972, 1735, 1676, 1620, 1597, 1469, 1335, 1219, 1041, 1013, 927, 850, 794 cm^−1^; ^1^H NMR (600 MHz, DMSO-*d*_6_) δ *cis*-isomer: 11.04 (s, 1H, NH), 7.88 (d, *J* = 7.8 Hz, 2H, ArH), 7.66 (t, *J* = 7.2 Hz, 1H, ArH), 7.53 (t, *J* = 7.8 Hz, 2H, ArH), 7.28 (t, *J* = 7.2 Hz, 1H, ArH), 6.93 (d, *J* = 7.8 Hz, 1H, ArH), 6.88 (t, *J* = 7.2 Hz, 1H, ArH), 6.82 (d, *J* = 7.8 Hz, 1H, ArH), 5.15 (s, 1H, CH); *trans*-isomer: 7.96 (d, *J* = 7.8 Hz, 2H, ArH), 7.76 (t, *J* = 7.2 Hz, 1H, ArH), 7.62 (d, *J* = 7.2 Hz, 1H, ArH), 7.55 (t, *J* = 7.8 Hz, 2H, ArH), 7.32 (d, *J* = 7.8 Hz, 1H, ArH), 7.14 (d, *J* = 7.8 Hz, 1H, ArH), 6.97 (d, *J* = 7.2 Hz, 1H, ArH), 5.28 (s, 1H, CH); ^13^C NMR (150 MHz, DMSO-*d*_6_) δ 191.0, 170.8, 144.0, 134.7, 134.4, 131.1, 129.1, 128.0, 123.2, 122.0, 119.3, 110.0, 63.6, 60.4; HRMS–ESI (*m*/*z*): [M + Na]^+^ calcd for C_16_H_11_NNaO_3_, 288.0631; found, 288.0628.

**Typical procedure for the preparation of spiro[indoline-3,2'-oxiran]-2-ones 4a**–**e:** A mixture of isatin (1.0 mmol), phenacyl bromide (2.2 mmol) and potassium carbonate (2.6 mmol) in 20.0 mL of chloroform was stirred at 50 °C for 10–24 h (TLC analysis). After cooling, the reaction was quenched with water. The solvent was evaporated under reduced pressure. The residue was recrystallized from ethanol to give the pure product for analysis.

**3'-benzoyl-1-(2-oxo-2-phenylethyl)spiro[indoline-3,2'-oxiran]-2-one (4a):** White solid, yield: 93%; mp 188–189 °C; IR (KBr) ν: 3449, 2929, 1733, 1696, 1613, 1597, 1467, 1351, 1229, 1186, 1101, 930, 786 cm^−1^; ^1^H NMR (600 MHz, DMSO-*d*_6_) δ 8.12 (d, *J* = 7.8 Hz, 2H, ArH), 7.91 (d, *J* = 7.2 Hz, 2H, ArH), 7.76 (t, *J* = 7.2 Hz, 1H, ArH), 7.69 (t, *J* = 7.2 Hz, 1H, ArH), 7.63 (t, *J* = 7.2 Hz, 2H, ArH), 7.55 (t, *J* = 7.8 Hz, 2H, ArH), 7.31 (t, *J* = 7.8 Hz, 1H, ArH), 7.14 (d, *J* = 7.8 Hz, 1H, ArH), 6.96 (t, *J* = 7.2 Hz, 1H, ArH), 6.90 (d, *J* = 7.2 Hz, 1H, ArH), 5.56–5.49 (m, 2H, CH_2_), 5.28 (s, 1H, CH); ^13^C NMR (150 MHz, DMSO-*d*_6_) δ 192.5, 190.9, 169.9, 144.9, 134.6, 134.5, 134.2, 134.1, 131.1, 129.1, 128.9, 128.3, 128.0, 122.9, 122.8, 118.7, 110.3, 64.1, 60.2, 47.3; HRMS–ESI (*m*/*z*): [M + K]^+^ calcd for C_24_H_17_KNO_4_, 422.0789; found, 422.0782.

**Typical procedure for the preparation of spiro[indoline-3,2'-oxiran]-2-ones 5a**–**p:** A mixture of isatin (1.0 mmol), phenacyl bromide (1.2 mmol) and potassium carbonate (1.5 mmol) in 20.0 mL of chloroform was stirred at 50 °C for 10–24 h (TLC analysis). After cooling, the reaction was quenched with water. The solvent was evaporated under reduced pressure. The residue was recrystallized with ethanol to give the pure product for analysis.

**3'-benzoyl-1-benzylspiro[indoline-3,2'-oxiran]-2-one (5a)** [35]**:** White solid, yield: 92%; mp 154–156 °C; IR (KBr) ν: 3034, 2923, 1730, 1691, 1610, 1463, 1360, 1232, 1185, 1104, 1007, 923, 870 cm^−1^; ^1^H NMR (600 MHz, CDCl_3_) δ 7.94 (d, *J* = 7.8 Hz, 2H, ArH), 7.61 (t, *J* = 7.2 Hz, 1H, ArH), 7.47 (t, *J* = 7.8 Hz, 2H, ArH), 7.36–7.30 (m, 5H, ArH), 7.21 (t, *J* = 7.8 Hz, 1H, ArH), 7.12 (d, *J* = 7.2 Hz, 1H, ArH), 6.92 (t, *J* = 7.8 Hz, 1H, ArH), 6.78 (d, *J* = 7.8 Hz, 1H, ArH), 5.04 (s, 1H, CH), 5.01 (s, 2H, CH_2_); ^13^C NMR (150 MHz, CDCl_3_) δ 190.7, 170.4, 144.6, 135.2, 135.1, 134.5, 131.0, 129.0, 128.9, 128.3, 128.0, 127.3, 124.5, 123.3, 119.3, 110.0, 64.0, 61.0, 44.5; HRMS–ESI (*m*/*z*): [M + Na]^+^ calcd for C_23_H_17_NNaO_3_, 378.1101; found, 378.1103.

## Supporting Information

Single-crystal data for compounds **3c** (CCDC 919779), **4a** (CCDC 921900) and **5o** (CCDC 919780) have been deposited in the Cambridge Crystallographic Data Centre. These data can be obtained free of charge via http://www.ccdc.ac.ck./data_request/cif.

File 1 Experimental details and spectroscopic data.
