# Supplementary material for: Facile synthesis of functionalized spiro[indoline-3,2'-oxiran]-2-ones by Darzens reaction
Source: Beilstein J Org Chem. 2013 May 13;9:918–24. doi: 10.3762/bjoc.9.105 (PMC3678858; doi:10.3762/bjoc.9.105)

Supporting Information

for

Facile synthesis of functionalized spiro[indoline-3,2'-oxiran]-2-ones by Darzens reaction

Qin Fu and Chao-Guo Yan*

Address: College of Chemistry & Chemical Engineering, Yangzhou University, Yangzhou 225002, China

Email: Chao-Guo Yan - cgyan@yzu.edu.cn

* Corresponding author

Experimental details and spectroscopic data

1. General procedure for the preparation of spiro[indoline-3,2'-oxiran]-2-ones 3a–h: A mixture of isatin (1.0 mmol), phenacyl bromide (1.0 mmol) and potassium carbonate (1.2 mmol) in 20.0 mL chloroform was stirred at 50 °C for 10–24 h (TLC analysis). After cooling, the reaction was quenched with water. The solvent was evaporated under reduced pressure. The residue was recrystallized from ethanol to give the pure product for analysis.

3'-benzoylspiro[indoline-3,2'-oxiran]-2-one (3a): white solid, yield: 83%. mp 158–159 °C. IR (KBr) υ: 3457, 3180, 2972, 1735, 1676, 1620, 1597, 1469, 1335, 1219, 1041, 1013, 927, 850, 794 cm^{-1}; ^{1}H NMR (600 MHz, DMSO-d_6) δ *cis*-isomer: 11.04 (s, 1H, NH), 7.88 (d, *J* = 7.8 Hz, 2H, ArH), 7.66 (t, *J* = 7.2 Hz, 1H, ArH), 7.53 (t, *J* = 7.8 Hz, 2H, ArH), 7.28 (t, *J* = 7.2 Hz, 1H, ArH), 6.93 (d, *J* = 7.8 Hz, 1H, ArH), 6.88 (t, *J* = 7.2 Hz, 1H, ArH), 6.82 (d, *J* = 7.8 Hz, 1H, ArH), 5.15 (s, 1H, CH); *trans*-isomer: 7.96 (d, *J* = 7.8 Hz, 2H, ArH), 7.76 (t, *J* = 7.2 Hz, 1H, ArH), 7.62 (d, *J* = 7.2 Hz, 1H, ArH), 7.55 (t, *J* = 7.8 Hz, 2H, ArH), 7.32 (d, *J* = 7.8 Hz, 1H, ArH), 7.14 (d, *J* = 7.8 Hz, 1H, ArH), 6.97 (d, *J* = 7.2 Hz, 1H, ArH), 5.28 (s, 1H, CH); ^{13}C NMR (150 MHz, DMSO-d_6) δ 191.0, 170.8, 144.0, 134.7, 134.4, 131.1, 129.1, 128.0, 123.2, 122.0, 119.3, 110.0, 63.6, 60.4; HRMS–ESI (*m*/*z*): $[M + Na]^+$ calcd for $C_{16}H_{11}NNaO_3$: 288.0631; found: 288.0628.

3'-(4-chlorobenzoyl)spiro[indoline-3,2'-oxiran]-2-one (3b): white solid, yield: 72%. mp 162–164 °C. IR (KBr) υ: 3092, 2842, 1732, 1688, 1622, 1586, 1457, 1341, 1295, 1231, 1089, 1008, 917, 837, 767 cm^{-1}; ^{1}H NMR (600 MHz, DMSO-d_6) δ 7.90 (brs, 2H, ArH), 7.61 (brs, 2H, ArH), 7.29 (s, 1H, ArH), 6.92 (brs, 2H, ArH), 6.80 (s, 1H, ArH), 5.16 (s, 1H, CH); ^{13}C NMR (150 MHz, DMSO-d_6) δ 190.5, 170.4, 143.1, 139.8, 135.1, 133.3, 130.6, 130.1, 129.3, 126.0, 123.2, 121.3, 112.5, 63.4, 60.4; HRMS–ESI (*m*/*z*): $[M + Na]^+$ calcd for $C_{16}H_{10}ClNNaO_3$: 322.0241; found: 322.0237.

3'-benzoyl-5-methylspiro[indoline-3,2'-oxiran]-2-one (3c): white solid, yield: 85%. mp 170–172 °C. IR (KBr) υ: 3390, 1735, 1687, 1621, 1597, 1469, 1336, 1230, 1013, 927, 790 cm^{-1}; ^{1}H NMR (600 MHz, $CDCl_3$) δ 8.56 (s, 1H, NH), 7.95 (d, *J* = 7.8 Hz, 2H, ArH), 7.61 (t, *J* = 7.2 Hz, 1H, ArH), 7.48 (t, *J* = 7.2 Hz, 2H, ArH), 7.08 (d, *J* = 7.8 Hz, 1H, ArH), 6.92 (s, 1H, ArH), 6.84 (d, *J* = 7.8 Hz, 1H, ArH), 4.96 (s, 1H, CH), 2.21 (s, 3H, CH_3); ^{13}C NMR (150 MHz, $CDCl_3$) δ 190.7, 172.7, 142.7, 135.0, 134.6, 131.2, 129.1, 128.4, 124.7, 123.3, 119.5, 111.3, 63.8, 61.3, 21.0; HRMS–ESI (*m*/*z*): $[M + Na]^+$ calcd for $C_{17}H_{13}NNaO_3$: 302.0788; found: 302.0784.

3'-(4-chlorobenzoyl)-5-methylspiro[indoline-3,2'-oxiran]-2-one (3d): white solid, yield: 80%.

mp 166–167 °C. IR (KBr) υ: 3067, 1724, 1687, 1625, 1588, 1488, 1334, 1312, 1232, 1092, 1010, 934, 855, 796 cm^{-1}; ^{1}H NMR (600 MHz, DMSO-d_6) δ 10.97 (s, 1H, NH), 7.91 (d, J = 7.8 Hz, 2H, ArH), 7.61 (d, J = 7.8 Hz, 2H, ArH), 7.10 (d, J = 7.8 Hz, 1H, ArH), 6.83 (d, J = 7.8 Hz, 1H, ArH), 6.62 (s, 1H, ArH), 5.13 (s, 1H, CH), 2.13 (s, 3H, CH_3); ^{13}C NMR (150 MHz, DMSO-d_6) δ 190.2, 170.7, 141.6, 139.4, 133.4, 131.4, 131.0, 129.9, 129.3, 123.7, 119.3, 110.8, 63.5, 60.6, 20.5; HRMS–ESI (m/z): $[M + Na]^+$ calcd for $C_{17}H_{12}ClNNaO_3$: 336.0398; found: 336.0395.

3'-benzoyl-5-fluorospiro[indoline-3,2'-oxiran]-2-one (3e): white solid, yield: 78%. mp 205–206 °C. IR (KBr) υ: 3211, 3091, 1731, 1690, 1626, 1481, 1353, 1300, 1235, 1184, 1156, 1014, 931, 885, 776 cm^{-1}; ^{1}H NMR (600 MHz, DMSO-d_6) δ 11.11 (s, 1H, NH), 7.92 (d, J = 7.8 Hz, 2H, ArH), 7.69 (t, J = 7.8 Hz, 1H, ArH), 7.55 (t, J = 7.8 Hz, 2H, ArH), 7.17 (td, J_1 = 8.4 Hz, J_2 = 2.4 Hz, 1H, ArH), 6.94 (dd, J_1 = 8.4 Hz, J_2 = 4.2 Hz, 1H, ArH), 6.62 (dd, J_1 = 7.8 Hz, J_2 = 1.8 Hz, 1H, ArH), 5.22 (s, 1H, CH); ^{13}C NMR (150 MHz, DMSO-d_6) δ 190.9, 170.8, 157.6 (d, J = 237.0 Hz), 140.3, 134.7, 134.5, 129.1, 128.1, 121.0 (d, J = 8.7 Hz), 117.5 (d, J = 23.1 Hz), 112.0 (d, J = 7.8 Hz), 110.7 (d, J = 26.0 Hz), 63.5, 60.4; HRMS–ESI (m/z): $[M + Na]^+$ calcd for $C_{16}H_{10}FNNaO_3$: 306.0537; found: 306.0534.

3'-(4-chlorobenzoyl)-5-fluorospiro[indoline-3,2'-oxiran]-2-one (3f): white solid, yield: 77%. mp 220–221 °C. IR (KBr) υ: 3090, 1726, 1690, 1629, 1588, 1481, 1334, 1303, 1234, 1179, 1155, 1010, 936, 862, 743 cm^{-1}; ^{1}H NMR (600 MHz, DMSO-d_6) δ *cis*-isomer: 11.10 (s, 1H, NH), 7.95 (d, J = 8.4 Hz, 2H, ArH), 7.62 (d, J = 8.4 Hz, 2H, ArH), 7.17 (t, J = 8.4 Hz, 1H, ArH), 6.97–6.93 (m, 1H, ArH), 6.62 (d, J = 7.8 Hz, 1H, ArH), 5.21 (s, 1H, CH); *trans*-isomer: ^{1}H NMR (600 MHz, DMSO-d_6) δ 10.92 (s, 1H, NH), 7.86 (d, J = 8.4 Hz, 2H, ArH), 7.65 (d, J = 8.4 Hz, 2H, ArH), 7.29 (d, J = 7.8 Hz, 1H, ArH), 7.25 (d, J = 8.4 Hz, 1H, ArH), 5.28 (s, 1H, CH); ^{13}C NMR (150 MHz, DMSO-d_6) δ 190.0, 170.7, 158.7 (d, J = 236.3 Hz), 140.3, 139.6, 133.3, 130.0, 129.2, 120.9 (d, J = 8.2 Hz), 117.5 (d, J = 23.3 Hz), 112.0 (d, J = 6.2 Hz), 110.8 (d, J = 25.8 Hz), 63.4, 60.5; HRMS–ESI (m/z): $[M + Na]^+$ calcd for $C_{16}H_9ClFNNaO_3$: 340.0147; found: 340.0143.

3'-benzoyl-5-chlorospiro[indoline-3,2'-oxiran]-2-one (3g): white solid, yield: 86%. mp 200–202 °C. IR (KBr) υ: 3440, 3206, 1746, 1713, 1691, 1625, 1474, 1325, 1227, 1013, 826, 789 cm^{-1}; ^{1}H NMR (600 MHz, DMSO-d_6) δ 11.18 (s, 1H, NH), 7.92 (d, J = 7.8 Hz, 2H, ArH), 7.69 (t, J = 7.8 Hz, 1H, ArH), 7.54 (t, J = 7.8 Hz, 2H, ArH), 7.36 (dd, J_1 = 8.4 Hz, J_2 = 1.8 Hz, 1H, ArH), 6.95 (d, J = 8.4 Hz, 1H, ArH), 6.82 (s, 1H, ArH), 5.20 (s, 1H, CH); ^{13}C NMR (150 MHz,

DMSO-d_6) δ 1910, 170.5, 143.1, 134.7, 134.6, 130.9, 129.2, 128.1, 125.8, 123.0, 121.5, 112.5, 63.6, 60.2; HRMS–ESI (*m*/*z*): $[M + Na]^+$ calcd for $C_{16}H_{10}ClNNaO_3$: 322.0237; found: 322.0233.

5-chloro-3'-(4-chlorobenzoyl)spiro[indoline-3,2'-oxiran]-2-one (3h): white solid, yield: 82%. mp 204–205 °C. IR (KBr) υ: 3079, 1728, 1689, 1622, 1589, 1474, 1325, 1308, 1227, 1175, 1082, 1013, 886, 756 cm^{-1}; ^1H NMR (600 MHz, DMSO-d_6) δ 11.18 (s, 1H, NH), 7.95 (d, *J* = 7.8 Hz, 2H, ArH), 7.62 (d, *J* = 8.4 Hz, 2H, ArH), 7.37 (d, *J* = 8.4 Hz, 1H, ArH), 6.96 (d, *J* = 8.4 Hz, 1H, ArH), 6.82 (s, 1H, ArH), 5.19 (s, 1H, CH); ^{13}C NMR (150 MHz, DMSO-d_6) δ 190.0, 170.4, 143.0, 139.6, 133.3, 130.9, 130.0, 129.2, 125.9, 123.1, 121.4, 112.5, 63.4, 60.3; HRMS–ESI (*m*/*z*): $[M + Na]^+$ calcd for $C_{16}H_9Cl_2NNaO_3$: 355.9851; found: 355.9853.

2. General procedure for the preparation of spiro[indoline-3,2'-oxiran]-2-ones 4a–e: A mixture of isatin (1.0 mmol), phenacyl bromide (2.2 mmol) and potassium carbonate (2.6 mmol) in 20.0 mL of chloroform was stirred at 50 °C for 10–24 h (TLC analysis). After cooling, the reaction was quenched with water. The solvent was evaporated under reduced pressure. The residue was recrystallized from ethanol to give the pure product for analysis.

3'-benzoyl-1-(2-oxo-2-phenylethyl)spiro[indoline-3,2'-oxiran]-2-one (4a): white solid, yield: 93%. mp 188–189 °C. IR (KBr) υ: 3449, 2929, 1733, 1696, 1613, 1597, 1467, 1351, 1229, 1186, 1101, 930, 786 cm^{-1}; ^1H NMR (600 MHz, DMSO-d_6) δ 8.12 (d, *J* = 7.8 Hz, 2H, ArH), 7.91 (d, *J* = 7.2 Hz, 2H, ArH), 7.76 (t, *J* = 7.2 Hz, 1H, ArH), 7.69 (t, *J* = 7.2 Hz, 1H, ArH), 7.63 (t, *J* = 7.2 Hz, 2H, ArH), 7.55 (t, *J* = 7.8 Hz, 2H, ArH), 7.31 (t, *J* = 7.8 Hz, 1H, ArH), 7.14 (d, *J* = 7.8 Hz, 1H, ArH), 6.96 (t, *J* = 7.2 Hz, 1H, ArH), 6.90 (d, *J* = 7.2 Hz, 1H, ArH), 5.56–5.49 (m, 2H, CH_2), 5.28 (s, 1H, CH); ^{13}C NMR (150 MHz, DMSO-d_6) δ 192.5, 190.9, 169.9, 144.9, 134.6, 134.5, 134.2, 134.1, 131.1, 129.1, 128.9, 128.3, 128.0, 122.9, 122.8, 118.7, 110.3, 64.1, 60.2, 47.3; HRMS–ESI (*m*/*z*): $[M + K]^+$ calcd for $C_{24}H_{17}KNO_4$: 422.0789; found: 422.0782.

3'-(4-chlorobenzoyl)-1-(2-(4-chlorophenyl)-2-oxoethyl)spiro[indoline-3,2'-oxiran]-2-one (4b): white solid, yield: 89%. mp 172–173 °C. IR (KBr) υ: 3445, 1730, 1692, 1618, 1588, 1469, 1401, 1228, 1192, 1091, 962, 748 cm^{-1}; ^1H NMR (600 MHz, DMSO-d_6) δ 8.14 (brs, 2H, ArH), 7.94 (brs, 2H, ArH), 7.69 (brs, 2H, ArH), 7.63 (brs, 2H, ArH), 7.33 (brs, 1H, ArH), 7.15 (brs, 1H, ArH), 6.96 (brs, 1H, ArH), 6.91 (brs, 1H, ArH), 5.52 (s, 2H, CH_2), 5.29 (s, 1H, CH); ^{13}C NMR (150 MHz, DMSO-d_6) δ 191.7, 190.0, 169.8, 144.9, 139.6, 139.1, 133.3, 132.9, 131.2, 130.2, 130.0, 129.3, 129.0, 122.8, 118.6, 110.4, 110.3, 63.9, 60.2, 47.2; HRMS–ESI (*m*/*z*): $[M + K]^+$ calcd for

$C_{24}H_{15}Cl_2KNO_4$: 490.0010; found: 490.0008.

3'-benzoyl-5-methyl-1-(2-oxo-2-phenylethyl)spiro[indoline-3,2'-oxiran]-2-one (4c): white solid, yield: 90%. mp 198–199 °C. IR (KBr) υ: 3061, 2928, 1729, 1691, 1626, 1596, 1491, 1450, 1415, 1361, 1278, 1226, 810, 754 cm^{-1}; ^1H NMR (600 MHz, DMSO-d_6) δ 8.13 (d, J = 7.8 Hz, 2H, ArH), 7.93 (d, J = 7.8 Hz, 2H, ArH), 7.75 (t, J = 7.2 Hz, 1H, ArH), 7.69 (t, J = 7.2 Hz, 1H, ArH), 7.62 (t, J = 7.2 Hz, 2H, ArH), 7.56 (t, J = 7.2 Hz, 2H, ArH), 7.13 (d, J = 7.8 Hz, 1H, ArH), 7.04 (d, J = 7.8 Hz, 1H, ArH), 6.74 (s, 1H, ArH), 5.55–5.47 (m, 2H, CH_2), 5.27 (s, 1H, CH), 2.14 (s, 3H, CH_3); ^{13}C NMR (150 MHz, DMSO-d_6) δ 192.5, 190.9, 169.9, 142.6, 134.7, 134.5, 134.3, 134.2, 131.8, 131.3, 129.1, 128.9, 128.3, 128.0, 123.4, 118.8, 110.0, 64.1, 60.2, 47.3, 20.5; HRMS–ESI (*m*/*z*): $[M + K]^+$ calcd for $C_{25}H_{19}KNO_4$: 436.0946; found: 436.0942.

3'-(4-chlorobenzoyl)-1-(2-(4-chlorophenyl)-2-oxoethyl)-5-methylspiro[indoline-3,2'-oxiran]-2-one (4d): white solid, yield: 85%. mp 192–194 °C. IR (KBr) υ: 3441, 1740, 1695, 1590, 1490, 1368, 1230, 1192, 1093, 1010, 823 cm^{-1}; ^1H NMR (600 MHz, $CDCl_3$) δ 7.99 (d, J = 7.8 Hz, 2H, ArH), 7.90 (d, J = 7.8 Hz, 2H, ArH), 7.53 (d, J = 7.8 Hz, 2H, ArH), 7.47 (d, J = 7.8 Hz, 2H, ArH), 7.06 (d, J = 7.2 Hz, 1H, ArH), 6.89 (s, 1H, ArH), 6.59 (d, J = 7.8 Hz, 1H, ArH), 5.37 (d, J = 17.4 Hz, 1H, CH), 5.00 (d, J = 17.4 Hz, 1H, CH), 4.92 (s, 1H, CH), 2.20 (s, 3H, CH_3); ^{13}C NMR (150 MHz, DMSO-d_6) δ 191.7, 190.0, 169.8, 142.6, 139.5, 139.1, 133.4, 132.9, 131.9, 131.4, 130.2, 129.9, 129.3, 129.0, 123.4, 118.6, 110.1, 64.0, 60.2, 47.3, 20.5; HRMS–ESI (*m*/*z*): $[M + K]^+$ calcd for $C_{25}H_{17}Cl_2KNO_4$: 504.0170; found: 504.0173.

5-chloro-3'-(4-chlorobenzoyl)-1-(2-(4-chlorophenyl)-2-oxoethyl)spiro[indoline-3,2'-oxiran]-2-one (4e): white solid, yield: 88%. mp 180–181 °C. IR (KBr) υ: 3442, 2924, 1732, 1691, 1618, 1589, 1489, 1443, 1364, 1228, 1093, 964, 818 cm^{-1}; ^1H NMR (600 MHz, DMSO-d_6) δ 8.13 (d, J = 8.4 Hz, 2H, ArH), 7.98 (d, J = 8.4 Hz, 2H, ArH), 7.71 (d, J = 8.7 Hz, 2H, ArH), 7.65 (d, J = 8.4 Hz, 2H, ArH), 7.44 (dd, J_1 = 8.4 Hz, J_2 = 3.6 Hz, 1H, ArH), 7.22 (d, J = 8.4 Hz, 1H, ArH), 6.89 (d, J = 8.4 Hz, 1H, ArH), 5.56–5.49 (m, 2H, CH_2), 5.34 (s, 1H, CH); ^{13}C NMR (150 MHz, DMSO-d_6) δ 191.5, 189.9, 169.5, 143.9, 139.7, 139.2, 133.3, 132.8, 131.0, 130.3, 130.0, 129.3, 129.0, 126.7, 122.7, 120.7, 112.0, 63.9, 59.9, 47.3; HRMS–ESI (*m*/*z*): $[M + K]^+$ calcd for $C_{24}H_{14}Cl_3KNO_4$: 523.9620; found: 523.9623.

3. General procedure for the preparation of spiro[indoline-3,2'-oxiran]-2-ones 5a–p: A mixture of isatin (1.0 mmol), phenacyl bromide (1.2 mmol) and potassium carbonate (1.5 mmol)

in 20.0 mL of cholorform was stirred at 50 °C for 10–24 h (TLC analysis). After cooling, the reaction was quenched with water. The solvent was evaporated under reduced pressure. The residue was recrystallized from ethanol to give the pure product for analysis.

3'-benzoyl-1-benzylspiro[indoline-3,2'-oxiran]-2-one (5a): white solid, yield: 92%. mp 154–156 °C. IR (KBr) υ: 3034, 2923, 1730, 1691, 1610, 1463, 1360, 1232, 1185, 1104, 1007, 923, 870 cm^{-1}; ^1H NMR (600 MHz, $CDCl_3$) δ 7.94 (d, *J* = 7.8 Hz, 2H, ArH), 7.61 (t, *J* = 7.2 Hz, 1H, ArH), 7.47 (t, *J* = 7.8 Hz, 2H, ArH), 7.36–7.30 (m, 5H, ArH), 7.21 (t, *J* = 7.8 Hz, 1H, ArH), 7.12 (d, *J* = 7.2 Hz, 1H, ArH), 6.92 (t, *J* = 7.8 Hz, 1H, ArH), 6.78 (d, *J* = 7.8 Hz, 1H, ArH), 5.04 (s, 1H, CH), 5.01 (s, 2H, CH_2); ^{13}C NMR (150 MHz, $CDCl_3$) δ 190.7, 170.4, 144.6, 135.2, 135.1, 134.5, 131.0, 129.0, 128.9, 128.3, 128.0, 127.3, 124.5, 123.3, 119.3, 110.0, 64.0, 61.0, 44.5; HRMS–ESI (*m*/*z*): $[M + Na]^+$ calcd for $C_{23}H_{17}NNaO_3$: 378.1101; found: 378.1103.

1-benzyl-3'-(4-chlorobenzoyl)spiro[indoline-3,2'-oxiran]-2-one (5b): white solid, yield: 80%. mp 100–102 °C. IR (KBr) υ: 3065, 2923, 1727, 1688, 1590, 1468, 1358, 1233, 1182, 1009, 927, 857 cm^{-1}; ^1H NMR (600 MHz, $CDCl_3$) δ *cis*-isomer: 8.03 (d, *J* = 8.4 Hz, 2H, ArH), 7.46 (d, *J* = 8.4 Hz, 2H, ArH), 7.32 (t, *J* = 7.8 Hz, 2H, ArH), 7.28 (s, 1H, ArH), 7.21 (t, *J* = 7.2 Hz, 4H, ArH), 7.11 (t, *J* = 7.2 Hz, 1H, ArH), 6.83 (d, *J* = 8.4 Hz, 1H, ArH), 4.88 (d, *J* = 15.6 Hz, 1H, CH), 4.82 (d, *J* = 15.6 Hz, 1H, CH), 4.58 (s, 1H, CH); *trans*-isomer: 7.90 (d, *J* = 8.4 Hz, 2H, ArH), 7.52 (d, *J* = 8.4 Hz, 2H, ArH), 7.06 (d, *J* = 7.2 Hz, 2H, ArH), 6.93 (t, *J* = 7.8 Hz, 2H, ArH), 6.80 (d, *J* = 7.8 Hz, 1H, ArH), 5.01 (s, 2H, CH_2), 4.99 (s, 1H, CH); ^{13}C NMR (150 MHz, $CDCl_3$) δ 190.1, 189.7, 170.3, 169.2, 144.6, 144.5, 141.2, 140.5, 135.1, 135.0, 133.8, 133.4, 131.3, 131.1, 130.2, 129.8, 129.4, 129.1, 129.0, 128.9, 128.0, 127.9, 127.4, 127.3, 124.3, 124.2, 123.3, 122.5, 110.2, 110.1, 64.9, 63.8, 61.6, 61.0, 44.5, 44.3; HRMS–ESI (*m*/*z*): $[M + Na]^+$ calcd for $C_{23}H_{16}ClNNaO_3$: 412.0711; found: 412.0713.

3'-benzoyl-1-butylspiro[indoline-3,2'-oxiran]-2-one (5c): white solid, yield: 88%. mp 116–118 °C. IR (KBr) υ: 2964, 2930, 2864, 1720, 1693, 1615, 1469, 1366, 1228, 1194, 1108, 1008, 932, 827 cm^{-1}; ^1H NMR (600 MHz, $CDCl_3$) δ 7.93 (d, *J* = 7.8 Hz, 2H, ArH), 7.60 (t, *J* = 7.2 Hz, 1H, ArH), 7.46 (t, *J* = 7.8 Hz, 2H, ArH), 7.32 (t, *J* = 7.8 Hz, 1H, ArH), 7.12 (d, *J* = 7.2 Hz, 1H, ArH), 6.94 (t, *J* = 7.2 Hz, 1H, ArH), 6.90 (d, *J* = 7.8 Hz, 1H, ArH), 4.98 (s, 1H, CH), 3.80 (t, *J* = 7.2 Hz, 2H, CH_2), 1.75–1.70 (m, 2H, CH_2), 1.47–1.41 (m, 2H, CH_2), 0.99 (t, *J* = 7.2 Hz, 3H, CH_3); ^{13}C NMR (150 MHz, $CDCl_3$) δ 190.8, 170.1, 144.9, 135.1, 134.4, 131.0, 129.0, 128.3,

124.5, 123.0, 119.4, 109.3, 63.8, 60.9, 40.5, 29.4, 20.2, 13.7; HRMS–ESI (*m*/*z*): $[M + Na]^+$ calcd for $C_{20}H_{19}NNaO_3$: 344.1257; found: 344.1257.

1-butyl-3'-(4-chlorobenzoyl)spiro[indoline-3,2'-oxiran]-2-one (5d): white solid, 76%. mp 110–112 °C. IR (KBr) υ: 2931, 2868, 1729, 1687, 1622, 1464, 1369, 1234, 1196, 1137, 1088, 1005, 929, 832 cm^{-1}; ^1H NMR (600 MHz, $CDCl_3$) δ *cis*-isomer: 7.89 (d, *J* = 8.4 Hz, 2H, ArH), 7.46–7.42 (m, 3H, ArH), 7.33 (t, *J* = 7.8 Hz, 1H, ArH), 6.94 (t, *J* = 7.8 Hz, 1H, ArH), 6.91 (d, *J* = 7.8 Hz, 1H, ArH), 4.92 (s, 1H, CH), 3.80 (t, *J* = 7.8 Hz, 2H, CH_2), 1.74–1.69 (m, 2H, CH_2), 1.46–1.40 (m, 2H, CH_2), 0.99 (t, *J* = 7.8 Hz, 3H, CH_3); *trans*-isomer: 8.04 (d, *J* = 8.4 Hz, 2H, ArH), 7.21 (d, *J* = 7.2 Hz, 2H, ArH), 7.13 (t, *J* = 7.8 Hz, 2H, ArH), 4.53 (s, 2H, CH_2), 3.68–3.62 (m, 2H, CH_2), 1.61–1.58 (m, 2H, CH_2), 1.33–1.28 (m, 2H, CH_2), 0.89 (t, *J* = 7.2 Hz, 3H, CH_3); ^{13}C NMR (150 MHz, $CDCl_3$) δ 190.4, 189.8, 169.9, 169.0, 144.9, 141.1, 140.4, 133.9, 133.4, 131.3, 131.1, 130.2, 129.8, 129.4, 129.0, 124.4, 123.0, 122.6, 121.2, 119.2, 109.5, 109.3, 64.7, 63.6, 61.6, 60.9, 40.6, 40.4, 29.4, 29.3, 202, 20.1, 13.7, 13.6; MS(ESI+): (*m*/*z*) 513.48; HRMS–ESI (*m*/*z*): $[M + Na]^+$ calcd for $C_{20}H_{18}ClNNaO_3$: 378.0867; found: 378.087.

3'-benzoyl-1-benzyl-5-methylspiro[indoline-3,2'-oxiran]-2-one (5e): white solid, 90%. mp 140–142 °C. IR (KBr) υ: 3069, 2921, 1730, 1684, 1626, 1601, 1595, 1450, 1363, 1236, 1185, 1032, 934, 818 cm^{-1}; ^1H NMR (600 MHz, $CDCl_3$) δ *cis*-isomer: 7.94 (d, *J* = 7.2 Hz, 2H, ArH), 7.47 (t, *J* = 7.8 Hz, 3H, ArH), 7.35–7.32 (m, 4H, ArH), 7.18 (d, *J* = 6.6 Hz, 1H, ArH), 7.01 (d, *J* = 7.8 Hz, 1H, ArH), 6.93 (s, 1H, ArH), 6.66 (d, *J* = 7.8 Hz, 1H, CH), 5.03 (s, 1H, CH), 4.99 (s, 2H, CH_2), 2.18 (s, 3H, CH_3); *trans*-isomer: 8.04 (d, *J* = 7.2 Hz, 2H, ArH), 7.61 (t, *J* = 7.2 Hz, 2H, ArH), 4.87 (d, *J* = 15.6 Hz, 1H, CH), 4.77 (d, *J* = 15.6 Hz, 1H, CH), 4.61 (s, 1H, CH), 2.33 (s, 3H, CH_3); ^{13}C NMR (150 MHz, $CDCl_3$) δ 190.8, 190.7, 170.4, 169.2, 142.1, 135.3, 135.2, 134.4, 133.9, 133.0, 132.9, 131.5, 131.3, 129.0, 128.9, 128.8, 128.7, 128.3, 127.9, 127.8, 127.4, 127.3, 125.1, 123.2, 121.3, 119.3, 110.0, 109.8, 65.1, 64.1, 61.5, 58.4, 44.5, 44.2, 21.0, 20.9; HRMS–ESI (*m*/*z*): $[M + Na]^+$ calcd for $C_{24}H_{19}NNaO_3$: 392.1257; found: 392.1262.

1-benzyl-3'-(4-chlorobenzoyl)-5-methylspiro[indoline-3,2'-oxiran]-2-one (5f): white solid, 86%. mp 102–104 °C. IR (KBr) υ: 3063, 2923, 1728, 1689, 1624, 1592, 1491, 1445, 1400, 1358, 1229, 1180, 1090, 927, 818 cm^{-1}; ^1H NMR (600 MHz, $CDCl_3$) δ *cis*-isomer: 7.89 (d, *J* = 8.4 Hz, 2H, ArH), 7.45 (d, *J* = 8.4 Hz, 2H, ArH), 7.36–7.28 (m, 5H, ArH), 7.02 (d, *J* = 7.8 Hz, 1H, ArH), 6.87 (s, 1H, ArH), 6.68 (d, *J* = 7.8 Hz, 1H, ArH), 4.99 (s, 2H, CH_2), 4.97 (s, 1H, CH), 2.18 (s, 3H,

CH_3); *trans*-isomer: 20:7; 8.02 (d, *J* = 7.8 Hz, 2H, ArH), 6.71 (d, *J* = 7.8 Hz, 2H, ArH), 4.85 (d, *J* = 15.6 Hz, 1H, CH), 4.79 (d, *J* = 15.6 Hz, 1H, CH), 4.55 (s, 1H, CH), 2.33 (s, 3H, CH_3); ^{13}C NMR (150 MHz, $CDCl_3$) δ 190.1, 189.7, 1702, 169.2, 142.2, 142.1, 141.1, 140.4, 135.2, 135.1, 133.8, 133.5, 133.1, 133.0, 131.6, 131.4, 130.2, 129.7, 129.4, 129.1, 128.9, 128.8, 128.0, 127.9, 127.4, 127.3, 125.0, 123.2, 119.1, 110.0, 109.9, 64.9, 63.9, 61.7, 44.5, 44.3, 20.9, 18.4; HRMS–ESI (*m*/*z*): $[M + Na]^+$ calcd for $C_{24}H_{18}ClNNaO_3$: 426.0867; found: 426.0869.

3'-benzoyl-1-butyl-5-methylspiro[indoline-3,2'-oxiran]-2-one (5g): white solid, 82%. mp 118–120 °C. IR (KBr) υ: 2958, 2930, 2868, 1727, 1689, 1623, 1597, 1492, 1453, 1357, 1229, 1191, 1129, 1005, 943, 817 cm^{-1}; ^1H NMR (600 MHz, $CDCl_3$) δ *cis*-isomer: 7.93 (d, *J* = 7.8 Hz, 2H, ArH), 7.60 (t, *J* = 7.2 Hz, 1H, ArH), 7.47 (t, *J* = 7.2 Hz, 2H, ArH), 7.11 (d, *J* = 7.2 Hz, 1H, ArH), 6.93 (s, 1H, ArH), 6.79 (d, *J* = 7.8 Hz, 1H, ArH), 4.97 (s, 1H, CH), 3.78 (t, *J* = 7.2 Hz, 2H, CH_2), 2.11 (s, 3H, CH_3), 1.73–1.68 (m, 2H, CH_2), 1.44–1.41 (m, 2H, CH_2), 0.98 (t, *J* = 7.2 Hz, 3H, CH_3); *trans*-isomer: 8.02 (d, *J* = 7.2 Hz, 2H, ArH), 7.58 (t, *J* = 7.2 Hz, 1H, ArH), 7.22 (d, *J* = 7.2 Hz, 1H, ArH), 6.83 (d, *J* = 7.8 Hz, 1H, ArH), 3.63 (t, *J* = 7.2 Hz, 2H, CH_2), 1.60–1.55 (m, 2H, CH_2), 1.28–1.26 (m, 2H, CH_2), 0.86 (t, *J* = 7.2 Hz, 3H, CH_3); ^{13}C NMR (150 MHz, $CDCl_3$) δ 190.9, 170.0, 142.5, 135.2, 134.4, 132.7, 131.3, 129.0, 128.7, 128.3, 125.1, 119.4, 109.0, 63.9, 61.0, 40.5, 29.4, 20.9, 20.1, 13.7; HRMS–ESI (*m*/*z*): $[M + Na]^+$ calcd for $C_{21}H_{21}NNaO_3$: 358.1414; found: 358.1433.

1-butyl-3'-(4-chlorobenzoyl)-5-methylspiro[indoline-3,2'-oxiran]-2-one (5h): white solid, 86%. mp 122–124 °C. IR (KBr) υ: 3068, 2957, 2865, 1737, 1688, 1625, 1592, 1457, 1357, 1236, 1200, 1089, 906, 842 cm^{-1}; ^1H NMR (600 MHz, $CDCl_3$) δ *cis*-isomer: 7.88 (d, *J* = 8.4 Hz, 2H, ArH), 7.44 (d, *J* = 8.4 Hz, 2H, ArH), 7.12 (d, *J* = 7.8 Hz, 1H, ArH), 6.87 (s, 1H, ArH), 6.79 (d, *J* = 7.8 Hz, 1H, ArH), 4.91 (s, 1H, CH), 3.78 (t, *J* = 7.2 Hz, 2H, CH_2), 2.21 (s, 3H, CH_3), 1.73–1.68 (m, 2H, CH_2), 1.45–1.40 (m, 2H, CH_2), 0.98 (t, *J* = 7.2 Hz, 3H, CH_3); *trans*-isomer: 8.02 (d, *J* = 7.2 Hz, 2H, ArH), 7.22 (d, *J* = 7.2 Hz, 1H, ArH), 6.83 (d, *J* = 7.8 Hz, 1H, ArH), 3.65–3.60 (m, 2H, CH_2), 2.37 (s, 3H, CH_3), 1.58–1.56 (m, 2H, CH_2), 1.31–1.25 (m, 2H, CH_2), 0.87 (t, *J* = 7.2 Hz, 3H, CH_3); ^{13}C NMR (150 MHz, $CDCl_3$) δ 190.4, 189.8, 169.8, 168.9, 142.5, 141.0, 140.3, 133.9, 133.5, 132.7, 131.5, 131.4, 130.2, 129.7, 129.4, 129.0, 125.0, 123.3, 121.2, 119.2, 109.2, 109.1, 64.7, 63.7, 61.6, 40.3, 29.4, 29.3, 20.9, 20.2, 20.0, 13.7, 13.6; HRMS–ESI (*m*/*z*): $[M + Na]^+$ calcd for $C_{21}H_{20}ClNNaO_3$: 392.1024; found: 392.1025.

3'-benzoyl-1-benzyl-5-fluorospiro[indoline-3,2'-oxiran]-2-one (5i): white solid, 80%. mp 106–108 °C. IR (KBr): IR (KBr) υ: 3064, 1732, 1687, 1622, 1465, 1345, 1271, 1180, 1080, 1030, 929, 820 cm^{-1}; ^1H NMR (600 MHz, $CDCl_3$) δ *cis*-isomer: 7.69 (d, *J* = 7.8 Hz, 2H, ArH), 7.63 (t, *J* = 7.2 Hz, 2H, ArH), 7.49 (t, *J* = 7.2 Hz, 1H, ArH), 7.37–7.28 (m, 4H, ArH), 7.18 (d, *J* = 7.2 Hz, 1H, ArH), 7.02 (td, J_1 = 8.4 Hz, J_2 = 2.4 Hz, 1H, ArH), 6.93-6.90 (m, 1H, ArH), 6.69 (dd, J_1 = 7.8 Hz, J_2 = 3.6 Hz, 1H, ArH), 5.06 (s, 1H, CH), 5.00 (s, 2H, CH_2); *trans*-isomer: 8.05 (d, *J* = 7.2 Hz, 2H, ArH), 6.98 (dd, J_1 = 7.2 Hz, J_2 = 2.4 Hz, 1H, ArH), 6.73 (dd, J_1 = 7.8 Hz, J_2 = 3.6 Hz, 1H, ArH), 4.89 (d, *J* = 15.6 Hz, 1H, CH), 4.80 (d, *J* = 15.6 Hz, 1H, CH), 4.60 (s, 1H, CH); ^{13}C NMR (150 MHz, $CDCl_3$) δ 190.4, 190.3, 170.2, 169.0, 159.4 (d, *J* = 241.4 Hz), 159.1 (d, *J* = 241.4 Hz), 140.4, 140.3, 135.1, 135.0, 134.7, 134.1, 129.1, 129.0, 128.9, 128.8, 128.7, 128.4, 128.1, 128.0, 127.4, 127.3, 122.9 (d, *J* = 8.9 Hz), 120.9 (d, *J* = 8.9 Hz), 117.7, 117.5, 117.4, 112.9, 112.7, 111.1 (d, *J* = 7.8 Hz), 110.7 (d, *J* = 7.8 Hz), 110.6, 65.1, 63.9, 61.3, 60.8, 44.6, 44.4; HRMS–ESI (*m*/*z*): $[M + Na]^+$ calcd for $C_{23}H_{16}FNNaO_3$: 396.1006; found: 396.1009.

1-benzyl-3'-(4-chlorobenzoyl)-5-fluorospiro[indoline-3,2'-oxiran]-2-one (5j): white solid, 89%. mp 150–152 °C. IR (KBr) υ: 3061, 1726, 1694, 1621, 1589, 1466, 1401, 1338, 1269, 1177, 1090, 958, 819 cm^{-1}; ^1H NMR (600 MHz, $CDCl_3$) δ *cis*-isomer: 7.91 (d, *J* = 8.4 Hz, 2H, ArH), 7.47 (d, *J* = 8.4 Hz, 2H, ArH), 7.37–7.28 (m, 5H, ArH), 6.92 (td, J_1 = 8.4 Hz, J_2 = 2.4 Hz, 1H, ArH), 6.88 (dd, J_1 = 7.8 Hz, J_2 = 2.4 Hz, 1H, ArH), 6.70 (dd, J_1 = 8.4 Hz, J_2 = 3.6 Hz, 1H, ArH), 5.02–4.96 (m, 3H, CH_2, CH); *trans*-isomer: 8.04 (d, *J* = 8.4 Hz, 2H, ArH), 7.19 (d, *J* = 6.6 Hz, 2H, ArH), 7.02 (td, J_1 = 8.4 Hz, J_2 = 2.4 Hz, 1H, ArH), 6.96 (dd, J_1 = 7.2 Hz, J_2 = 2.4 Hz, 1H, ArH), 6.73 (dd, J_1 = 8.4 Hz, J_2 = 3.6 Hz, 1H, ArH), 4.86 (d, *J* = 15.6 Hz, 1H, CH), 4.82 (d, *J* = 15.6 Hz, 1H, CH), 4.55 (s, 1H, CH); ^{13}C NMR (150 MHz, $CDCl_3$) δ 189.3, 170.0, 159.1 (d, *J* = 241.7 Hz), 141.4, 140.5, 134.7, 133.3, 130.2, 129.8, 129.5, 129.2, 129.1, 128.9, 128.2, 128.1, 127.4, 127.3, 120.7 (d, *J* = 8.7 Hz), 117.5 (d, *J* = 23.4 Hz), 112.7 (d, *J* = 26.3 Hz), 110.8 (d, *J* = 7.8 Hz), 64.9, 63.6, 60.8, 44.7, 44.4; HRMS–ESI (*m*/*z*): $[M + Na]^+$ calcd for $C_{23}H_{15}ClFNNaO_3$: 430.0617; found: 430.0619.

3'-benzoyl-1-butyl-5-fluorospiro[indoline-3,2'-oxiran]-2-one (5k): white solid, 83%. mp 108–110 °C. IR (KBr): 3068, 2966, 2864, 1729, 1690, 1599, 1464, 1348, 1232, 1190, 1137, 950, 818 cm^{-1}; ^1H NMR (600 MHz, $CDCl_3$) δ *cis*-isomer: 7.94 (d, *J* = 7.8 Hz, 2H, ArH), 7.62 (t, *J* = 7.8 Hz, 1H, ArH), 7.48 (t, *J* = 7.8 Hz, 2H, ArH), 7.02 (td, J_1 = 8.4 Hz, J_2 = 3.6 Hz, 1H, ArH), 6.94 (d, *J* = 7.8 Hz, 1H, ArH), 6.82 (dd, J_1 = 8.4 Hz, J_2 = 3.6 Hz, 1H, ArH), 5.00 (s, 1H, CH), 3.79 (t, *J*

= 7.2 Hz, 2H, CH_2), 1.73–1.68 (m, 2H, CH_2), 1.46–1.40 (m, 2H, CH_2), 0.99 (t, J = 7.2 Hz, 3H, CH_3); *trans*-isomer: 100:9; 8.03 (d, J = 7.8 Hz, 2H, ArH), 7.14 (td, J_1 = 8.4 Hz, J_2 = 3.6 Hz, 1H, ArH), 6.98 (d, J = 7.2 Hz, 1H, ArH), 6.87 (dd, J_1 = 8.4 Hz, J_2 = 3.6 Hz, 1H, ArH), 4.55 (s, 1H, CH), 3.64 (t, J = 7.2 Hz, 2H, CH_2), 1.60–1.55 (m, 2H, CH_2), 1.29–1.24 (m, 2H, CH_2), 0.87 (t, J = 7.2 Hz, 3H, CH_3); ^{13}C NMR (150 MHz, $CDCl_3$) δ 190.4, 169.8, 158.9 (d, J = 240.9 Hz), 140.8, 135.0, 134.6, 129.1, 128.7, 128.6, 128.3, 121.0 (d, J = 8.9 Hz), 117.4 (d, J = 23.7 Hz), 112.7 (d, J = 23.6 Hz), 109.9 (d, J = 8.0 Hz), 63.7, 60.7, 40.6, 29.3, 20.1, 13.7; HRMS–ESI (*m*/*z*): $[M + Na]^+$ calcd for $C_{20}H_{18}FNNaO_3$: 362.1163; found: 362.1182.

1-butyl-3'-(4-chlorobenzoyl)-5-fluorospiro[indoline-3,2'-oxiran]-2-one (5l): white solid, 92%. mp 132–134 °C. IR (KBr): 2961, 2865, 1739, 1689, 1591, 1351, 1176, 1135, 1092, 950, 816 cm^{-1}; ^{1}H NMR (600 MHz, $CDCl_3$) δ *cis*-isomer: 7.90 (d, J = 8.4 Hz, 2H, ArH), 7.46 (d, J = 8.4 Hz, 2H, ArH), 7.04 (td, J_1 = 8.4 Hz, J_2 = 2.4 Hz, 1H, ArH), 6.88 (dd, J_1 = 7.8 Hz, J_2 = 2.4 Hz, 1H, ArH), 6.86–6.82 (m, 1H, ArH), 4.94 (s, 1H, CH), 3.78 (t, J = 7.8 Hz, 2H, CH_2), 1.72–1.68 (m, 2H, CH_2), 1.45–1.39 (m, 2H, CH_2), 0.99 (t, J = 7.2 Hz, 3H, CH_3); *trans*-isomer: 8.04 (d, J = 8.4 Hz, 2H, ArH), 7.13 (td, J_1 = 8.4 Hz, J_2 = 2.4 Hz, 1H, ArH), 6.96 (dd, J_1 = 7.8 Hz, J_2 = 2.4 Hz, 1H, ArH), 6.87–6.85 (m, 1H, ArH), 4.50 (s, 1H, CH), 3.67–3.61 (m, 2H, CH_2), 1.61–1.56 (m, 2H, CH_2), 1.32–1.26 (m, 2H, CH_2), 0.89 (t, J = 7.2 Hz, 3H, CH_3); ^{13}C NMR (150 MHz, $CDCl_3$) δ 189.4, 169.6, 158.9 (d, J = 241.4 Hz), 141.3, 140.8, 133.3, 130.2, 129.7, 129.6, 129.4, 129.0, 120.8 (d, J = 8.1 Hz), 117.5 (d, J = 24.2 Hz), 112.7 (d, J = 26.4 Hz), 110.0 (d, J = 6.9 Hz), 64.7, 63.5, 60.7, 40.7, 40.5, 29.3, 20.1, 13.7; HRMS–ESI (*m*/*z*): $[M + Na]^+$ calcd for $C_{20}H_{17}ClFNNaO_3$: 396.0773; found: 396.0776.

3'-benzoyl-1-benzyl-5-chlorospiro[indoline-3,2'-oxiran]-2-one (5m): white solid, 84%. mp 128–130 °C. IR (KBr): 3066, 3033, 1728, 1690, 1616, 1443, 1338, 1229, 1183, 1080, 1007, 946, 819 cm^{-1}; ^{1}H NMR (600 MHz, $CDCl_3$) δ *cis*-isomer: 7.96 (d, J = 7.2 Hz, 2H, ArH), 7.64 (t, J = 7.2 Hz, 1H, ArH), 7.50 (t, J = 7.8 Hz, 2H, ArH), 7.37–7.30 (m, 5H, ArH), 7.19 (d, J = 8.4 Hz, 1H, ArH), 7.14 (s, 1H, ArH), 6.69 (d, J = 8.4 Hz, 1H, ArH), 5.06 (s, 1H, CH), 5.00 (s, 2H, CH_2); *trans*-isomer: 8.04 (d, J = 7.2 Hz, 2H, ArH), 7.21 (s, 1H, ArH), 6.73 (d, J = 8.4 Hz, 1H, ArH), 4.89 (d, J = 15.6 Hz, 1H, CH), 4.80 (d, J = 15.6 Hz, 1H, CH), 4.61 (s, 1H, CH); ^{13}C NMR (150 MHz, $CDCl_3$) δ 190.3, 190.2, 170.0, 169.9, 143.0, 142.9, 135.0, 134.7, 134.6, 134.2, 131.1, 130.9, 129.1, 129.0, 128.9, 128.8, 128.7, 128.4, 128.2, 128.0, 127.4, 127.3, 125.0, 123.0, 121.0, 111.2, 111.0,

65.1, 63.9, 61.0, 60.6, 44.6, 44.4; HRMS–ESI (*m/z*): $[M + Na]^+$ calcd for $C_{23}H_{16}ClNNaO_3$: 412.0711; found: 412.0711.

1-benzyl-5-chloro-3'-(4-chlorobenzoyl)spiro[indoline-3,2'-oxiran]-2-one (5n): white solid, 91%. mp 174–176 °C. IR (KBr): 3069, 3015, 1732, 1688, 1590, 1439, 1400, 1338, 1175, 1120, 1088, 947, 822 cm^{-1}; ^1H NMR (600 MHz, $CDCl_3$) δ 7.92 (d, *J* = 8.4 Hz, 2H, ArH), 7.47 (d, *J* = 8.4 Hz, 2H, ArH), 7.36 (t, *J* = 6.6 Hz, 2H, ArH), 7.31 (t, *J* = 7.8 Hz, 3H, ArH), 7.20 (d, *J* = 6.6 Hz, 1H, ArH), 7.09 (s, 1H, ArH), 6.71 (d, *J* = 8.4 Hz, 1H, ArH), 5.02–4.95 (m, 3H, CH_2, CH); ^{13}C NMR (150 MHz, $CDCl_3$) δ 189.2, 169.8, 143.0, 141.4, 134.5, 133.3, 131.0, 130.2, 129.8, 129.5, 129.2, 129.1, 129.0, 128.2, 127.4, 127.3, 124.9, 120.8, 111.1, 63.6, 60.6, 44.6; HRMS–ESI (*m/z*): $[M + Na]^+$ calcd for $C_{23}H_{15}Cl_2NNaO_3$: 446.0321; found: 446.0320.

3'-benzoyl-1-butyl-5-chlorospiro[indoline-3,2'-oxiran]-2-one (3o): white solid, 81%. mp 146–148 °C. IR (KBr): 3068, 2962, 2867, 1732, 1690, 1609, 1446, 1350, 1229, 1186, 1130, 1088, 940, 870 cm^{-1}; ^1H NMR (600 MHz, $CDCl_3$) δ 7.95 (d, *J* = 7.2 Hz, 2H, ArH), 7.63 (t, *J* = 7.2 Hz, 1H, ArH), 7.48 (t, *J* = 7.2 Hz, 2H, ArH), 7.29 (d, *J* = 8.4 Hz, 1H, ArH), 7.14 (s, 1H, ArH), 6.83 (d, *J* = 8.4 Hz, 1H, ArH), 4.99 (s, 1H, CH), 3.79 (t, *J* = 7.2 Hz, 2H, CH_2), 1.72–1.67 (m, 2H, CH_2), 1.44–1.40 (m, 2H, CH_2), 0.98 (t, *J* = 7.2 Hz, 3H, CH_3); ^{13}C NMR (150 MHz, $CDCl_3$) δ 190.4, 169.6, 143.4, 135.0, 134.6, 130.9, 129.1, 128.5, 128.4, 121.1, 110.2, 63.7, 60.5, 40.7, 29.3, 20.1, 13.7; HRMS–ESI (*m/z*): $[M + Na]^+$ calcd for $C_{20}H_{18}ClNNaO_3$: 378.0867; found: 378.0870.

1-butyl-5-chloro-3'-(4-chlorobenzoyl)spiro[indoline-3,2'-oxiran]-2-one (5p): white solid, 88%. mp 126–128 °C. IR (KBr): 3094, 2962, 2863, 1744, 1689, 1613, 1589, 1440, 1348, 1233, 1198, 1133, 1089, 904, 840 cm^{-1}; ^1H NMR (600 MHz, $CDCl_3$) δ *cis*-isomer: 7.90 (d, *J* = 8.4 Hz, 2H, ArH), 7.47 (d, *J* = 8.4 Hz, 2H, ArH), 7.31 (d, *J* = 7.2 Hz, 1H, ArH), 7.09 (s, 1H, ArH), 6.83 (d, *J* = 8.4 Hz, 1H, ArH), 4.94 (s, 1H, CH), 3.78 (t, *J* = 7.2 Hz, 2H, CH_2), 1.72–1.67 (m, 2H, CH_2), 1.43–1.40 (m, 2H, CH_2), 0.98 (t, *J* = 7.2 Hz, 3H, CH_3); *trans*-isomer: 10:1; 8.03 (d, *J* = 8.4 Hz, 2H, ArH), 7.40 (d, *J* = 7.2 Hz, 1H, ArH), 7.18 (s, 1H, ArH), 6.87 (d, *J* = 7.8 Hz, 1H, ArH), 4.51 (s, 1H, CH), 3.86–3.62 (m, 2H, CH_2), 1.30–1.26 (m, 2H, CH_2), 0.88 (t, *J* = 7.2 Hz, 3H, CH_3); ^{13}C NMR (150 MHz, $CDCl_3$) δ 189.9, 189.3, 169.5, 168.5, 143.4, 141.3, 140.5, 133.7, 133.3, 131.1, 131.0, 130.4, 130.2, 129.8, 129.5, 129.1, 128.6, 128.5, 124.9, 123.1, 123.0, 120.9, 110.5, 110.3, 64.7, 63.5, 61.1, 60.5, 40.7, 40.5, 29.3, 29.2, 20.1, 20.0, 13.7, 13.6; HRMS–ESI (*m/z*): $[M + Na]^+$ calcd for $C_{20}H_{17}Cl_2NNaO_3$: 412.0478; found: 412.0478.

3'-benzoylspiro[indoline-3,2'-oxiran]-2-one (3a)

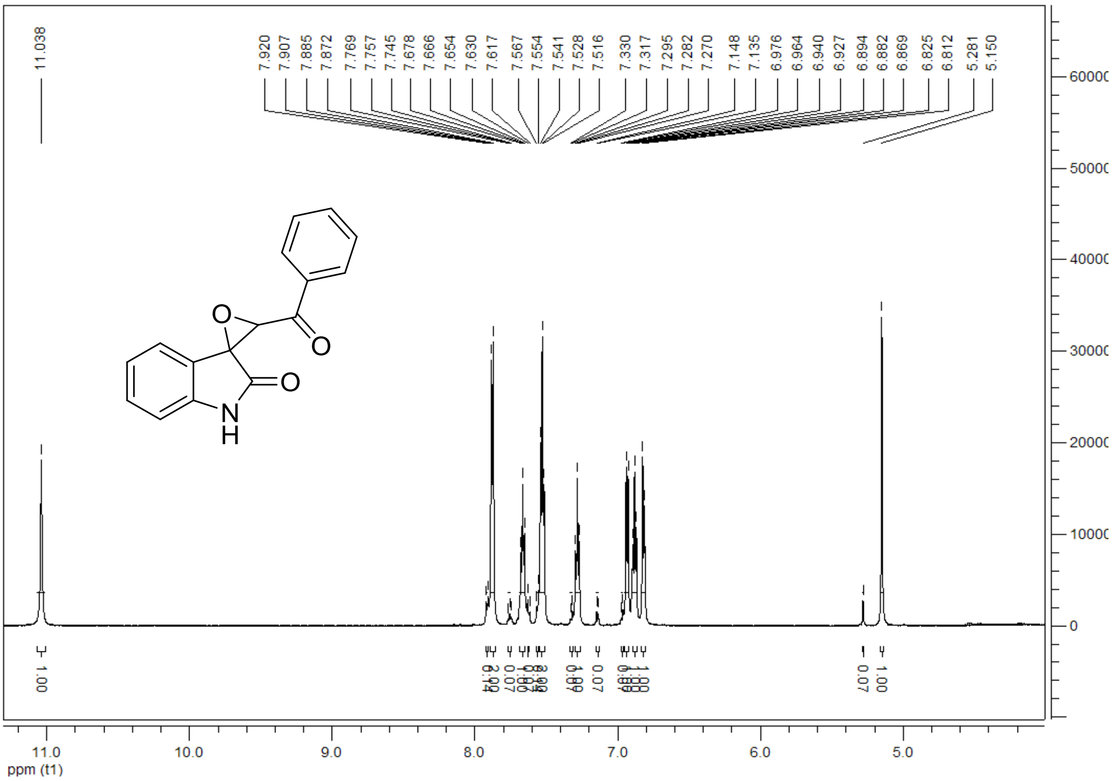

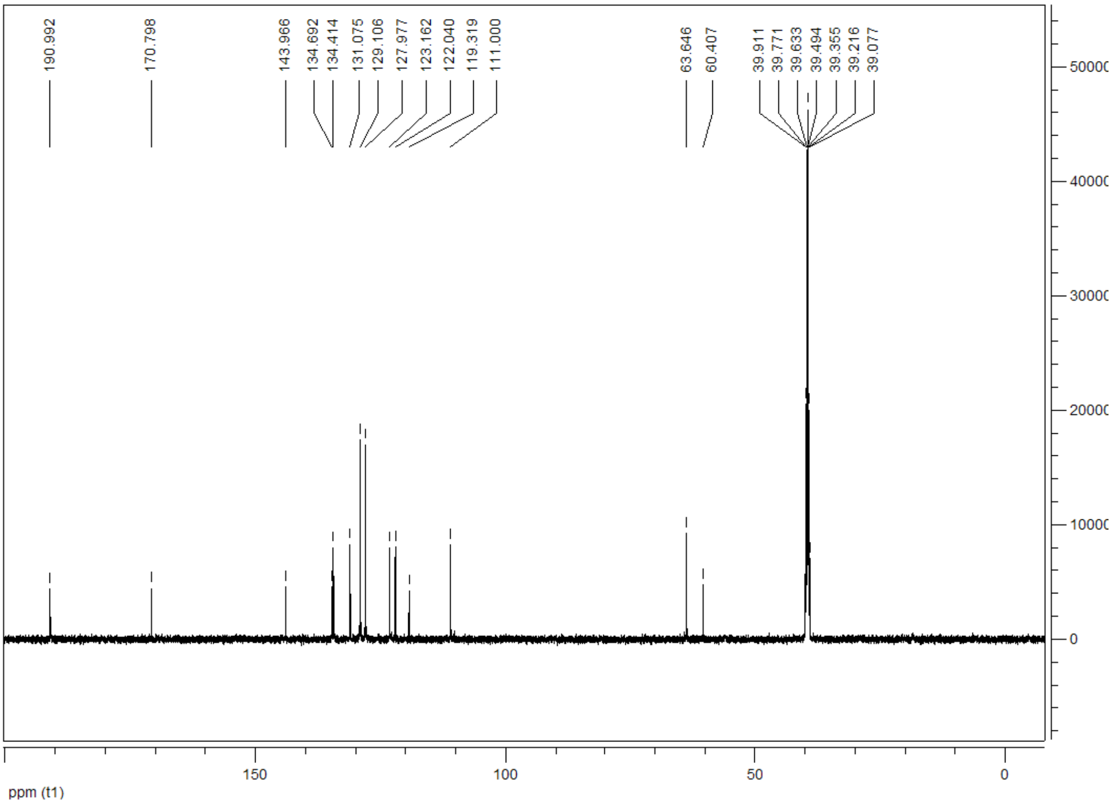

3'-(4-chlorobenzoyl)spiro[indoline-3,2'-oxiran]-2-one (3b)

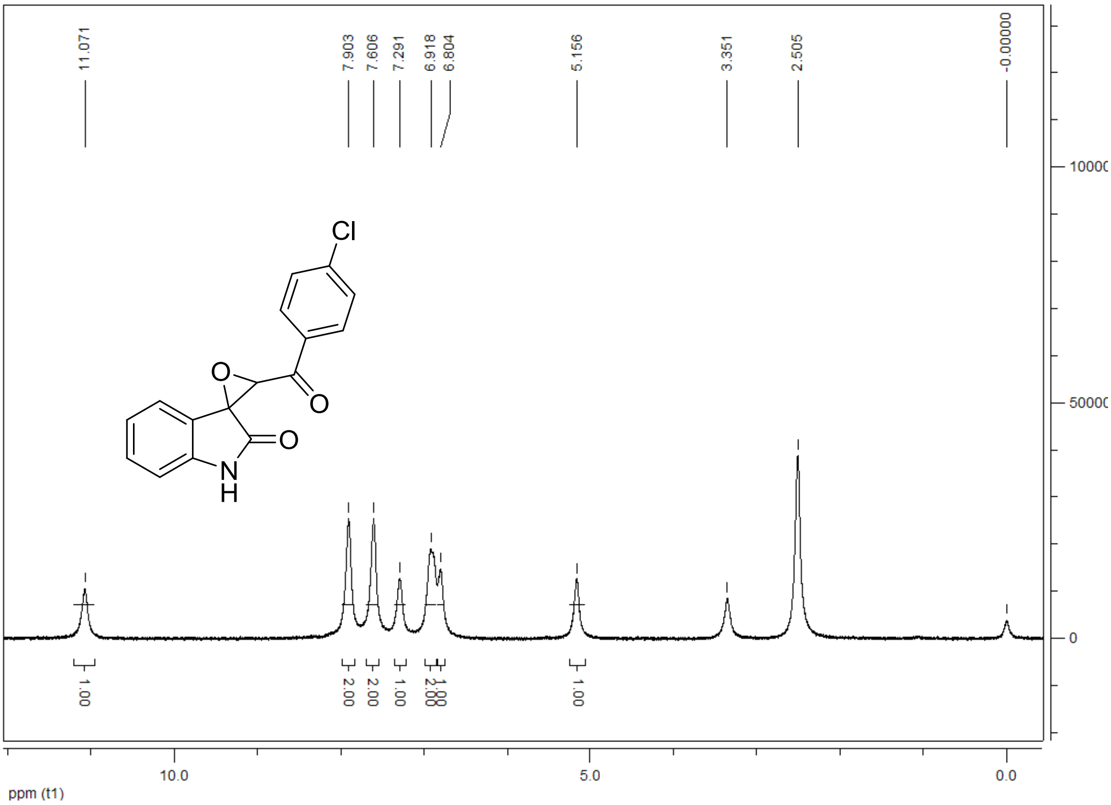

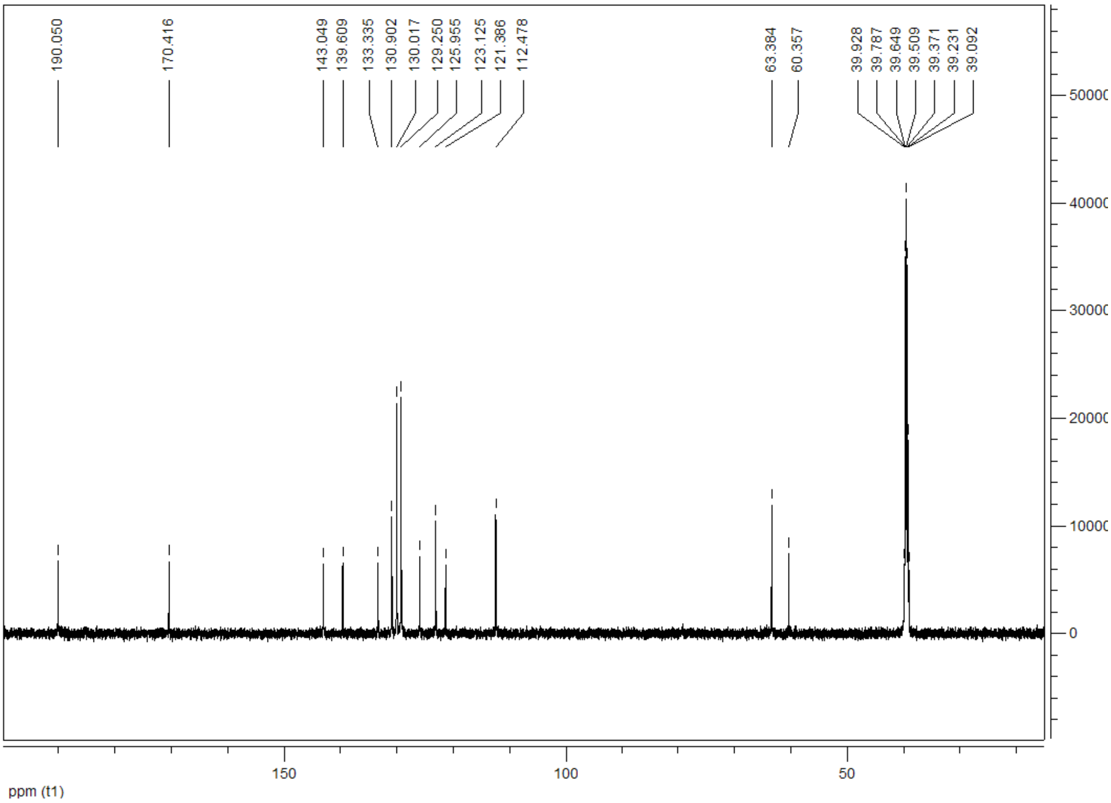

3'-benzoyl-5-methylspiro[indoline-3,2'-oxiran]-2-one (3c)

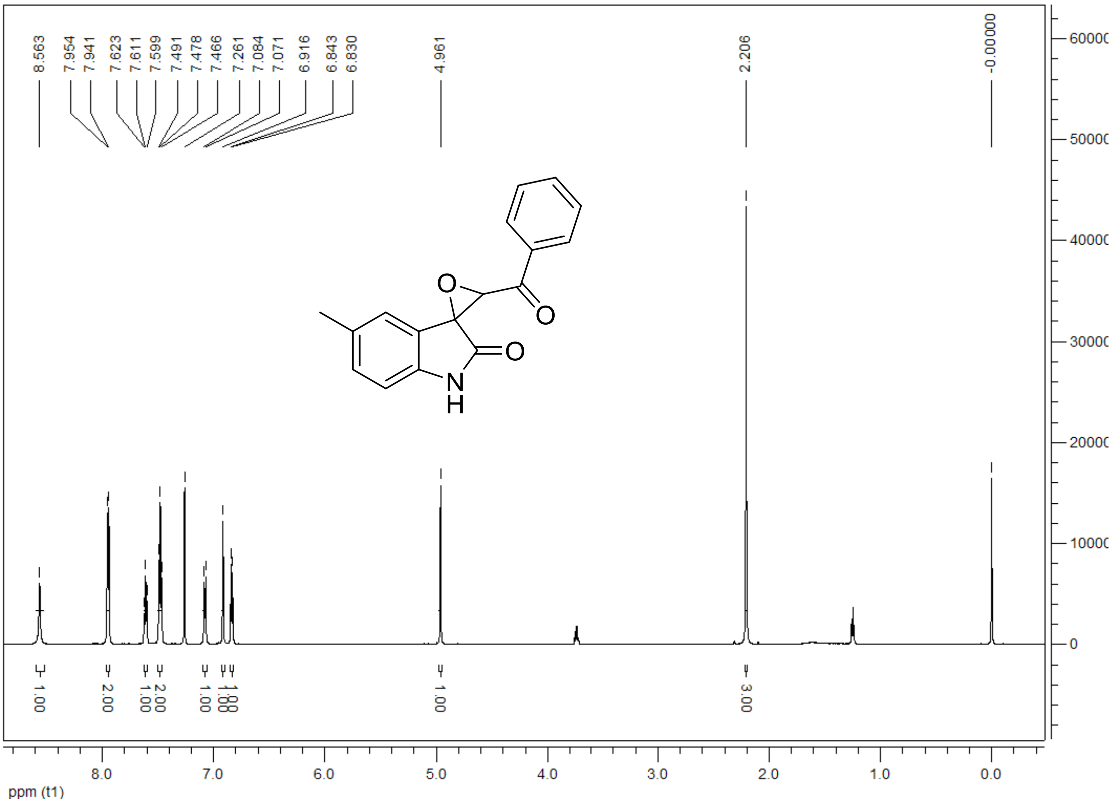

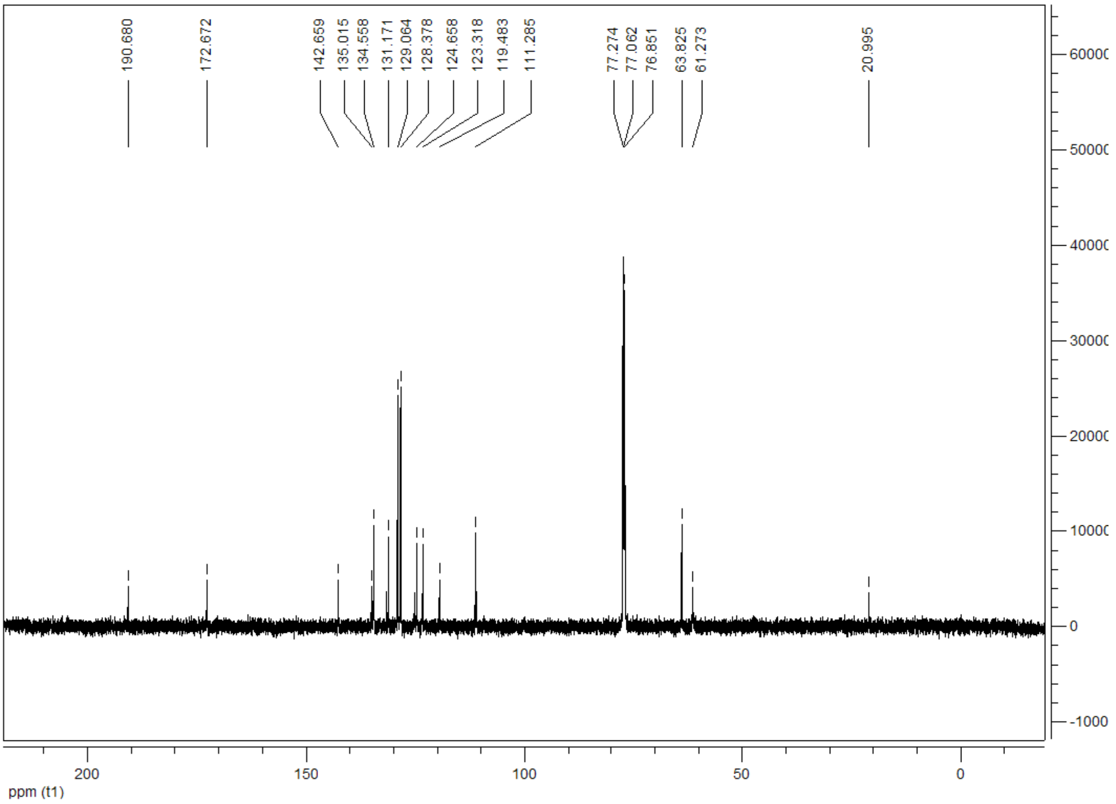

3'-(4-chlorobenzoyl)-5-methylspiro[indoline-3,2'-oxiran]-2-one (3d)

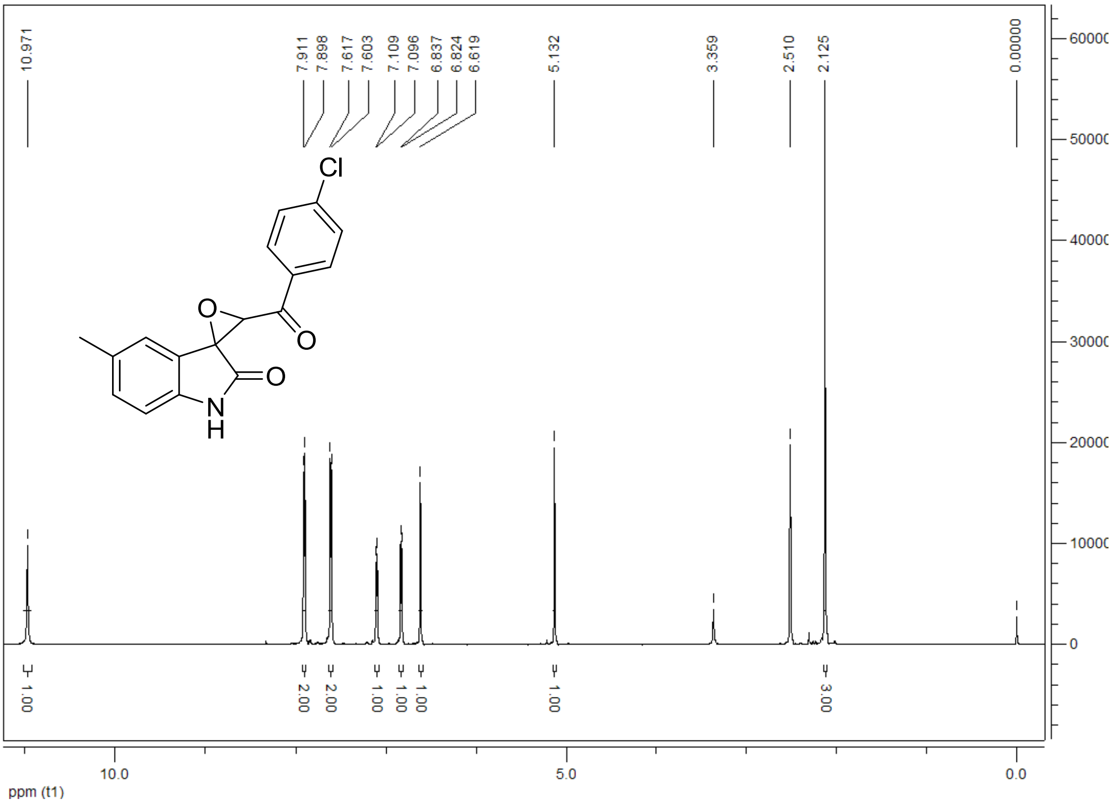

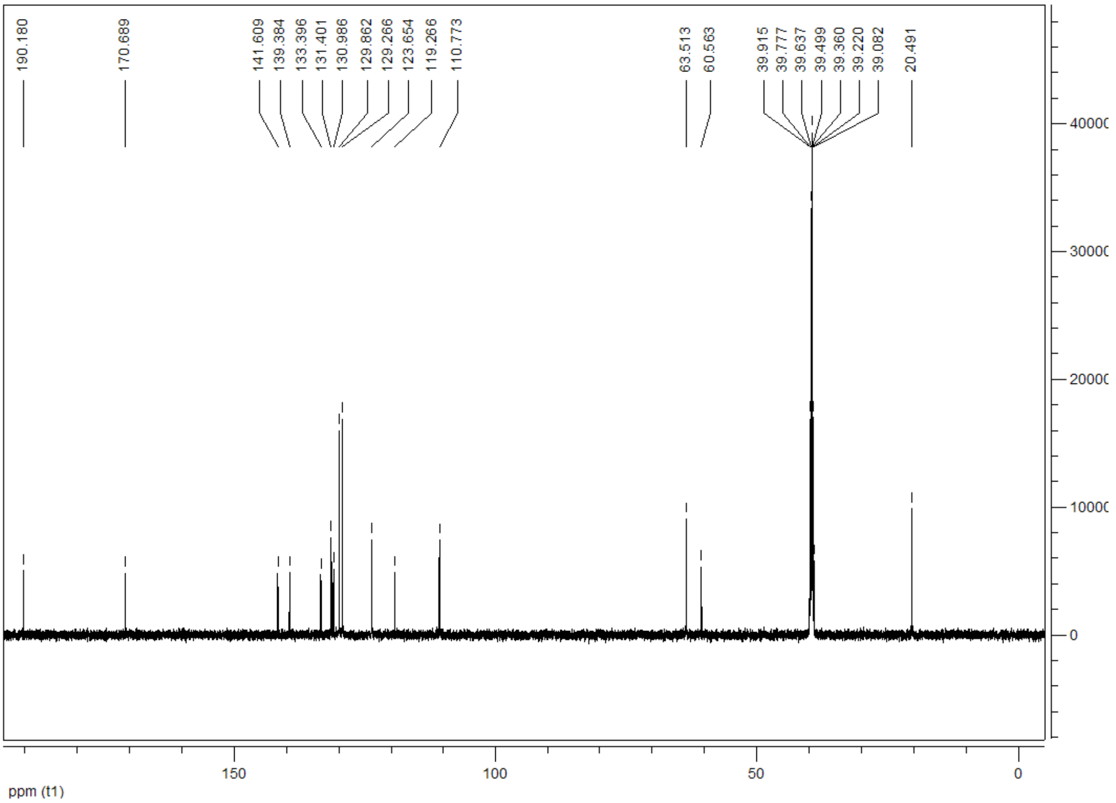

3'-benzoyl-5-fluorospiro[indoline-3,2'-oxiran]-2-one (3e)

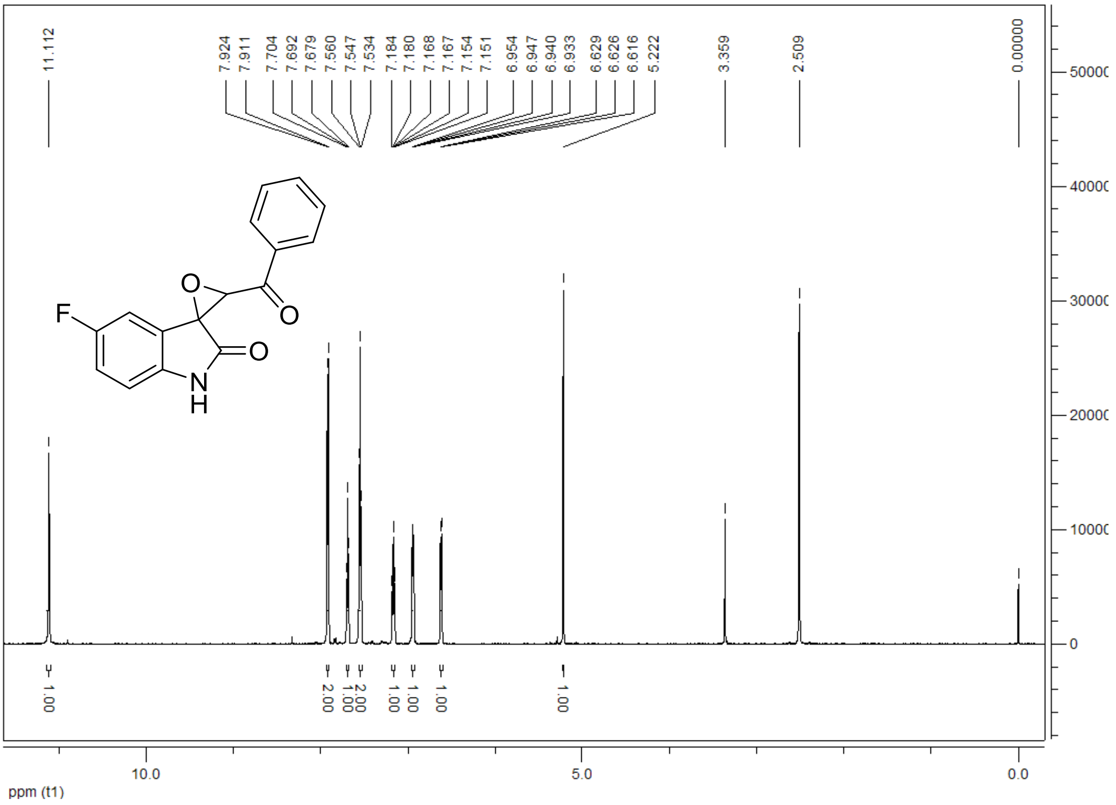

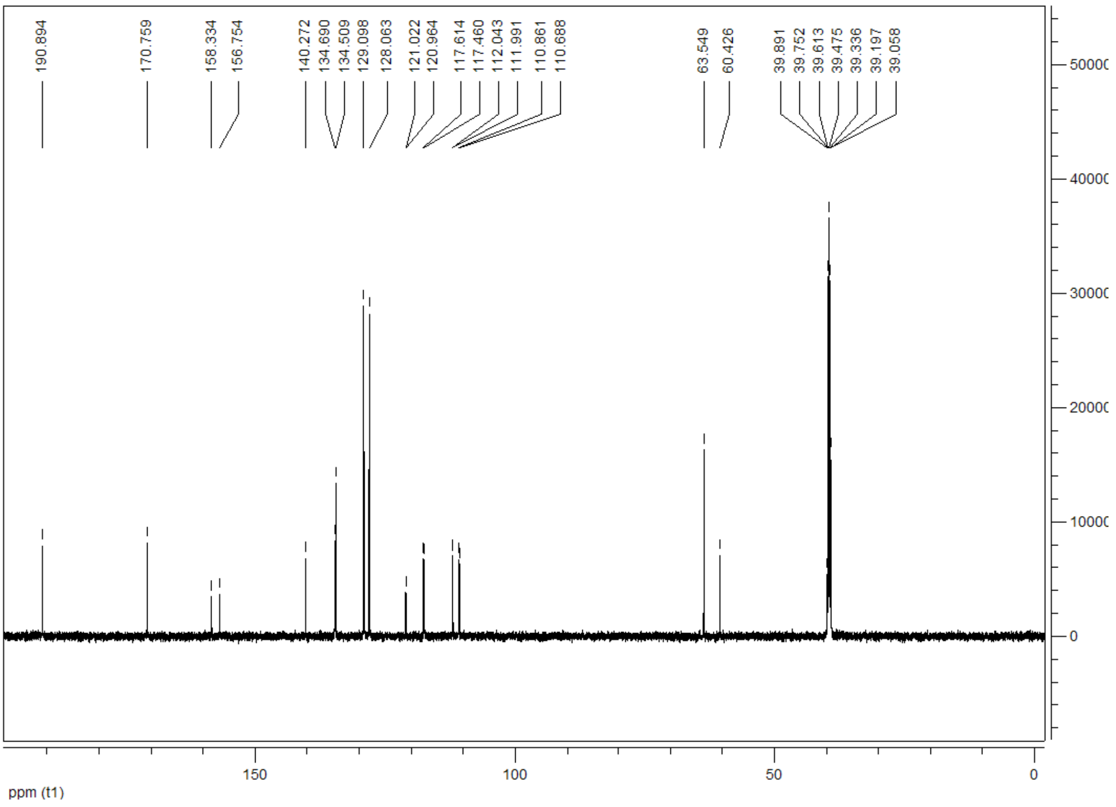

3'-(4-chlorobenzoyl)-5-fluorospiro[indoline-3,2'-oxiran]-2-one (3f)

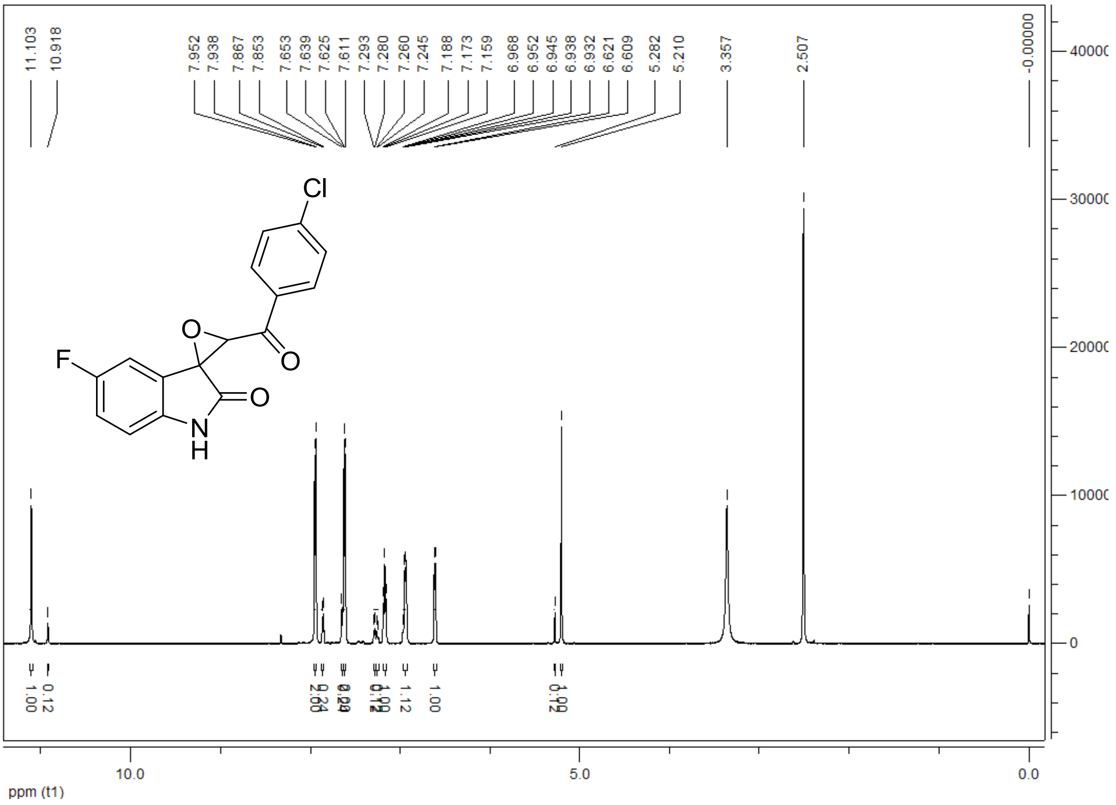

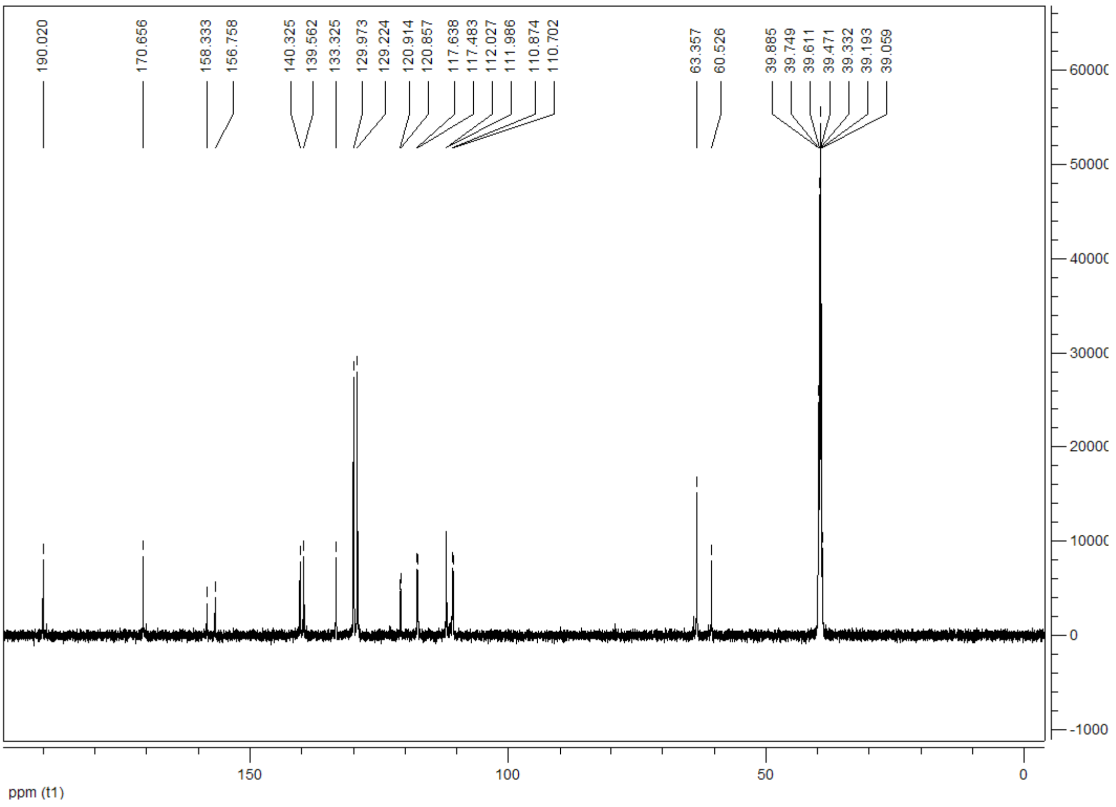

3'-benzoyl-5-chlorospiro[indoline-3,2'-oxiran]-2-one (3g)

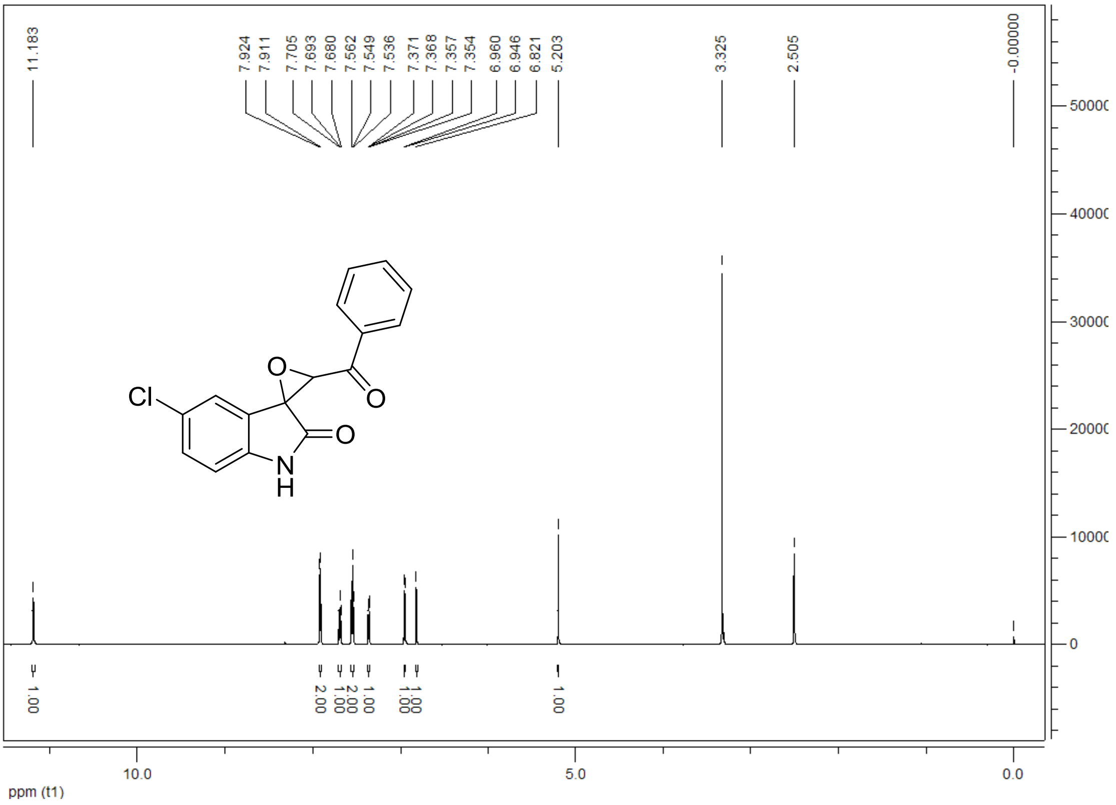

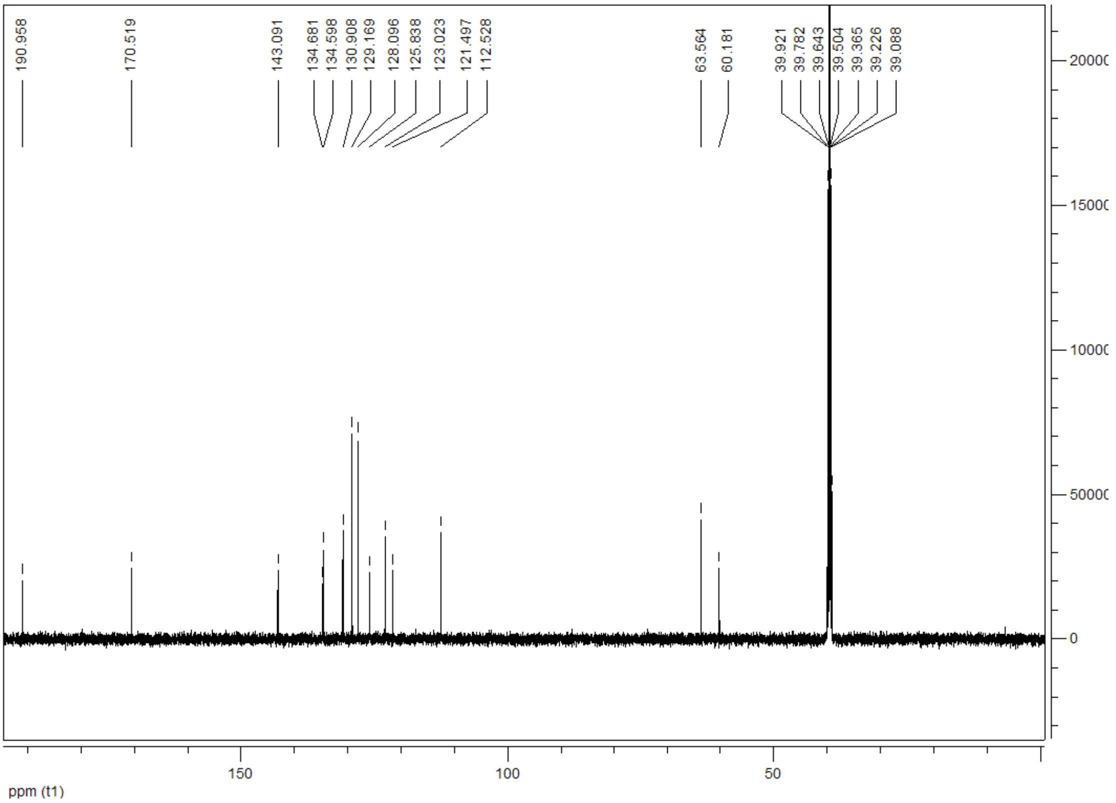

5-chloro-3'-(4-chlorobenzoyl)spiro[indoline-3,2'-oxiran]-2-one (3h)

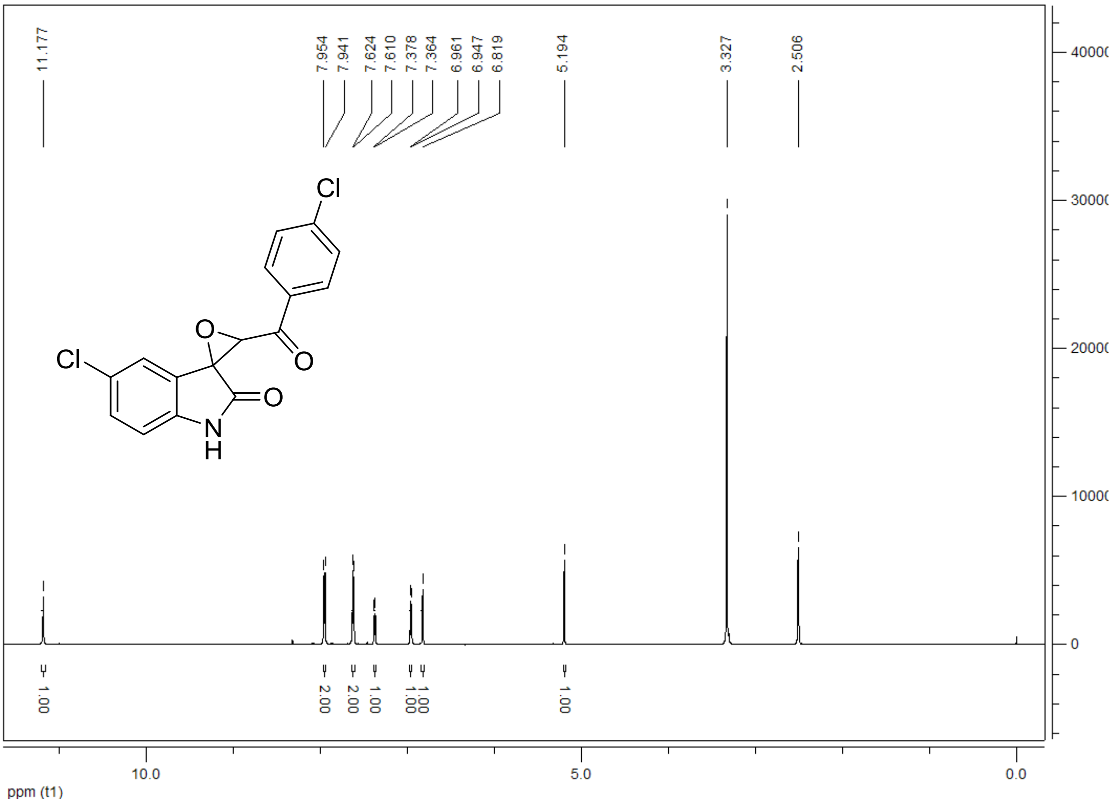

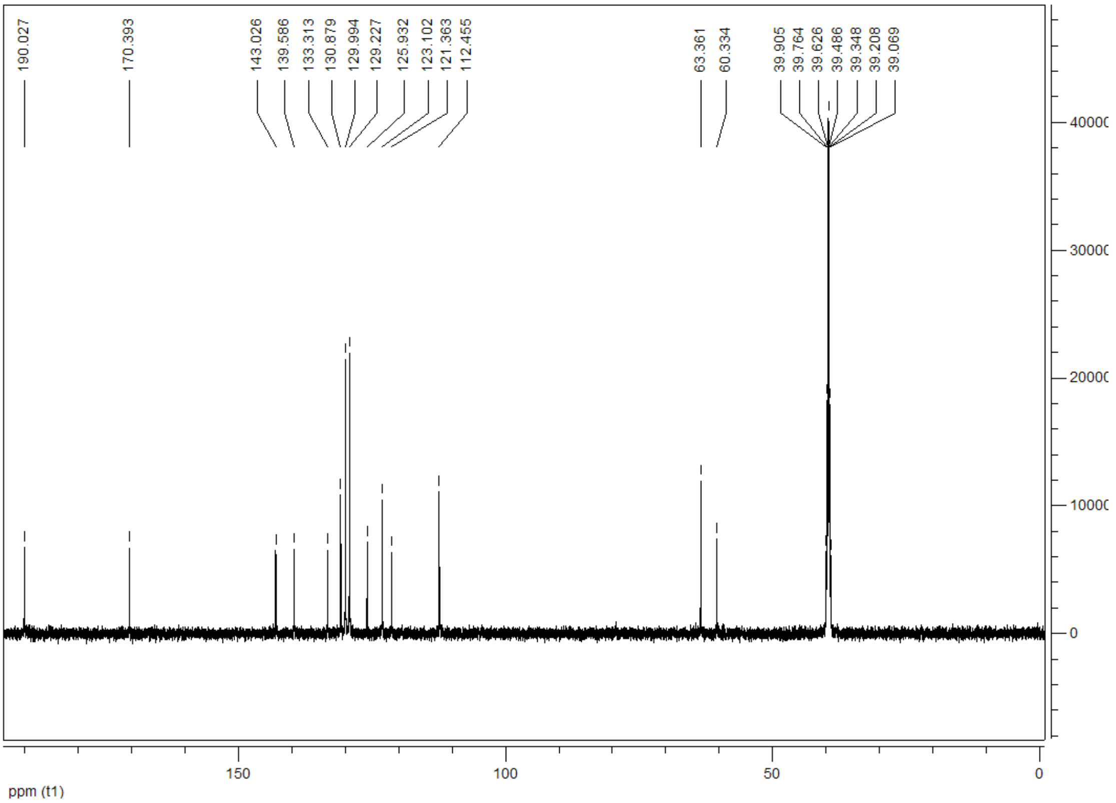

3'-benzoyl-1-(2-oxo-2-phenylethyl)spiro[indoline-3,2'-oxiran]-2-one (4a)

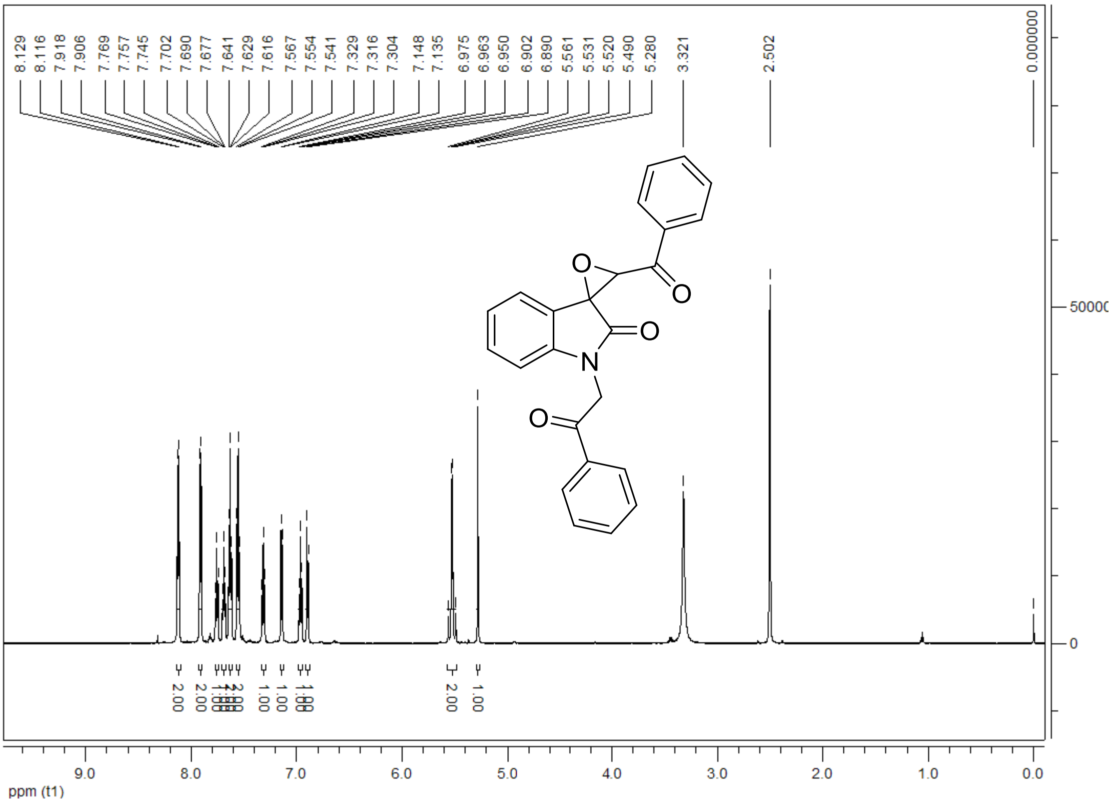

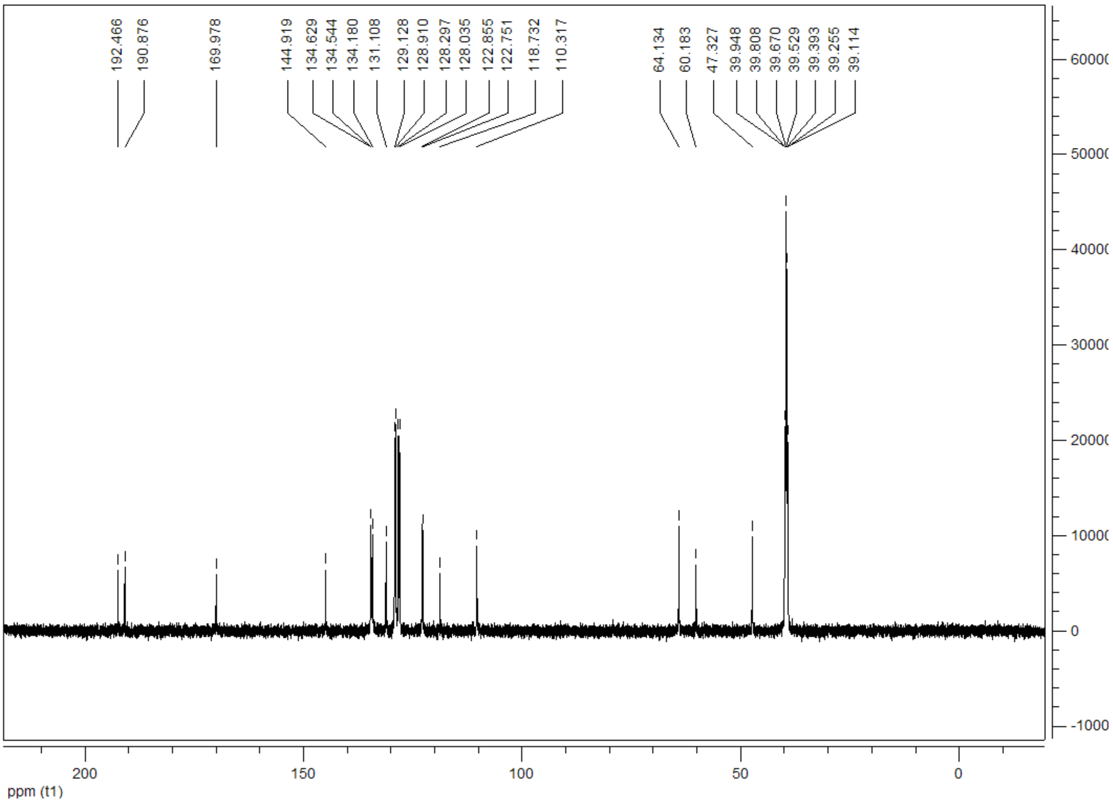

3'-(4-chlorobenzoyl)-1-(2-(4-chlorophenyl)-2-oxoethyl)spiro[indoline-3,2'-oxiran]-2-one (4b)

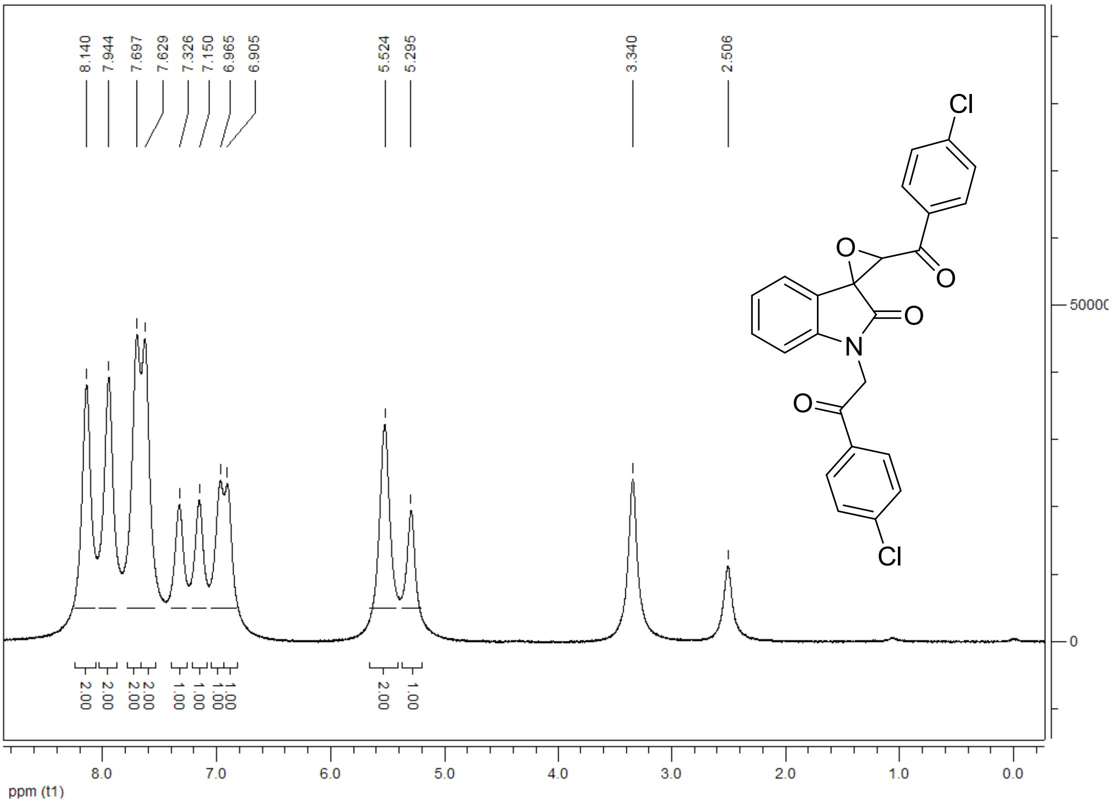

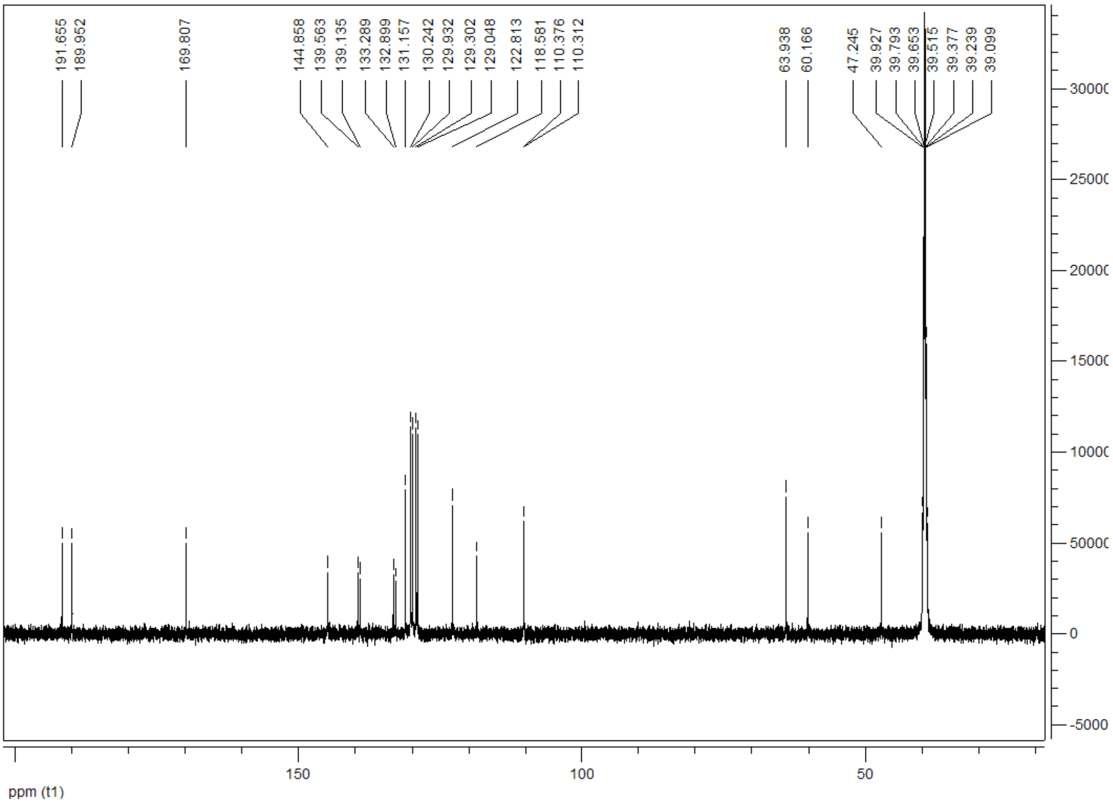

3'-benzoyl-5-methyl-1-(2-oxo-2-phenylethyl)spiro[indoline-3,2'-oxiran]-2-one (4c)

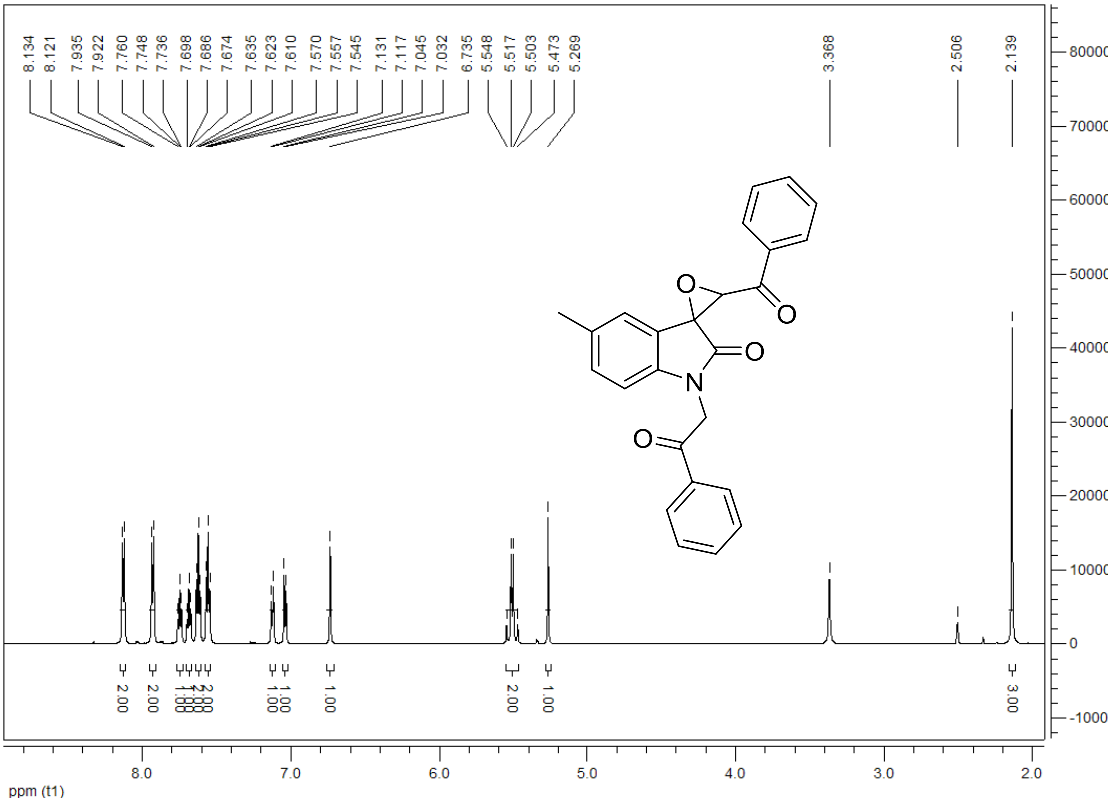

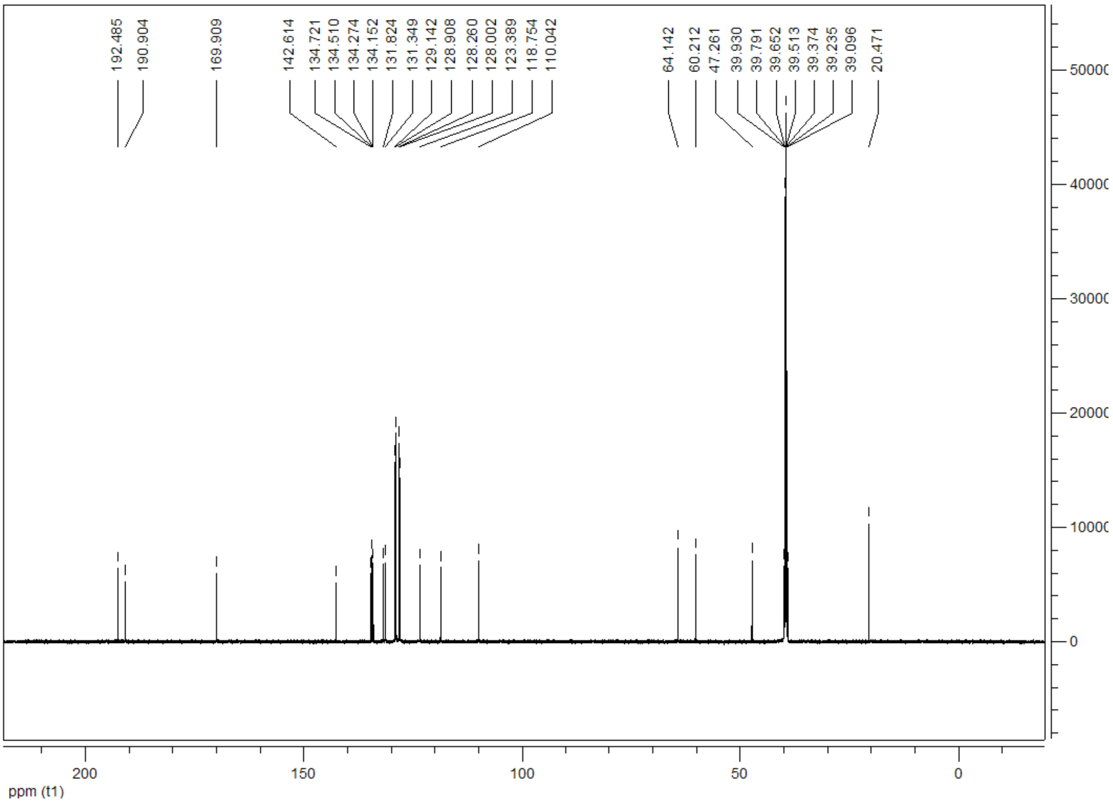

3'-(4-chlorobenzoyl)-1-(2-(4-chlorophenyl)-2-oxoethyl)-5-methylspiro[indoline-3,2'-oxiran]-2-one (4d)

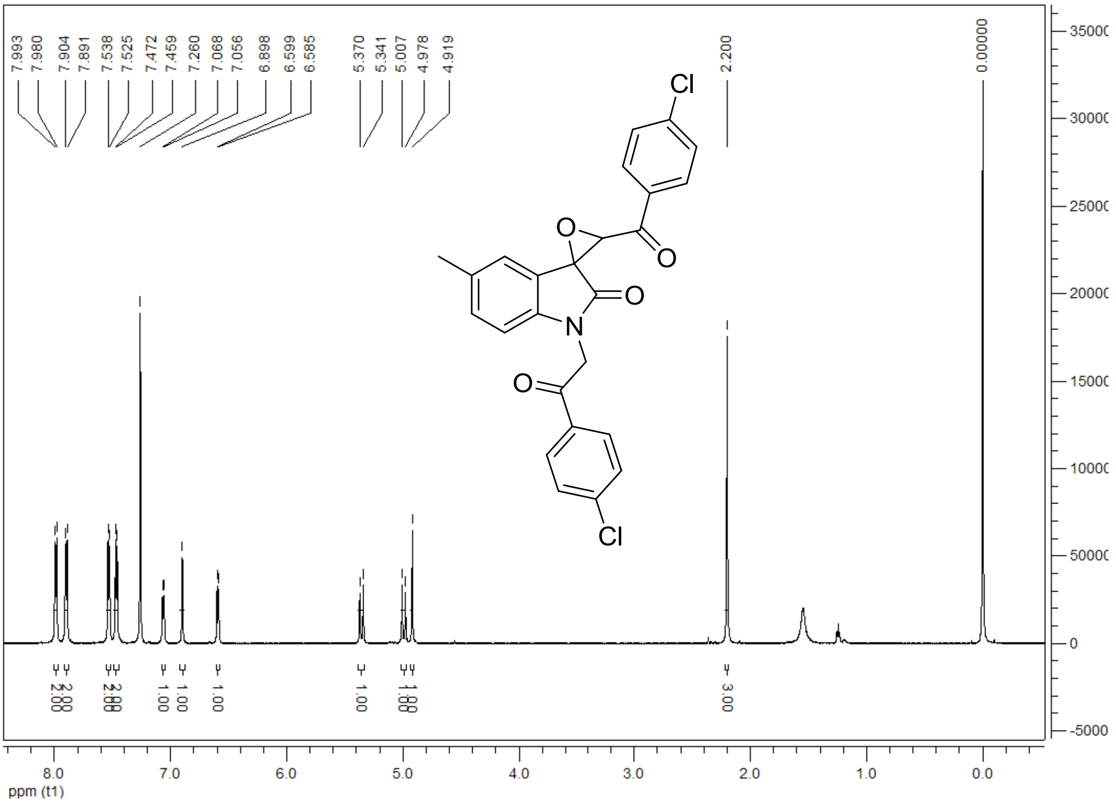

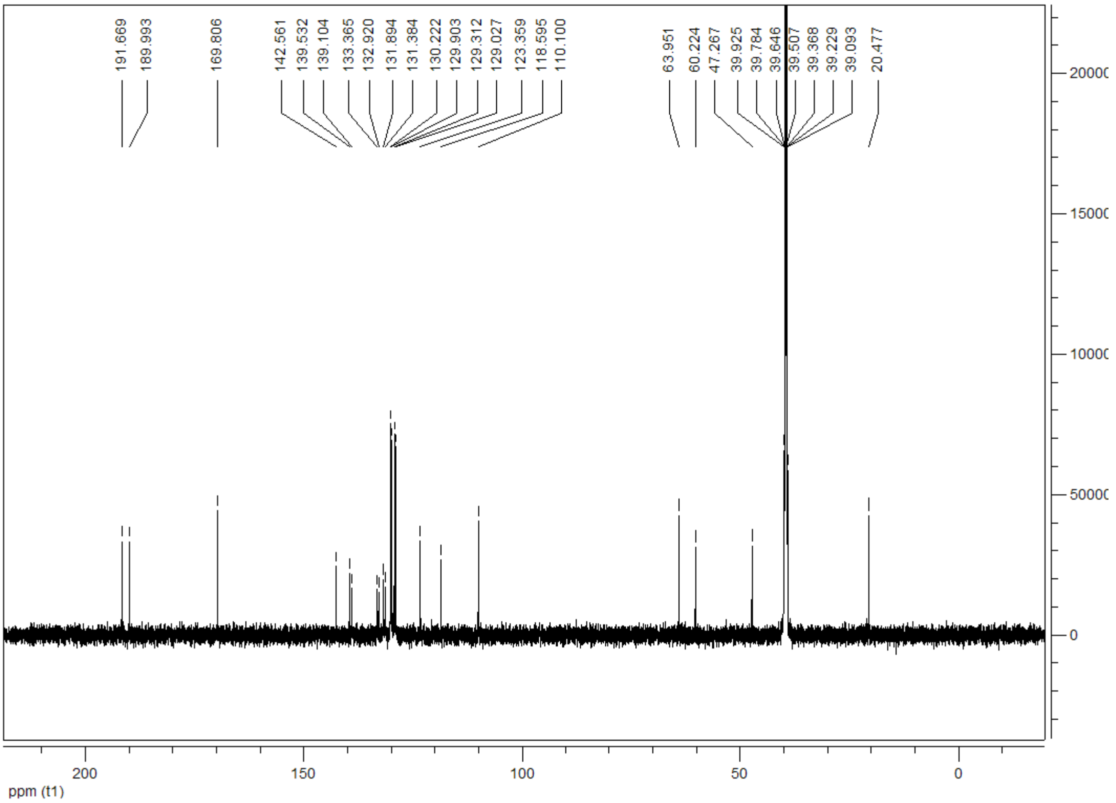

5-chloro-3'-(4-chlorobenzoyl)-1-(2-(4-chlorophenyl)-2-oxoethyl)spiro[indoline-3,2'-oxiran]-2-one (4e)

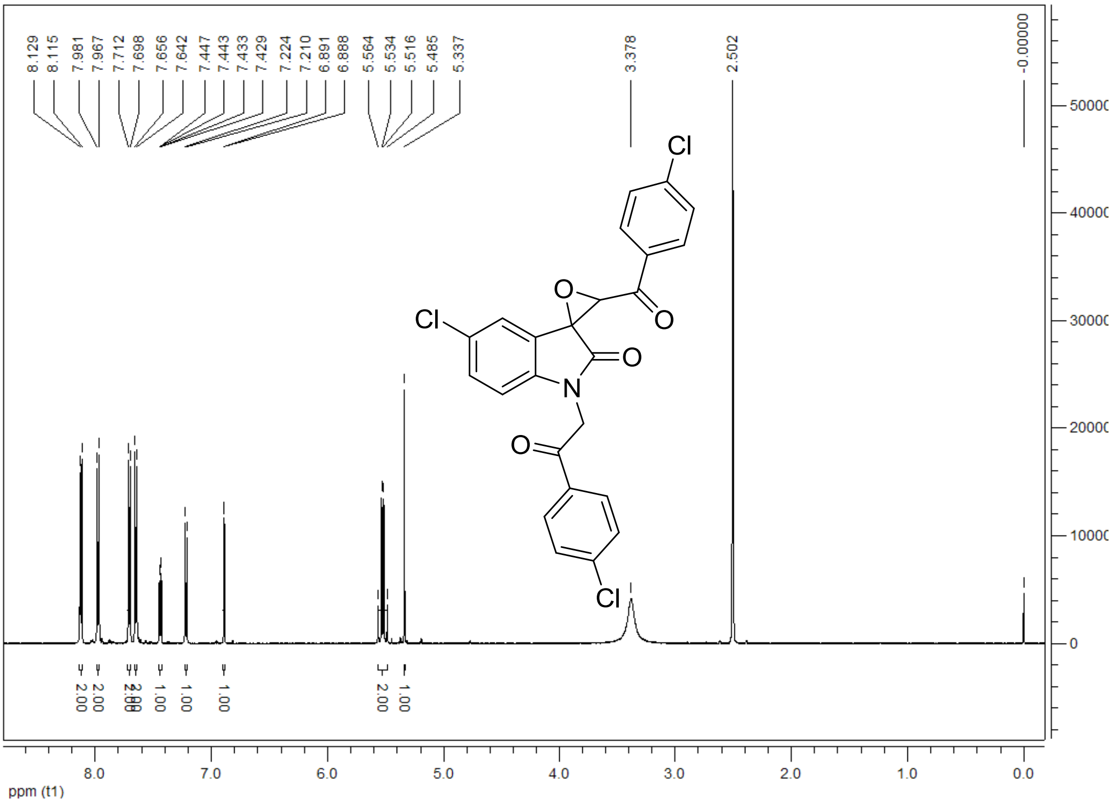

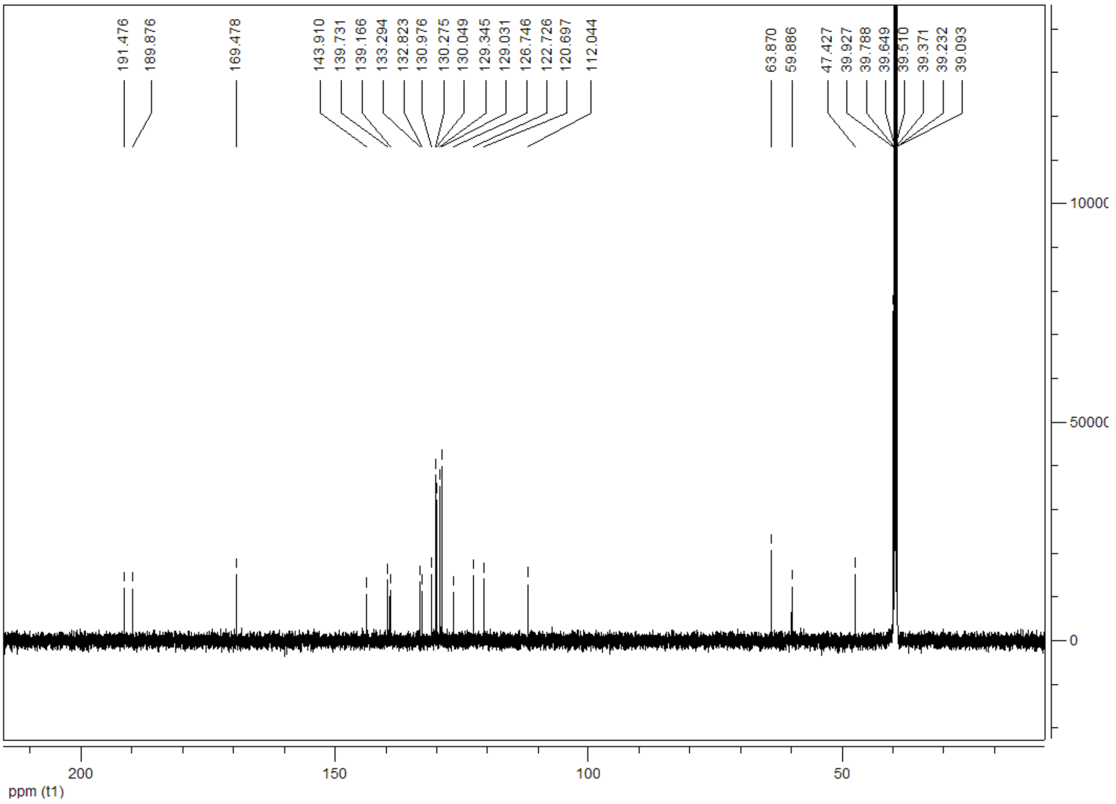

3'-benzoyl-1-benzylspiro[indoline-3,2'-oxiran]-2-one (5a)

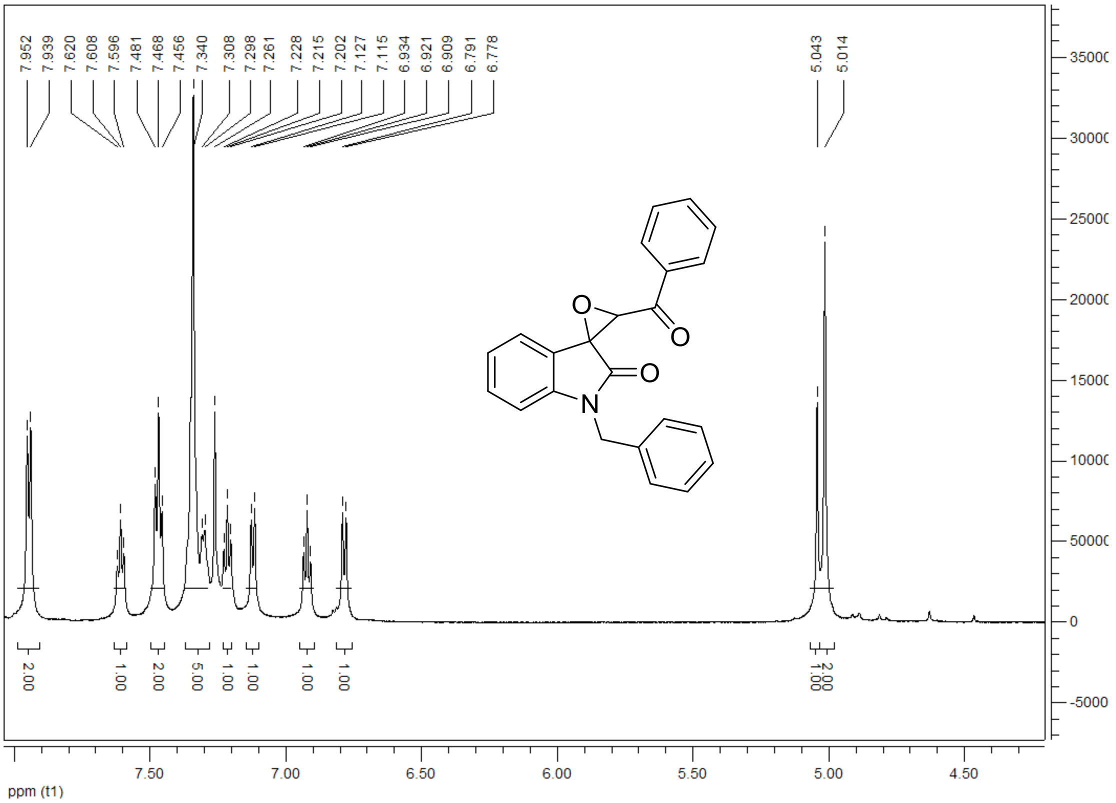

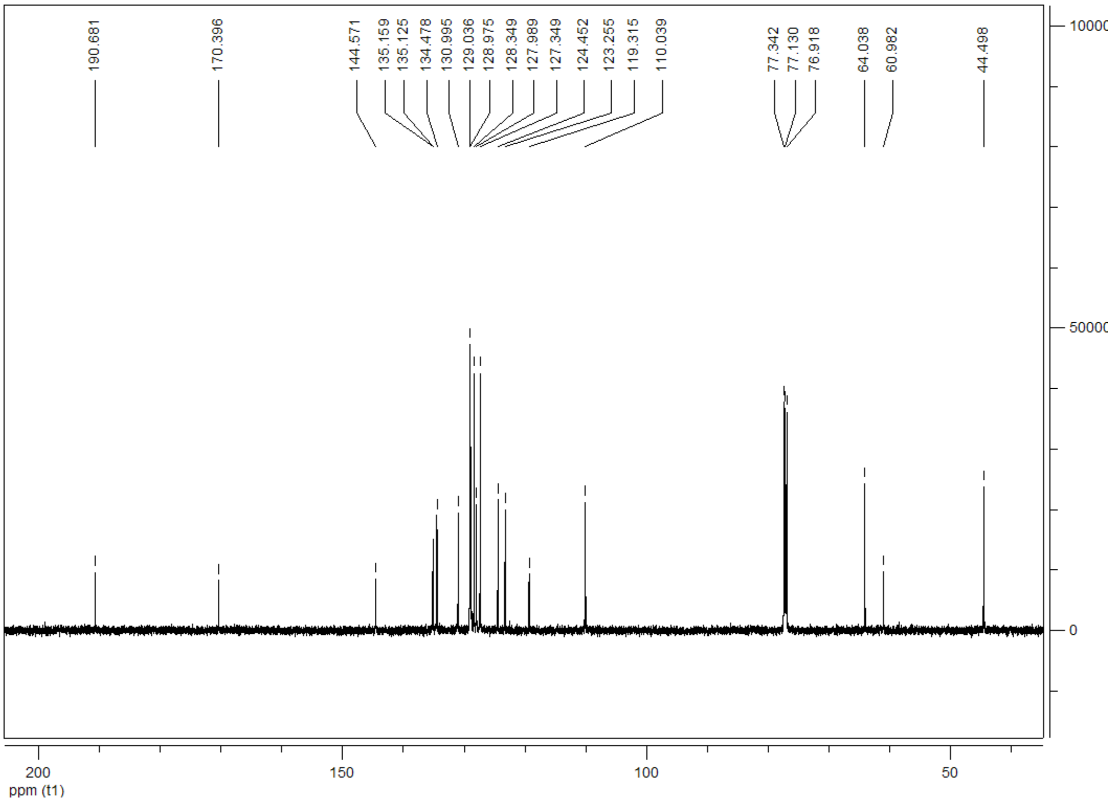

1-benzyl-3'-(4-chlorobenzoyl)spiro[indoline-3,2'-oxiran]-2-one (5b)

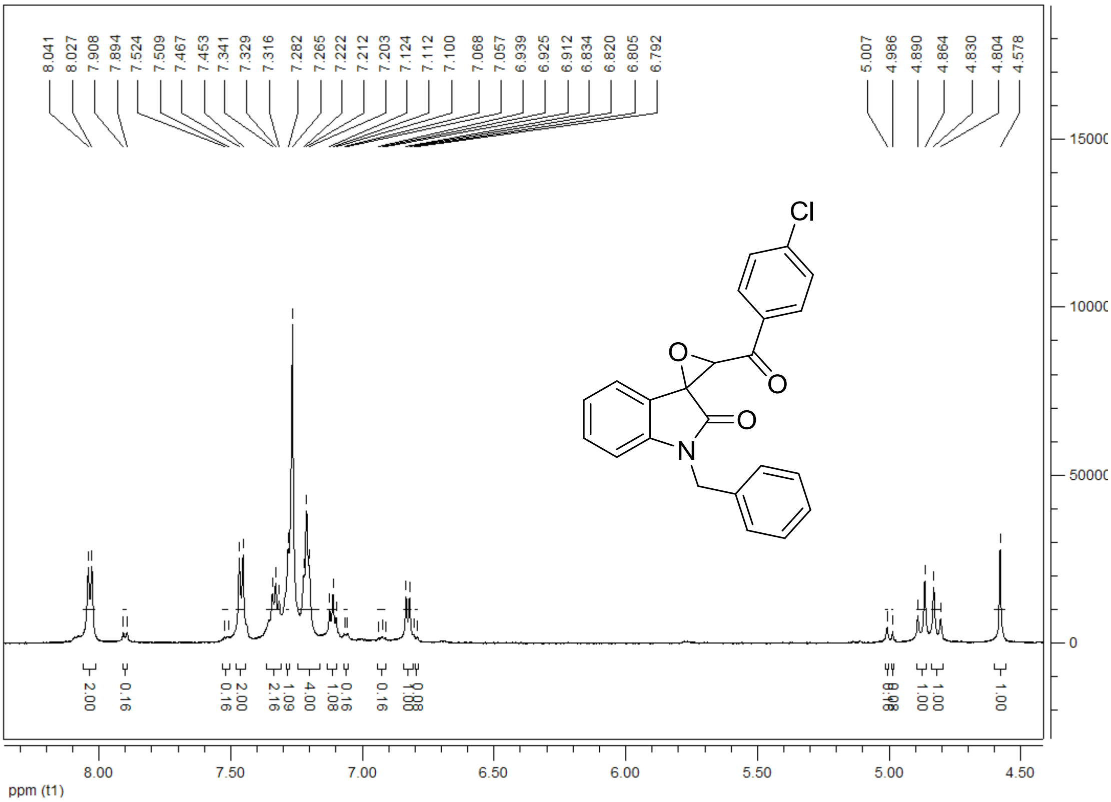

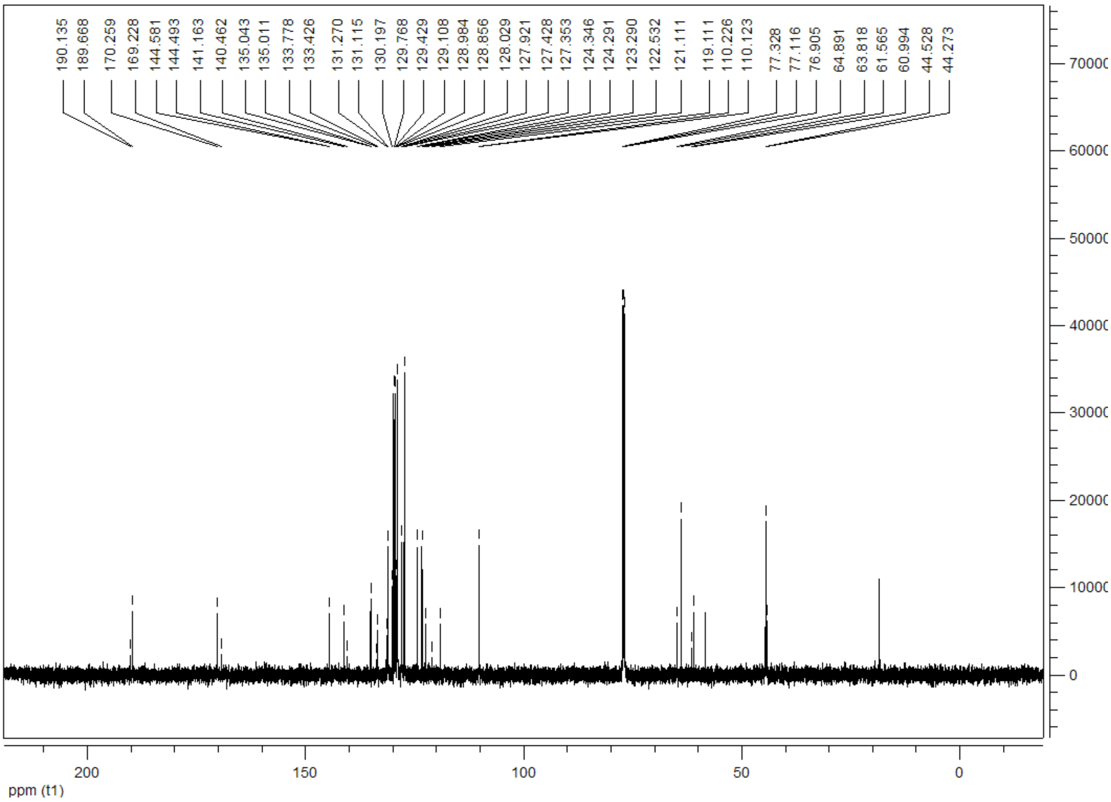

3'-benzoyl-1-butylspiro[indoline-3,2'-oxiran]-2-one (5c)

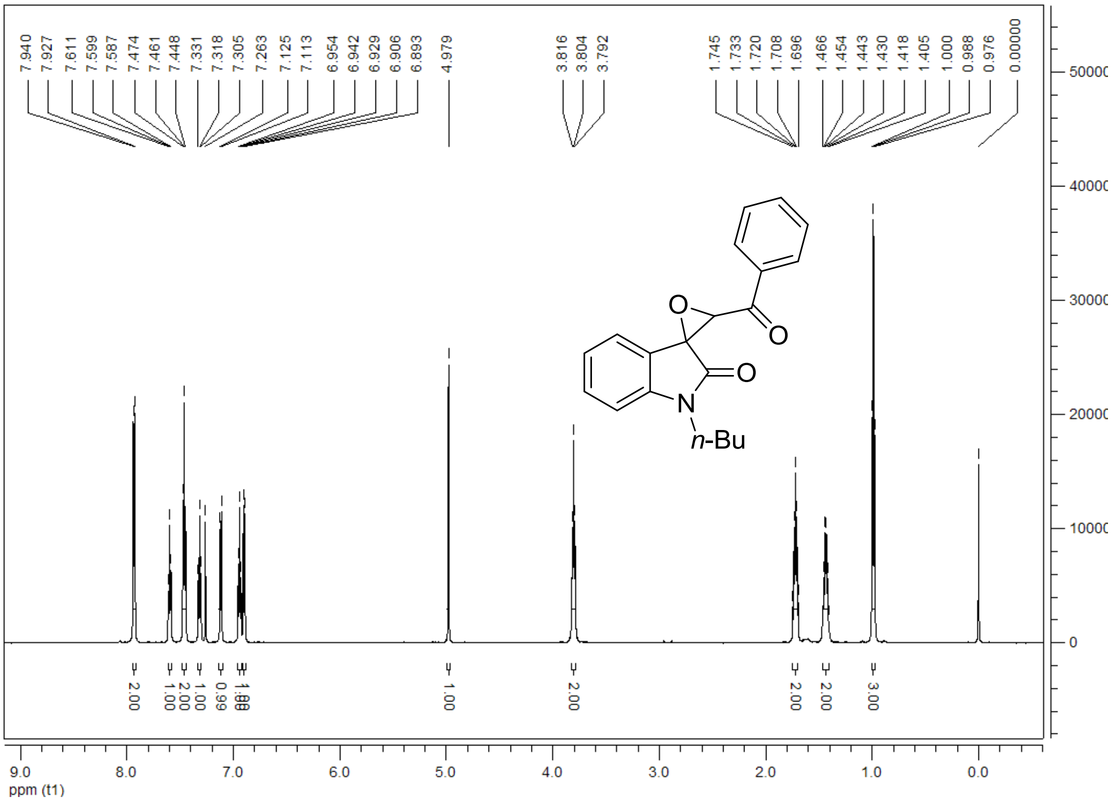

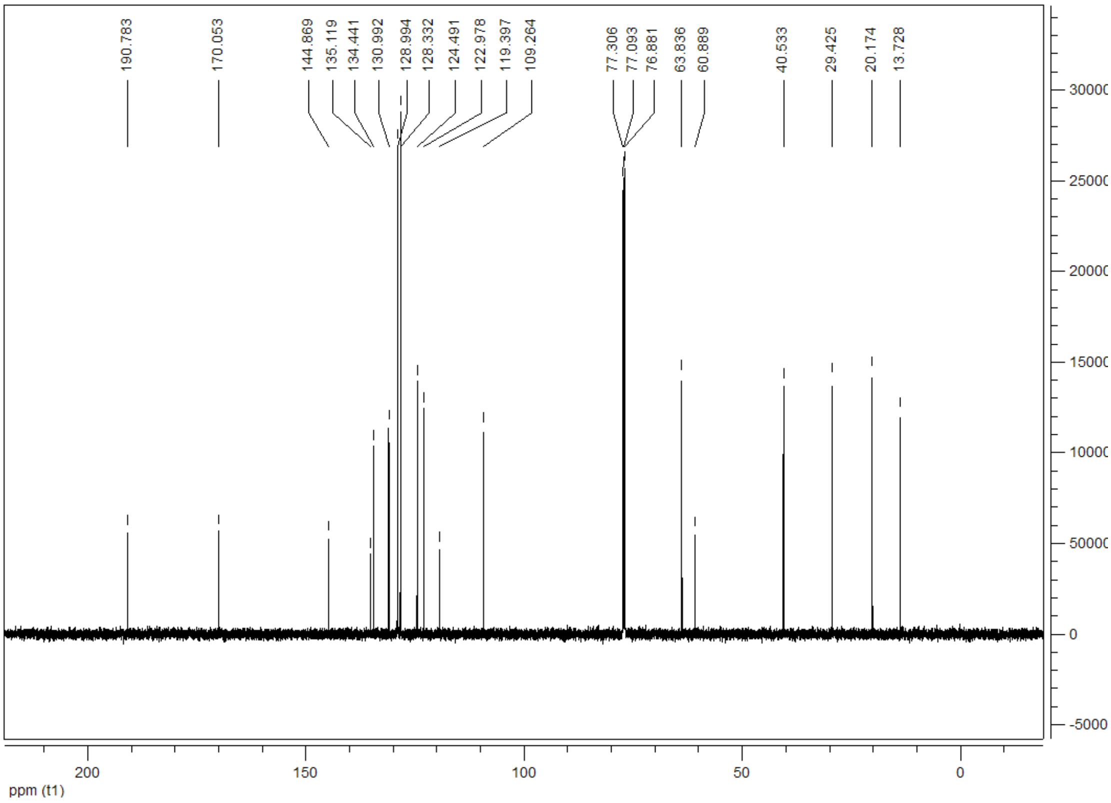

1-butyl-3'-(4-chlorobenzoyl)spiro[indoline-3,2'-oxiran]-2-one (5d)

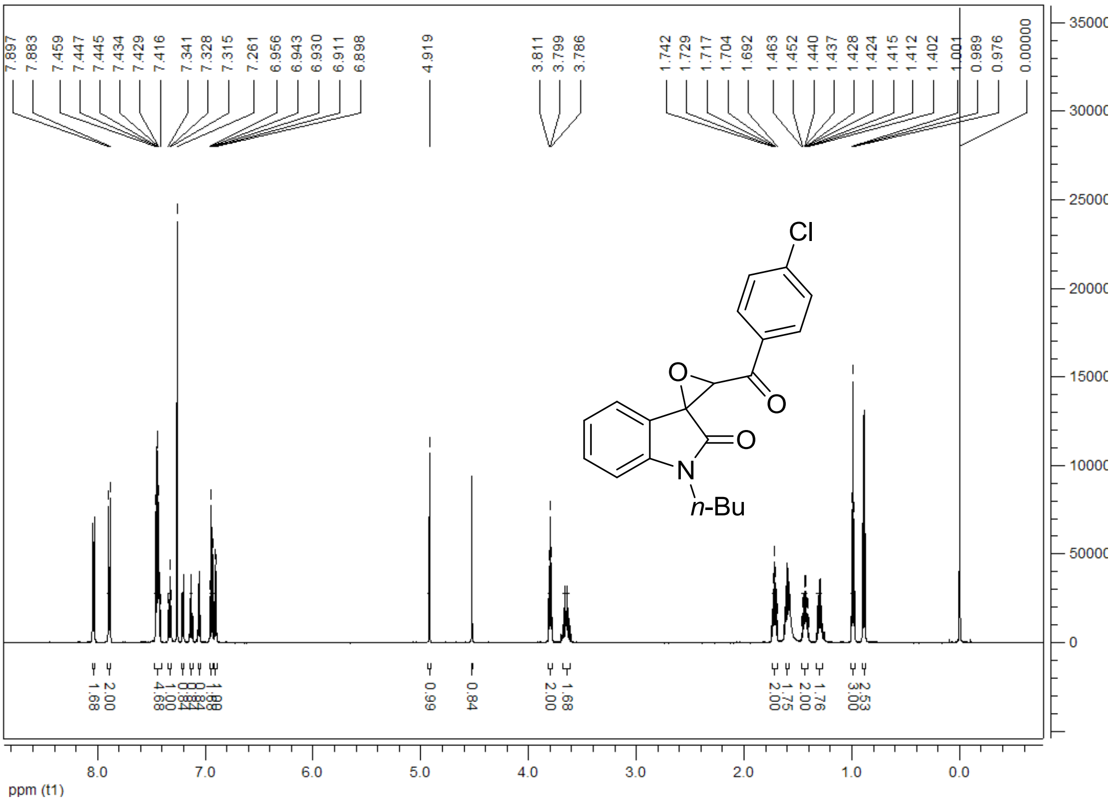

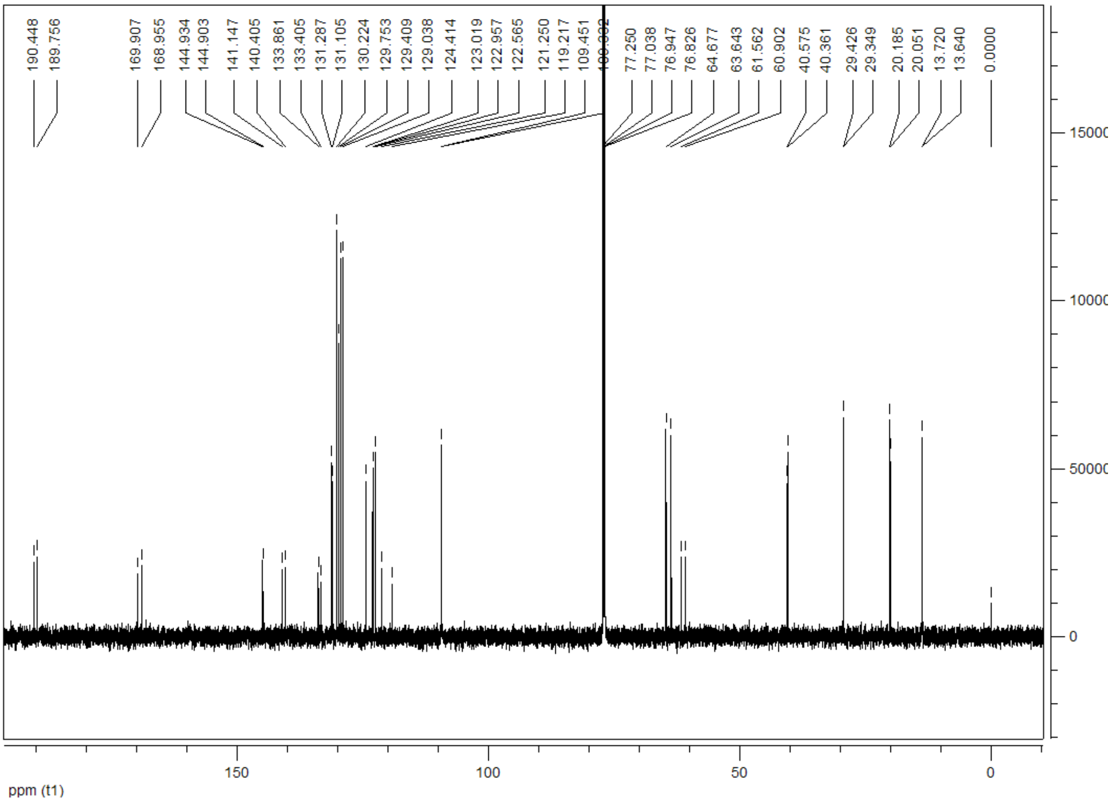

3'-benzoyl-1-benzyl-5-methylspiro[indoline-3,2'-oxiran]-2-one (5e)

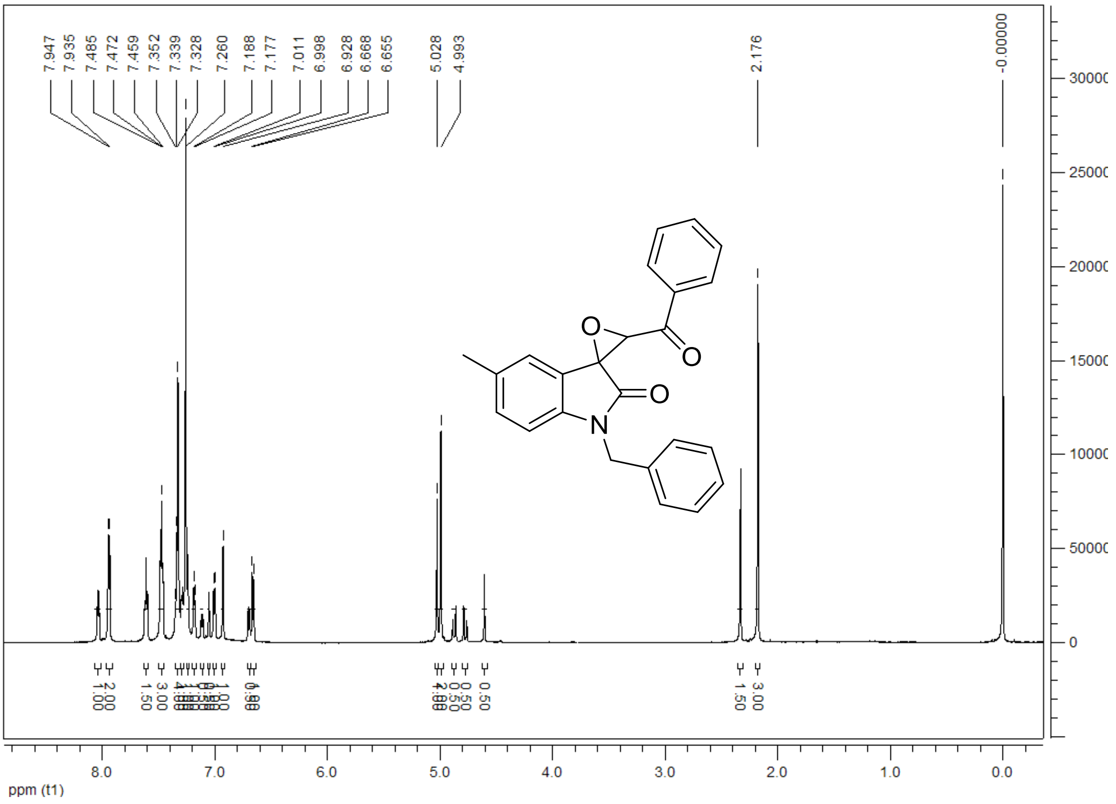

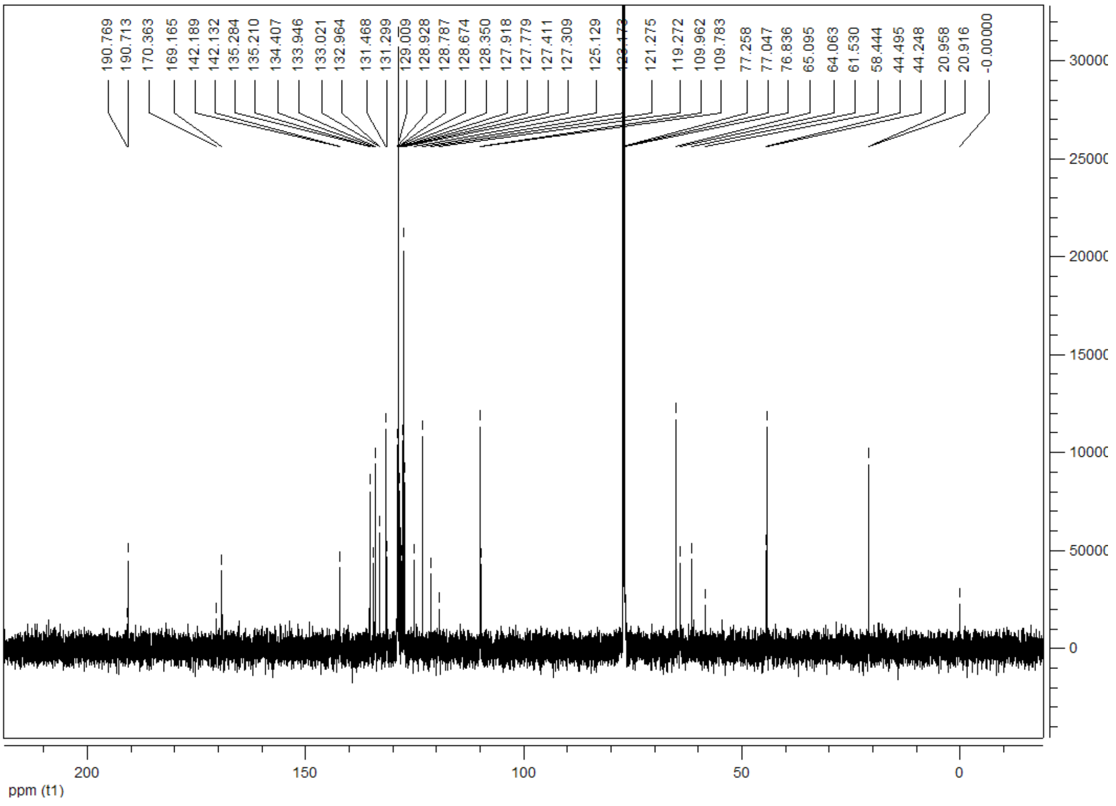

1-benzyl-3'-(4-chlorobenzoyl)-5-methylspiro[indoline-3,2'-oxiran]-2-one (5f)

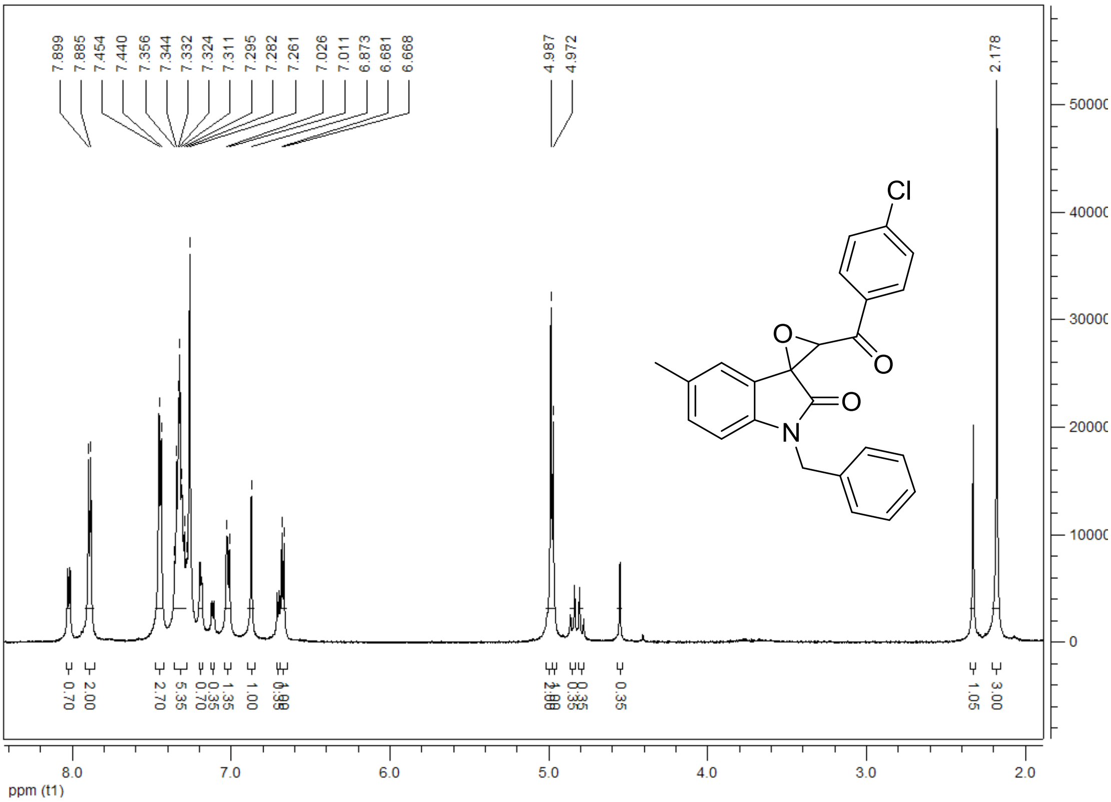

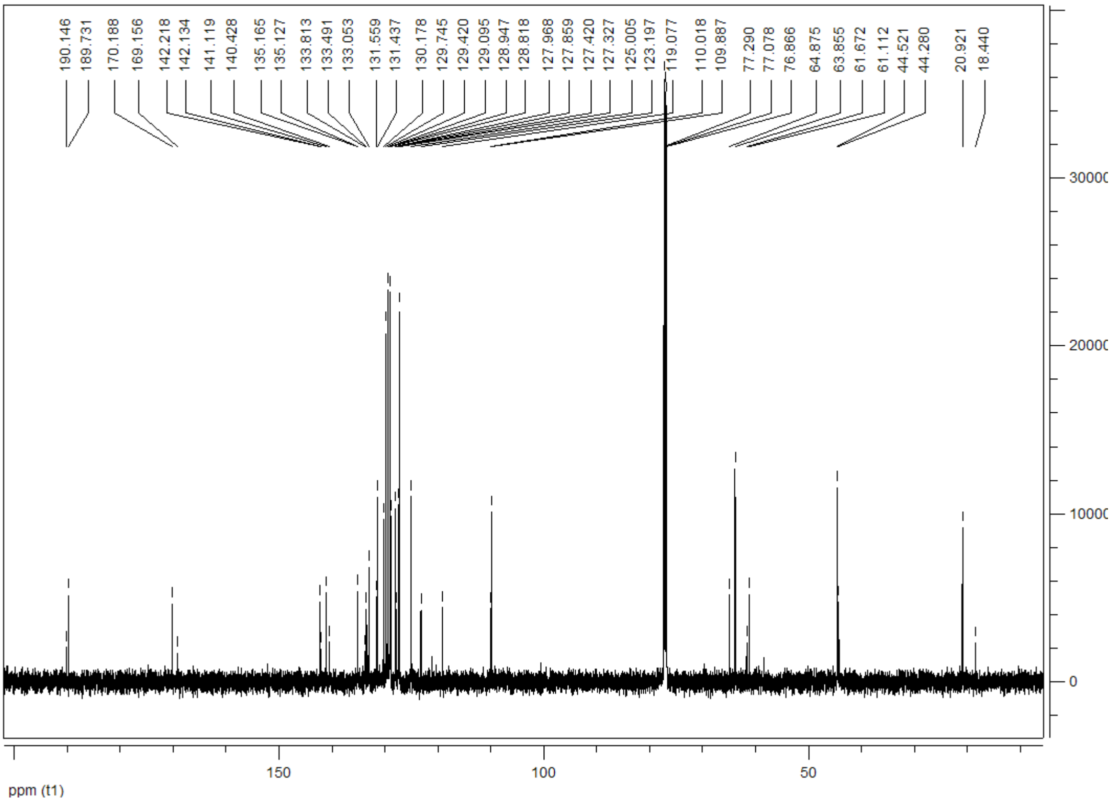

3'-benzoyl-1-butyl-5-methylspiro[indoline-3,2'-oxiran]-2-one (5g)

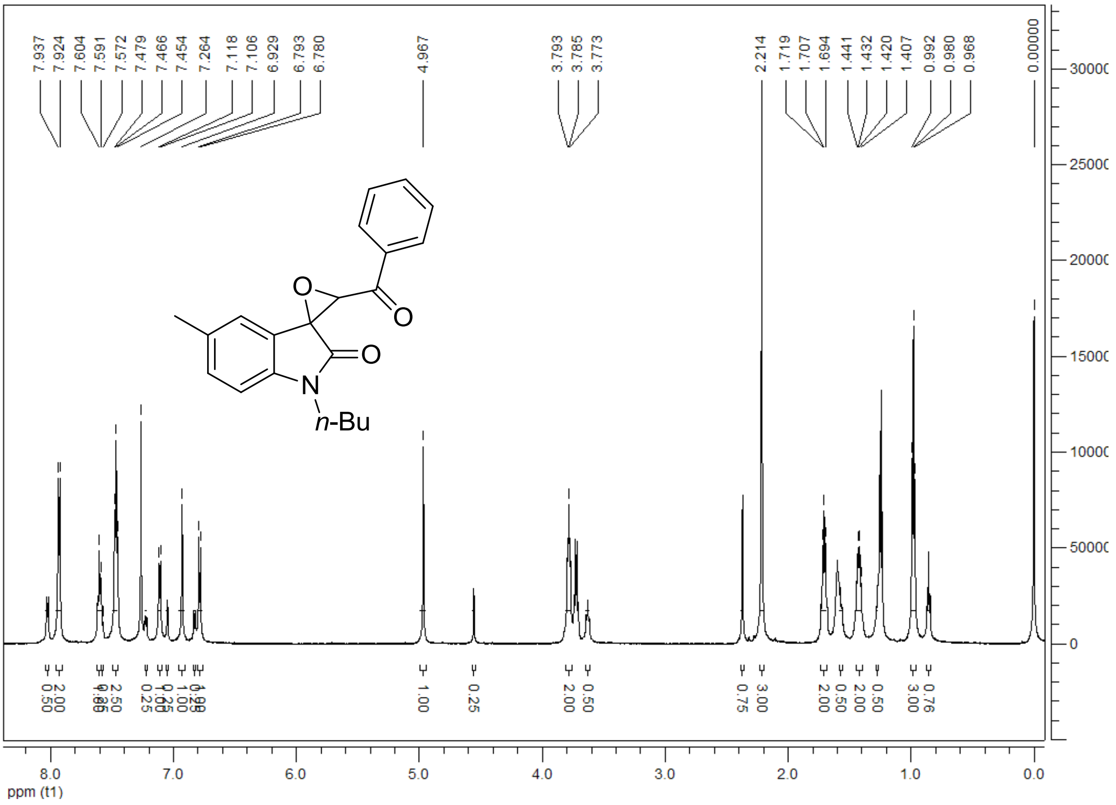

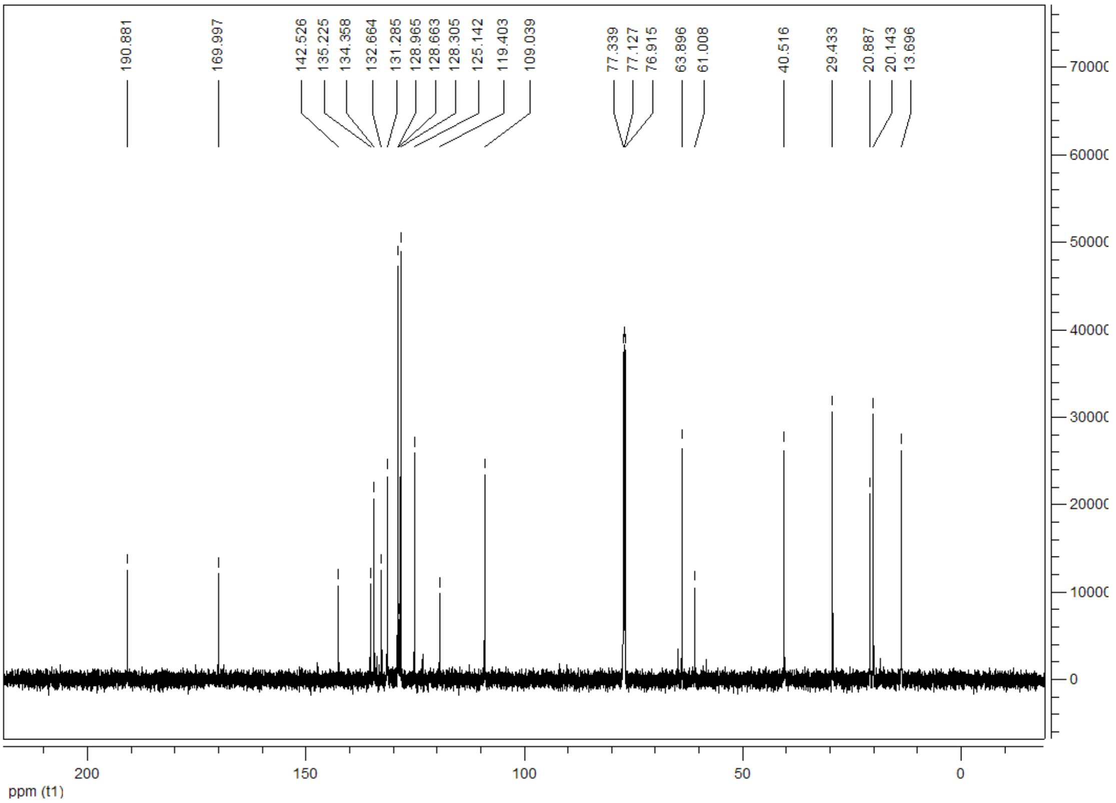

1-butyl-3'-(4-chlorobenzoyl)-5-methylspiro[indoline-3,2'-oxiran]-2-one (5h)

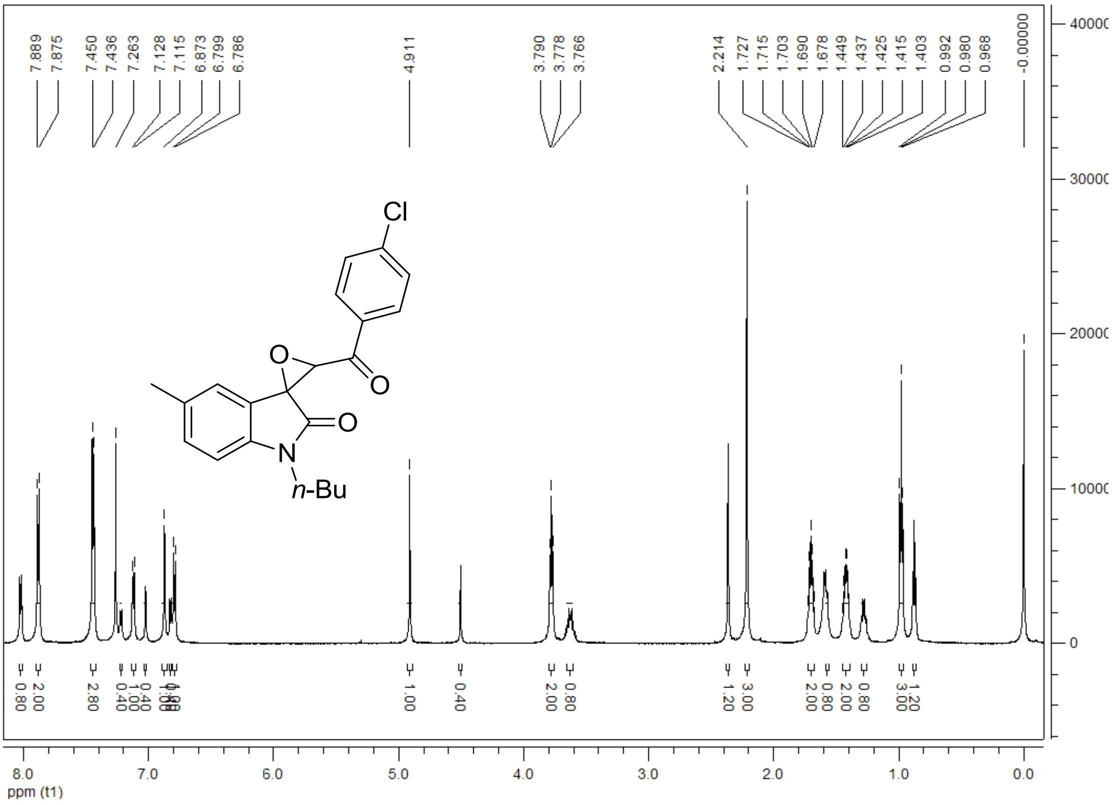

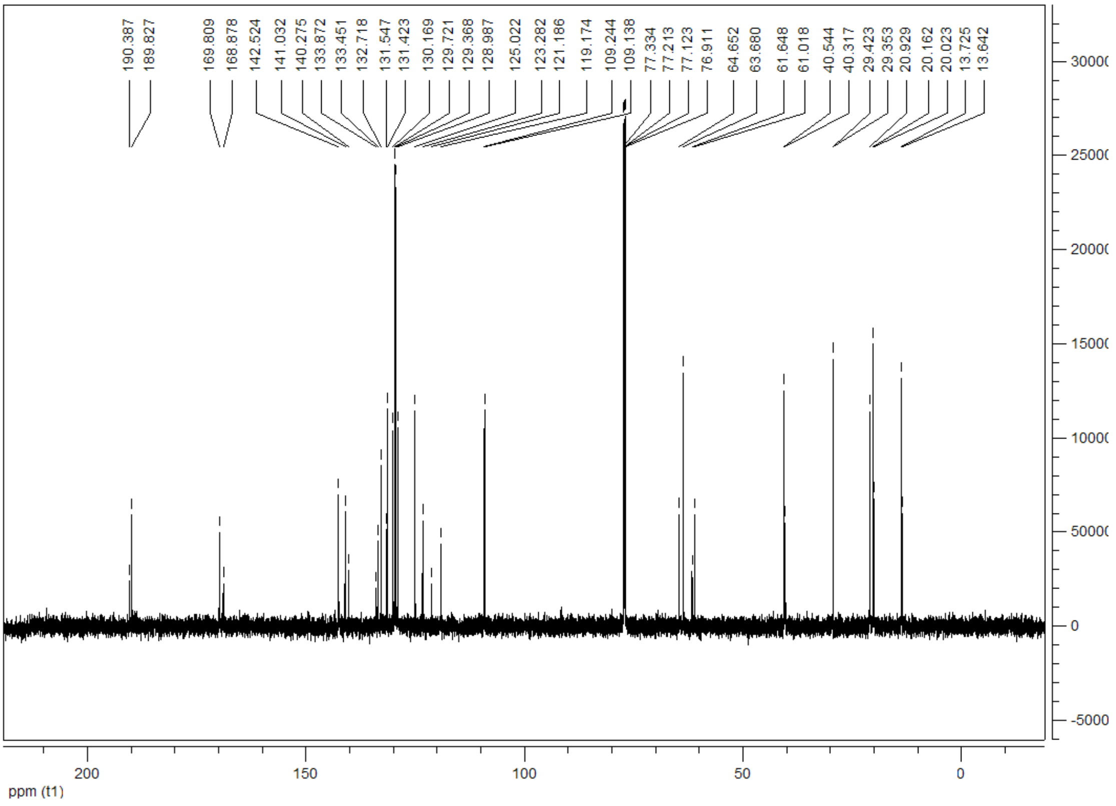

3'-benzoyl-1-benzyl-5-fluorospiro[indoline-3,2'-oxiran]-2-one (5i)

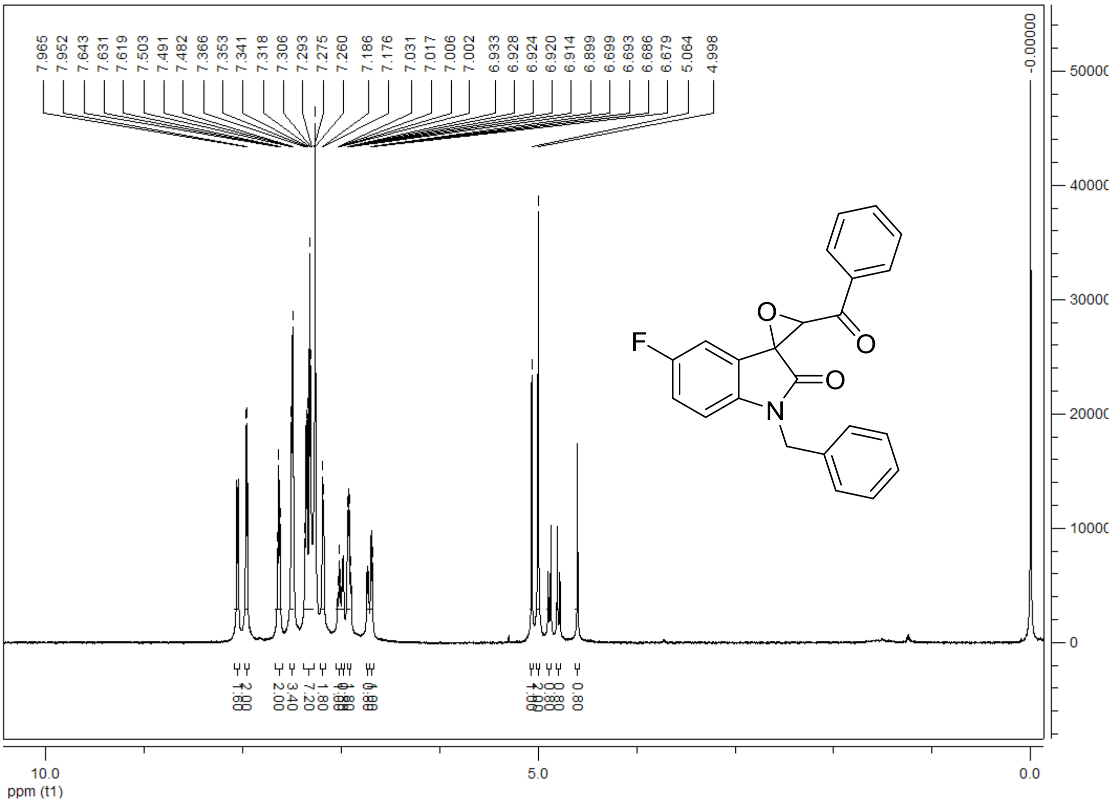

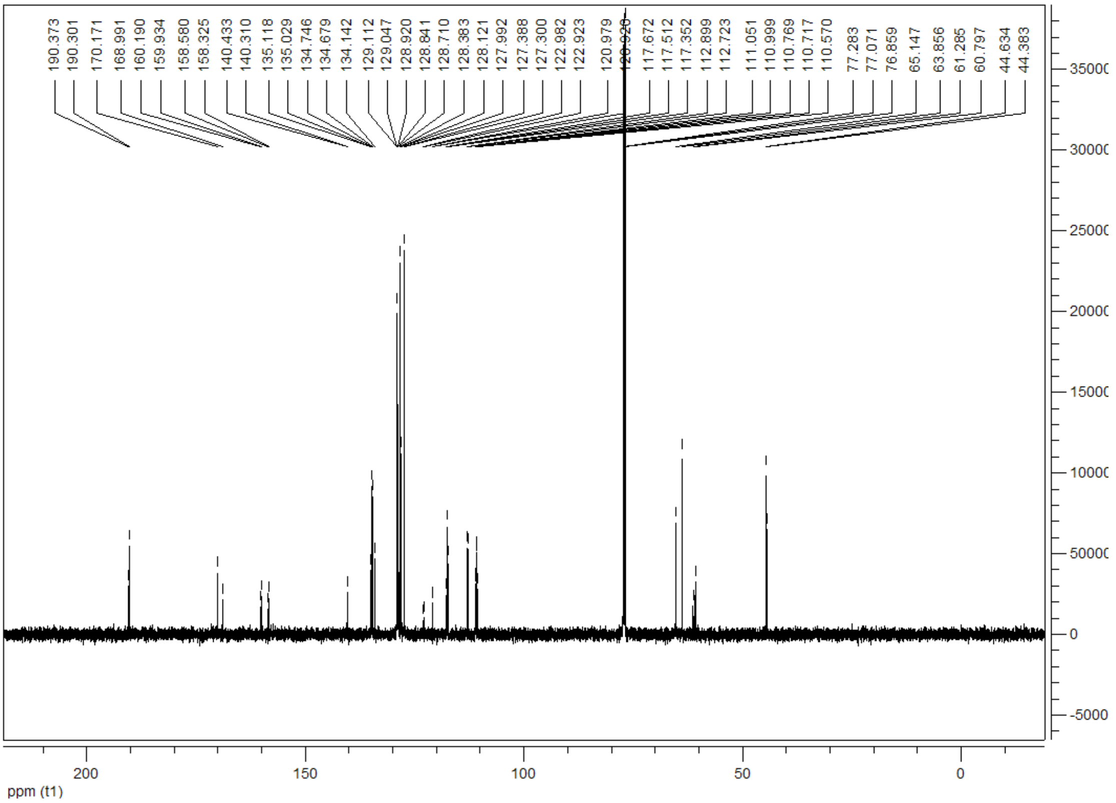

1-benzyl-3'-(4-chlorobenzoyl)-5-fluorospiro[indoline-3,2'-oxiran]-2-one (5j)

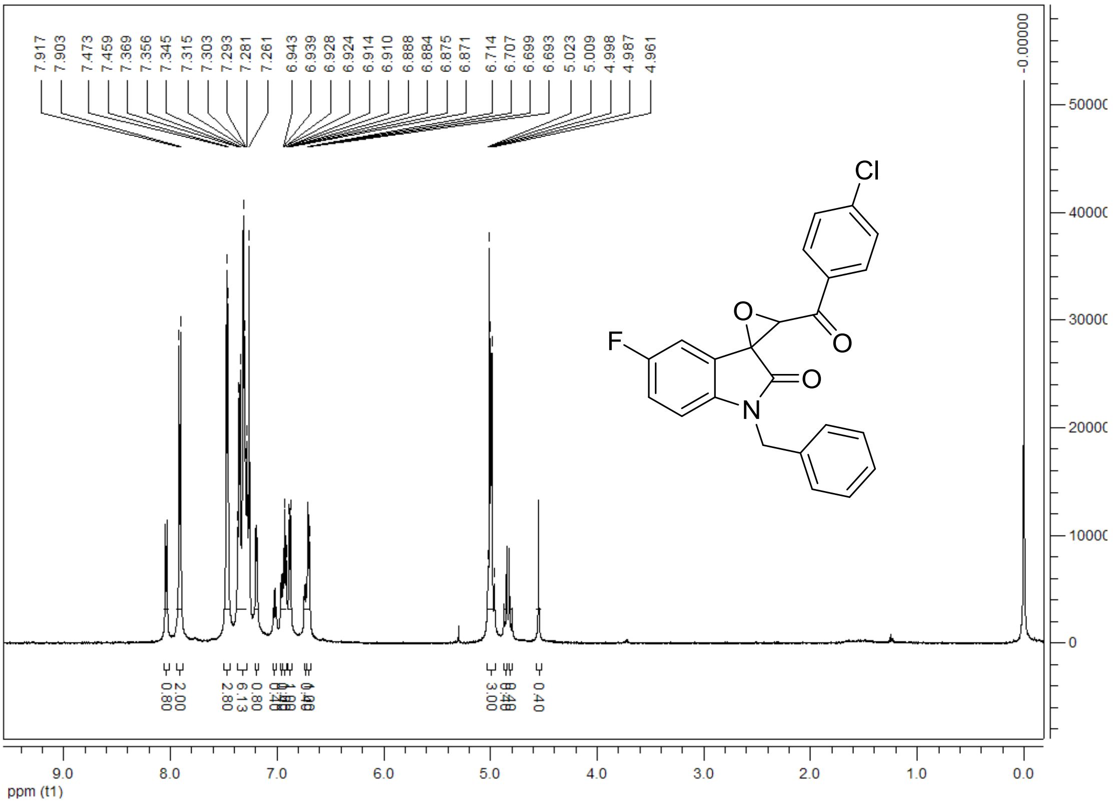

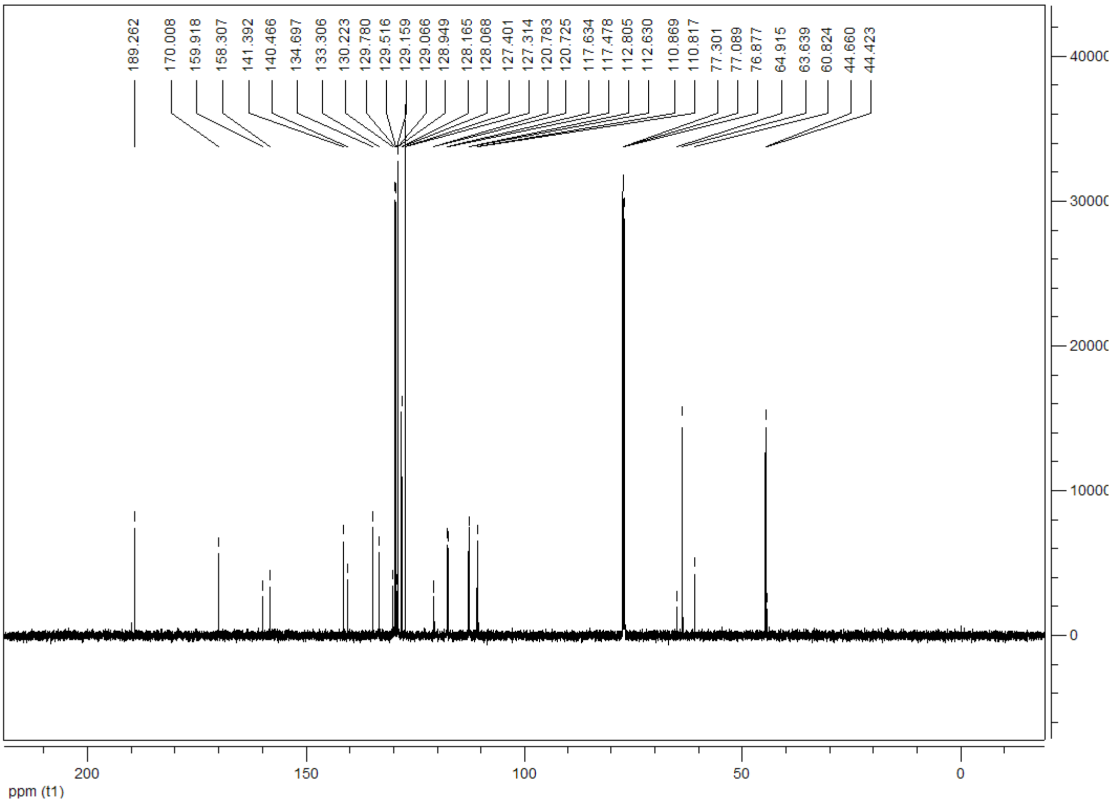

3'-benzoyl-1-butyl-5-fluorospiro[indoline-3,2'-oxiran]-2-one (5k)

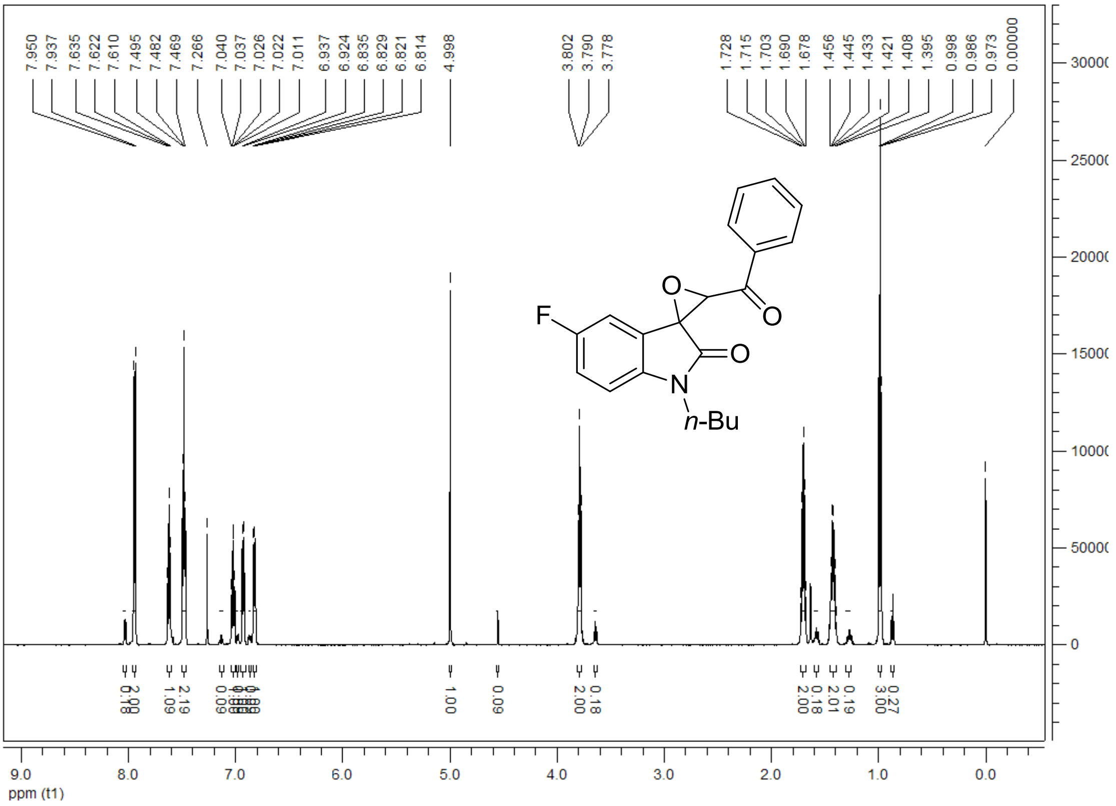

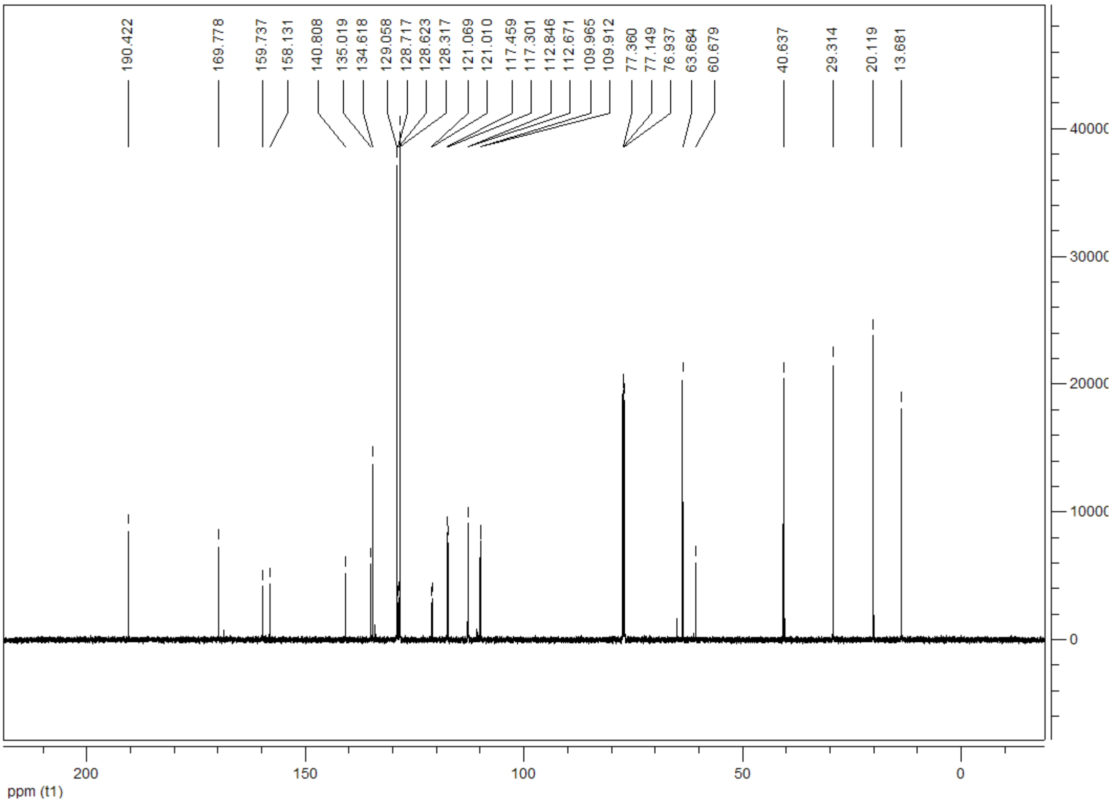

1-butyl-3'-(4-chlorobenzoyl)-5-fluorospiro[indoline-3,2'-oxiran]-2-one (5l)

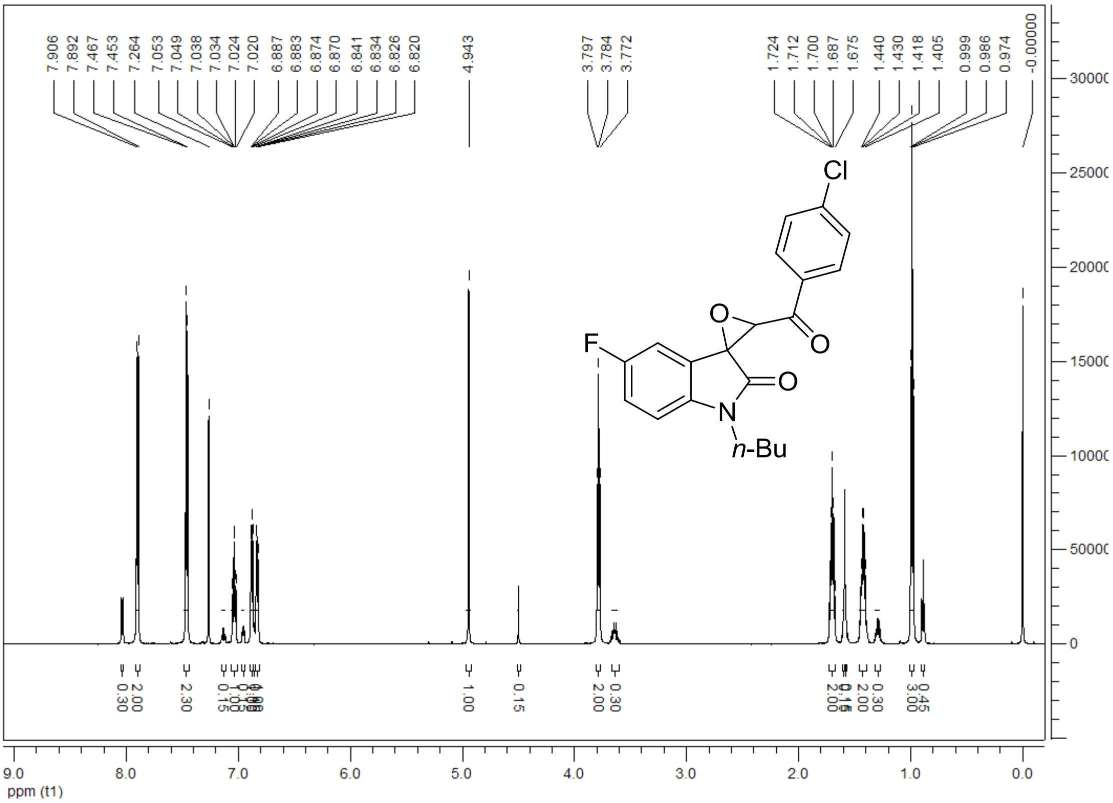

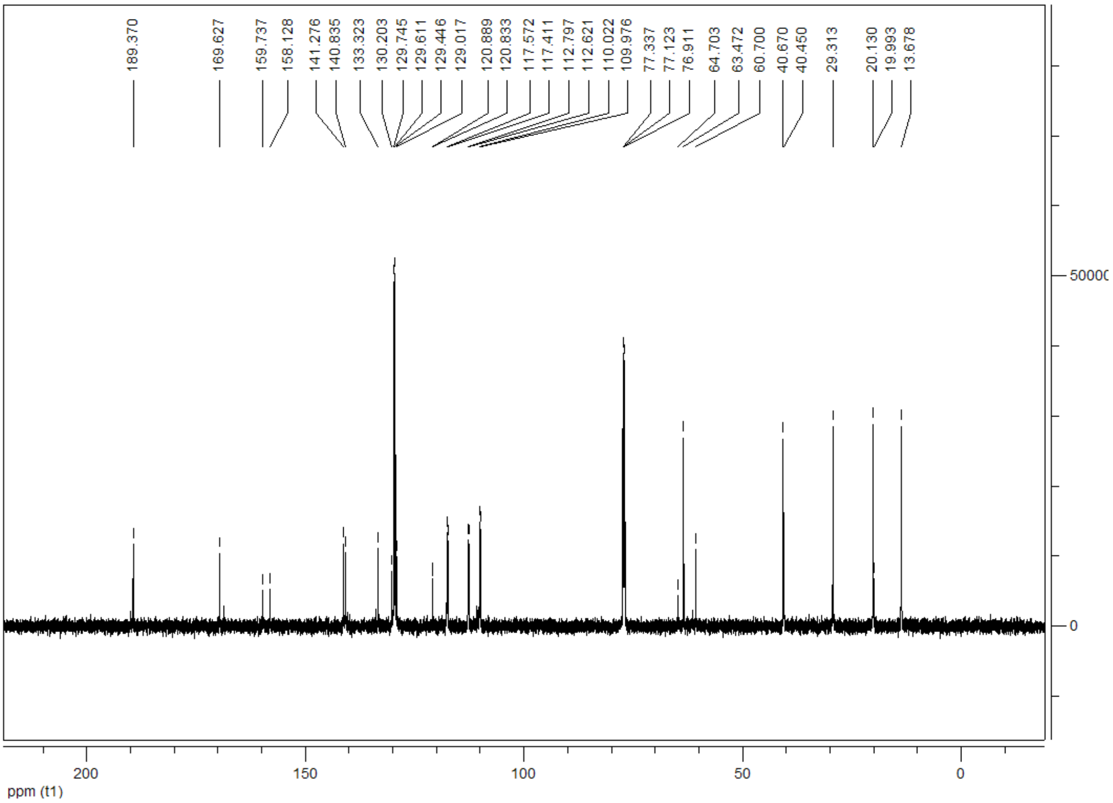

3'-benzoyl-1-benzyl-5-chlorospiro[indoline-3,2'-oxiran]-2-one (5m)

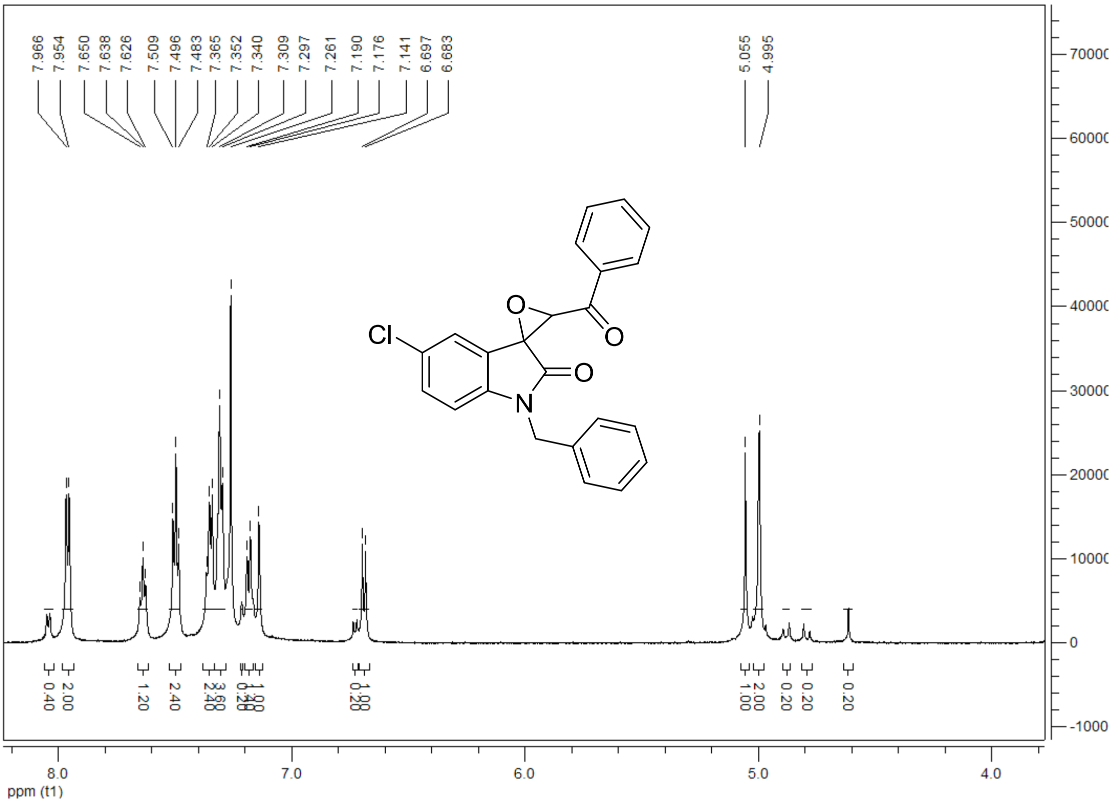

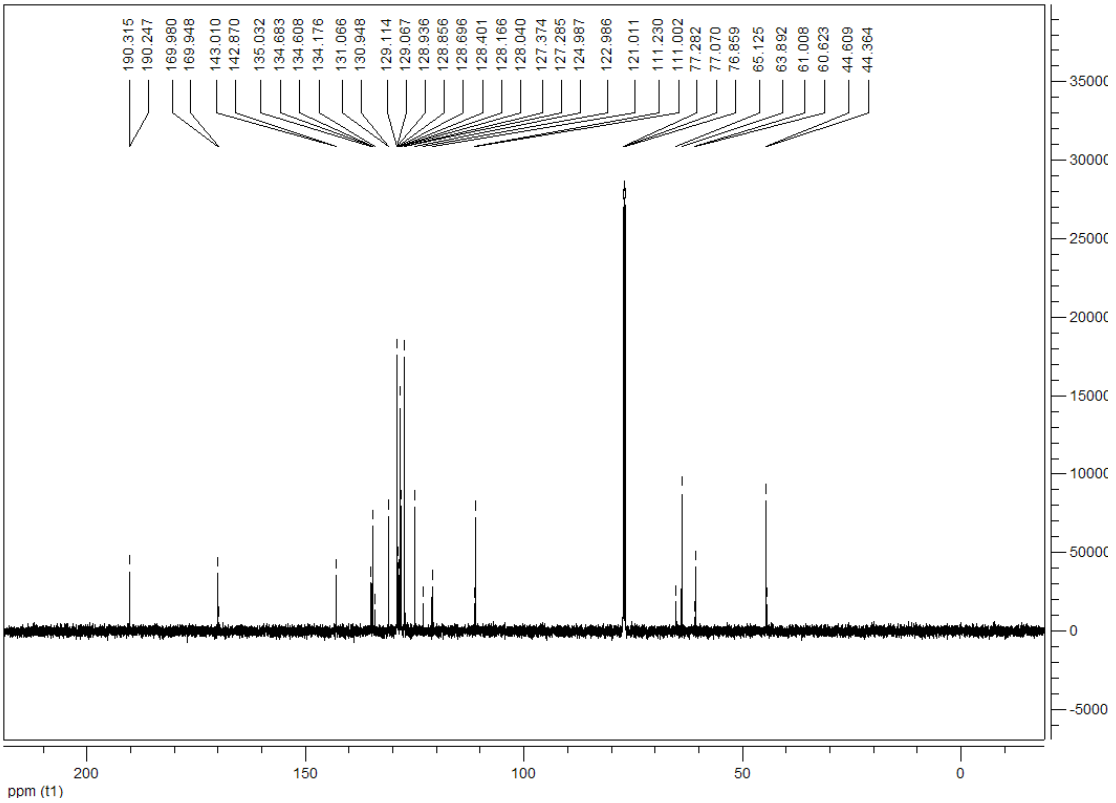

1-benzyl-5-chloro-3'-(4-chlorobenzoyl)spiro[indoline-3,2'-oxiran]-2-one (5n)

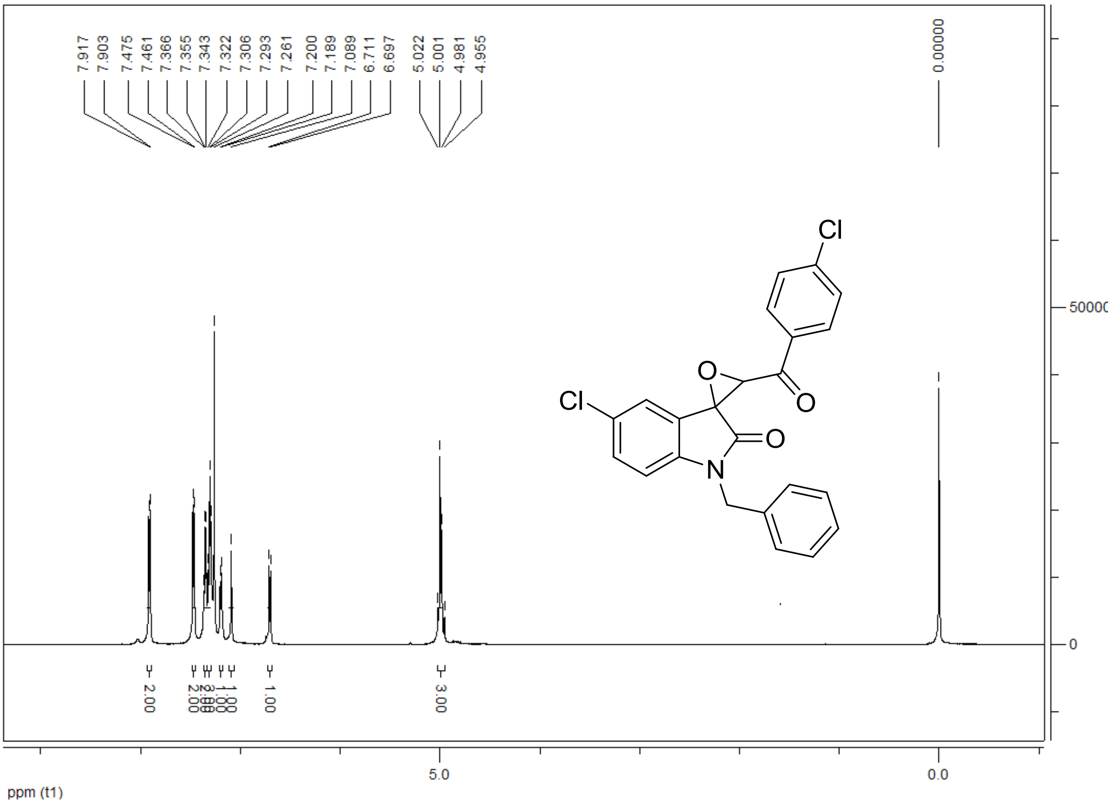

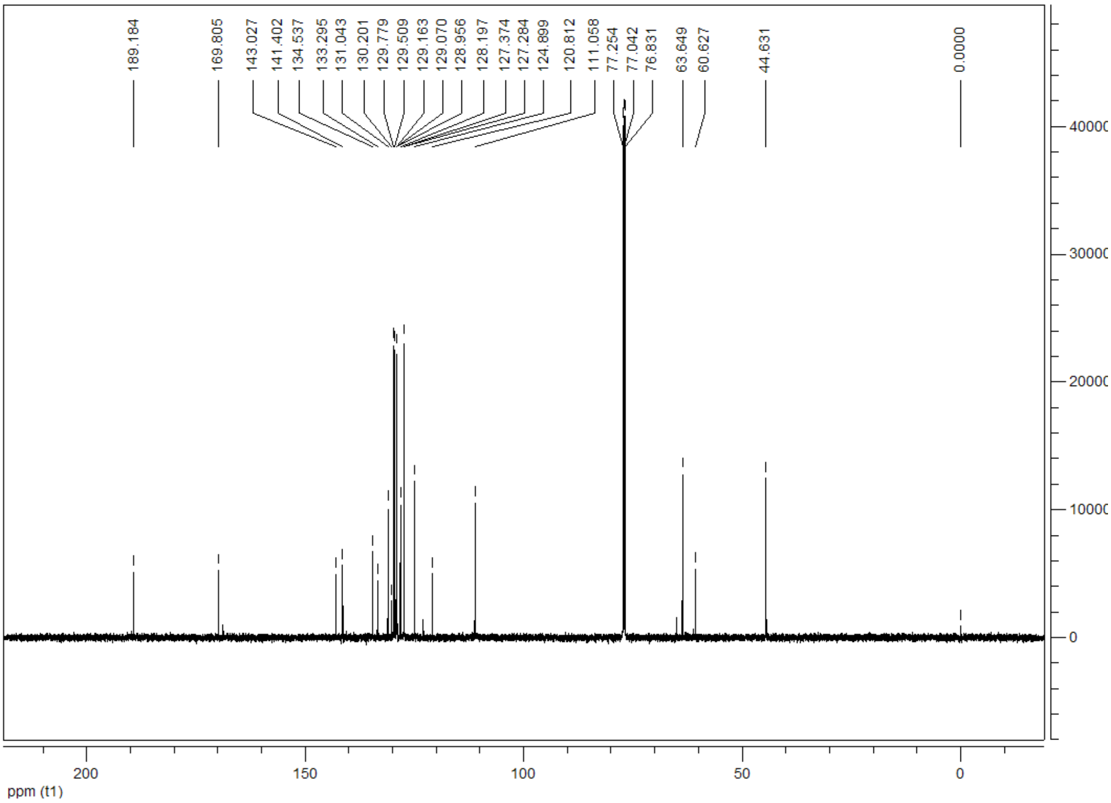

3'-benzoyl-1-butyl-5-chlorospiro[indoline-3,2'-oxiran]-2-one (5o)

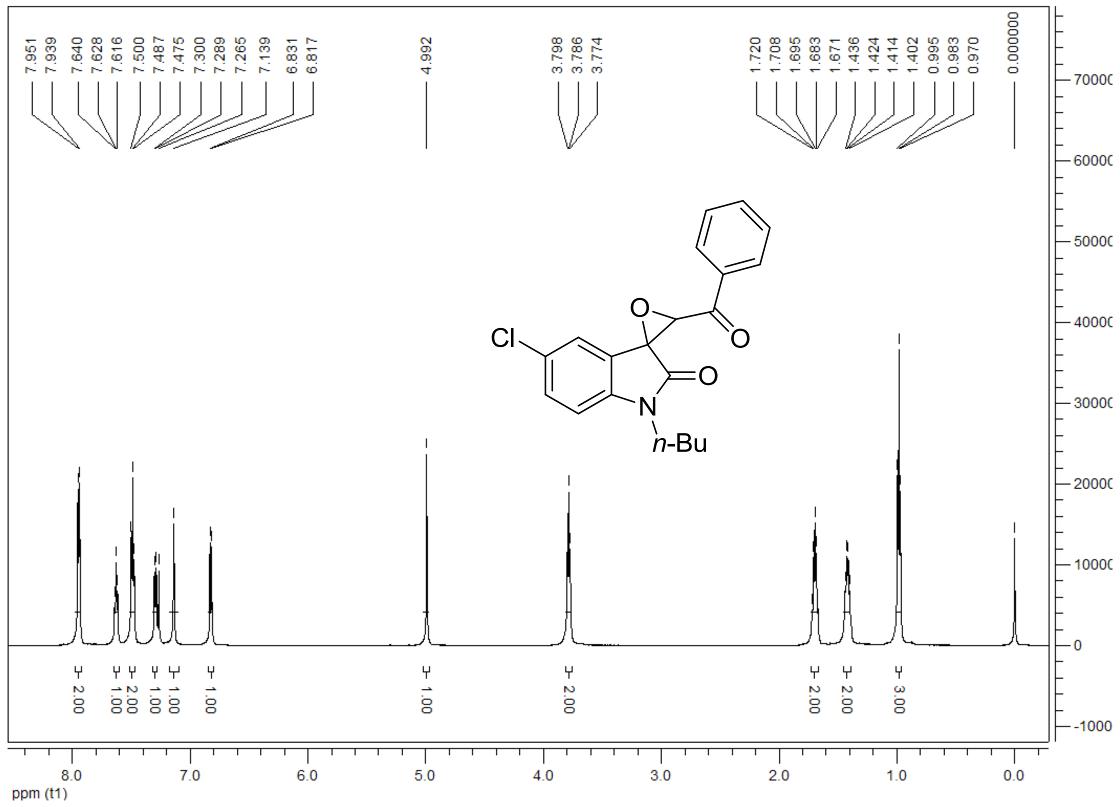

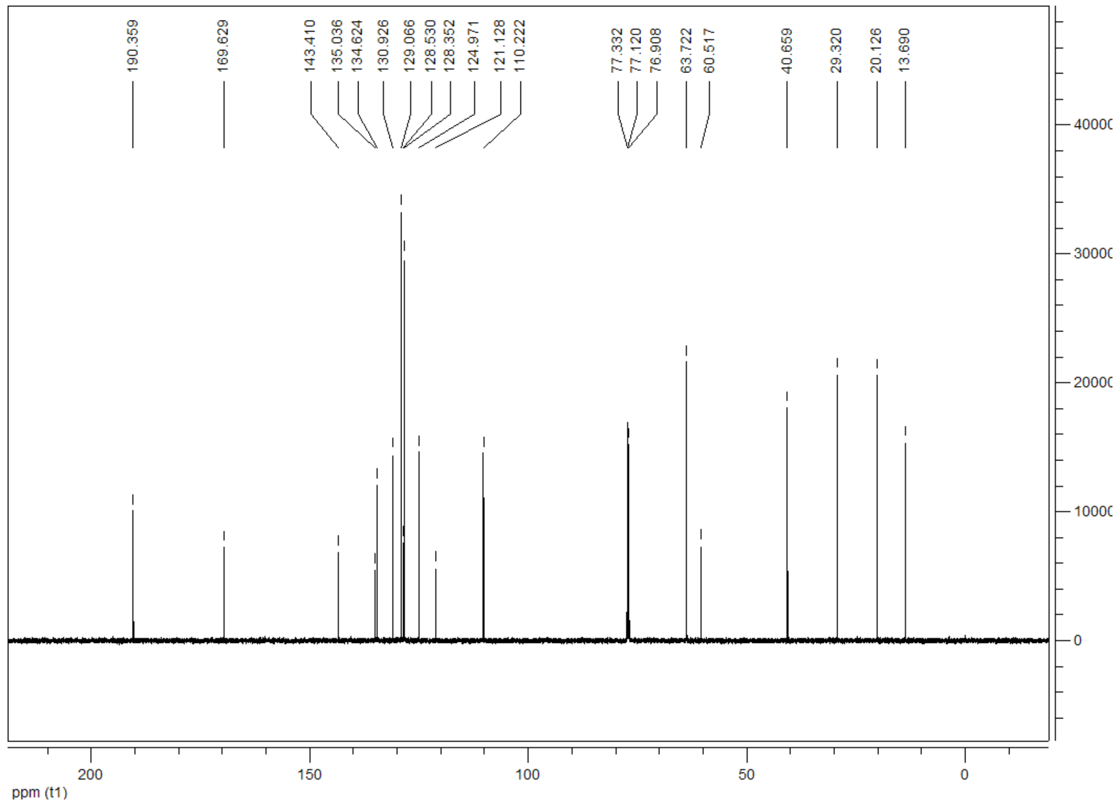

1-butyl-5-chloro-3'-(4-chlorobenzoyl)spiro[indoline-3,2'-oxiran]-2-one (5p)

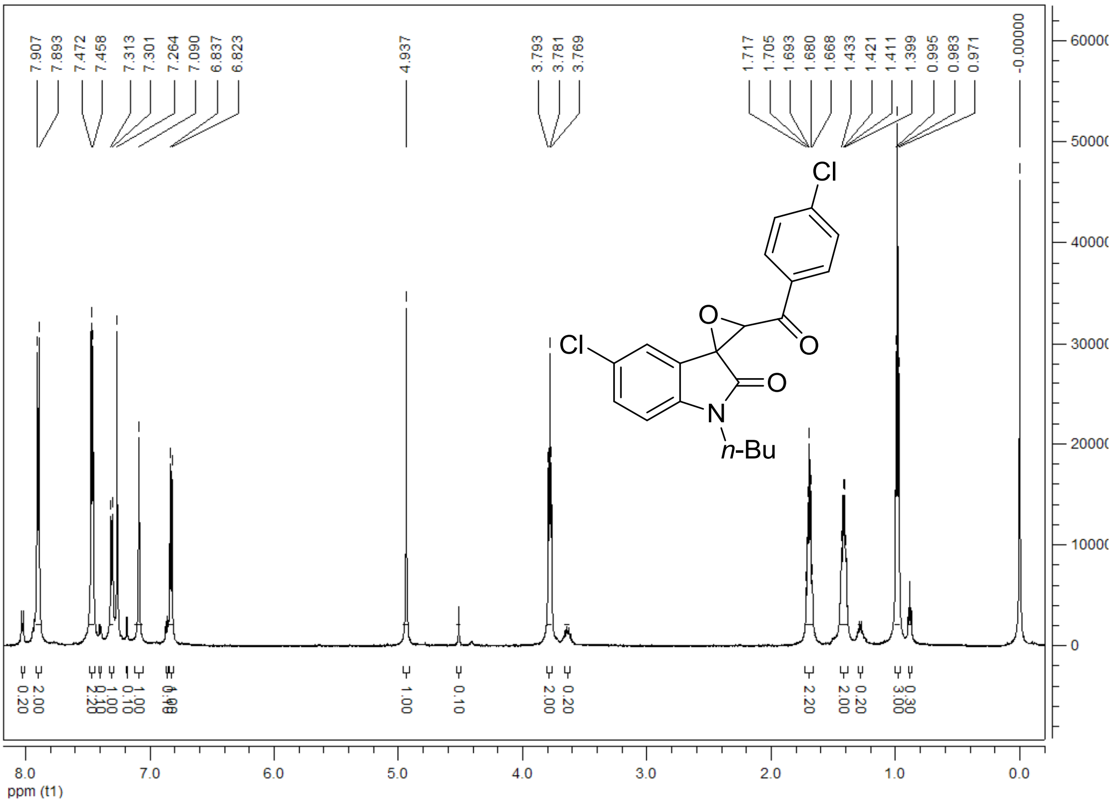

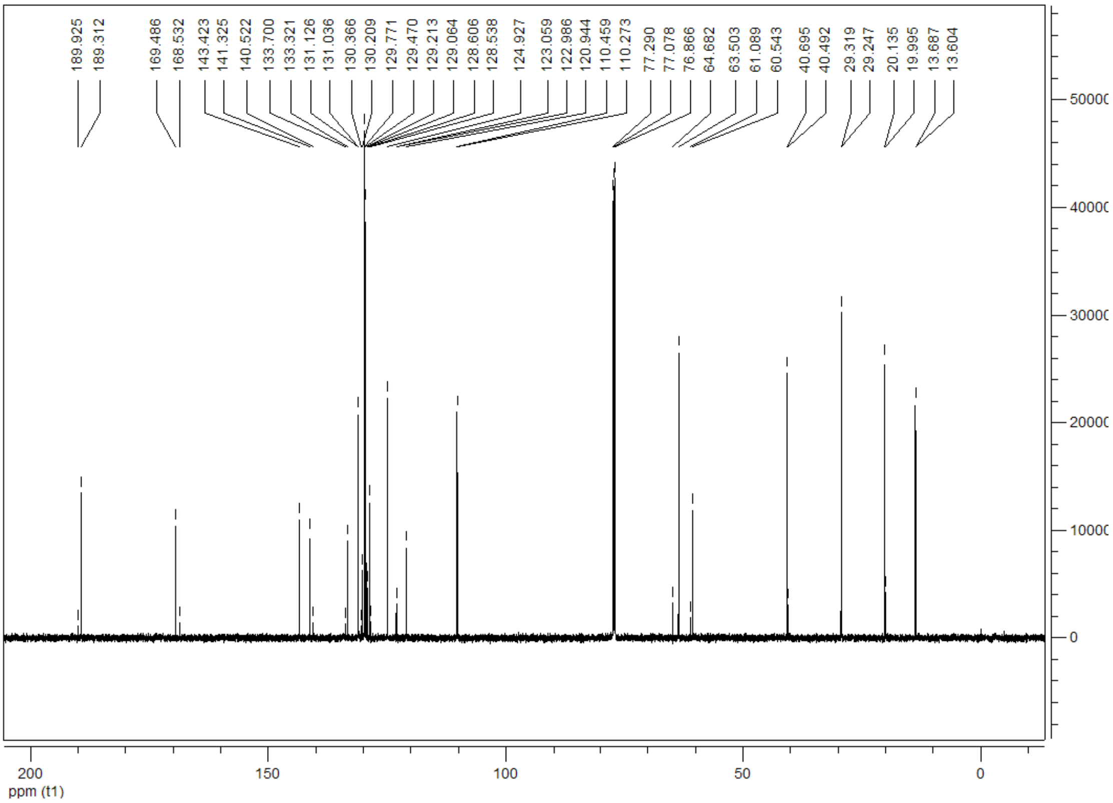

Supplement: File 1 — Experimental details and spectroscopic data. [file Beilstein_J_Org_Chem-09-918-s001.pdf]
